# Two distinct modes of DNMT1 recruitment ensure stable maintenance DNA methylation

**DOI:** 10.1038/s41467-020-15006-4

**Published:** 2020-03-06

**Authors:** Atsuya Nishiyama, Christopher B. Mulholland, Sebastian Bultmann, Satomi Kori, Akinori Endo, Yasushi Saeki, Weihua Qin, Carina Trummer, Yoshie Chiba, Haruka Yokoyama, Soichiro Kumamoto, Toru Kawakami, Hironobu Hojo, Genta Nagae, Hiroyuki Aburatani, Keiji Tanaka, Kyohei Arita, Heinrich Leonhardt, Makoto Nakanishi

**Affiliations:** 1 Division of Cancer Cell Biology, The Institute of Medical Science, The University of Tokyo, 4-6-1 Shirokanedai, Minato-ku, Tokyo, Japan; 2 Department of Biology II and Center for Integrated Protein Science Munich (CIPSM), Human Biology and BioImaging, Ludwig-Maximilians-Universität München, 82152 Planegg-Martinsried, Germany; 3 Structure Biology Laboratory, Graduate School of Medical Life Science, Yokohama City University, 1-7-29, Suehiro-cho, Tsurumi-ku, Yokohama, Kanagawa Japan; 4 Laboratory of Protein Metabolism, Tokyo Metropolitan Institute of Medical Science, 2-1-6 Kamikitazawa, Setagaya-ku, Tokyo, Japan; 5 Laboratory of Protein Organic Chemistry, Institute for Protein Research, Osaka University, Suita, Osaka Japan; 6 The Research Center for Advanced Science and Technology, University of Tokyo, 4-6-1 Komaba, Meguro-ku, Tokyo, Japan

**Keywords:** SAXS, DNA methylation

## Abstract

Stable inheritance of DNA methylation is critical for maintaining differentiated phenotypes in multicellular organisms. We have recently identified dual mono-ubiquitylation of histone H3 (H3Ub2) by UHRF1 as an essential mechanism to recruit DNMT1 to chromatin. Here, we show that PCNA-associated factor 15 (PAF15) undergoes UHRF1-dependent dual mono-ubiquitylation (PAF15Ub2) on chromatin in a DNA replication-coupled manner. This event will, in turn, recruit DNMT1. During early S-phase, UHRF1 preferentially ubiquitylates PAF15, whereas H3Ub2 predominates during late S-phase. H3Ub2 is enhanced under PAF15 compromised conditions, suggesting that H3Ub2 serves as a backup for PAF15Ub2. In mouse ES cells, loss of PAF15Ub2 results in DNA hypomethylation at early replicating domains. Together, our results suggest that there are two distinct mechanisms underlying replication timing-dependent recruitment of DNMT1 through PAF15Ub2 and H3Ub2, both of which are prerequisite for high fidelity DNA methylation inheritance.

## Abstract (web summary)

Ubiquitylation of histone H3 (H3Ub2) by UHRF1 recruits DNMT1 to chromatin, which is essential for DNA methylation inheritance. Here, the authors provide evidence that there are two distinct mechanisms underlying replication timing-dependent recruitment of DNMT1 through PAF15Ub2 and H3Ub2, both of which are required for high fidelity DNA methylation inheritance.

## Introduction

DNA cytosine methylation is a conserved epigenetic modification essential for embryonic development, transcriptional regulation, and genome stability^1^. In higher eukaryotes, individual differentiated cells possess unique DNA methylation patterns that determine their cellular phenotypes. Therefore, the DNA methylation pattern must be precisely maintained in coordination with DNA replication during S phase^2^. DNA methyltransferase 1 (DNMT1) contains multiple functional domains, including a replication foci targeting sequence (RFTS), an unmethylated CpG DNA-binding CXXC domain, two bromo-adjacent homology domains, and a C-terminal catalytic domain^3^. The RFTS domain is not only critical for DNMT1 recruitment to DNA methylation sites^4^ but also functions as an auto-inhibitory domain of DNMT1^5,6^.

The recruitment of DNMT1 to DNA methylation sites requires UHRF1, an E3 ubiquitin ligase^7,8^. UHRF1 recognizes specific epigenetic modifications on DNA strands and histone H3 tails through its SET- and RING-associated (SRA) domain and tandem Tudor domain (TTD)–plant homeodomain (PHD), respectively^9–14^. The former binds to hemi-methylated DNA, while the latter recognizes N-terminal ^1^ARTK^4^ residues and tri-methylated Lys9 of H3 (H3K9me3). The TTD domain also contributes to the interaction between UHRF1 and DNA ligase 1^15,16^. Furthermore, the E3 ubiquitin ligase activity of UHRF1 plays an essential role in DNMT1 recruitment to DNA methylation sites^17,18^, and is enhanced by association with hemi-methylated DNA and H3K9me3^19,20^. The UBL domain of UHRF1 also stimulates the E3 ligase activity of UHRF1 through its interaction with E2 ubiquitin-conjugating enzyme UbcH5a/UBE2D1^21,22^. We and others have recently reported that UHRF1-mediated dual mono-ubiquitylation of histone H3 (H3Ub2) on lysine residues 14, 18, and 23 plays a role in the RFTS-dependent recruitment of DNMT1 and its enzymatic activation, ensuring the high fidelity of DNA maintenance methylation^18,23,24^. DNMT1-bound USP7 also accumulates at DNA methylation sites^25,26^ and contributes to efficient DNA methylation by deubiquitylation of histone H3 and DNMT1^25–28^.

However, the existence of two distinct modes of DNMT1 recruitment to hemi-methylation sites, one coupled with and the other uncoupled from DNA replication machinery, has previously been suggested by the finding that DNMT1 co-localizes with LIG1 foci in early and mid-S phase but not in late S phase^29^. While H3Ub2 serves as one mark of DNMT1 recruitment, how this mark is coordinated with S-phase progression remains unknown.

In this report, we identify dual mono-ubiquitylation of PAF15 (PAF15Ub2) as a molecular mark coupling DNMT1 recruitment with DNA replication. During DNA replication, DNMT1 predominantly utilizes PAF15Ub2. When the PAF15-dependent mechanism is perturbed, DNMT1 utilizes H3Ub2, suggesting that H3Ub2 functions as a backup system for the maintenance of DNA methylation.

## Results

### Ubiquitylated PAF15 specifically binds replicating chromatin

Given that the ubiquitin ligase activity of UHRF1 and the ubiquitin binding activity of DNMT1 are essential for the recruitment of DNMT1 to hemi-methylated DNA sites, we speculated that factors associated with the DNA replication machinery also utilize ubiquitin signals to recruit DNMT1. To identify factors capable of binding DNMT1 in a ubiquitin signal-dependent manner, we used ubiquitin vinyl sulfone (UbVS) treatment, a pan-deubiquitylation enzyme inhibitor^30^, to specifically enrich for ubiquitylated proteins in cell-free *Xenopus* extracts. In brief, pretreatment of egg extracts with UbVS inhibits ubiquitin turnover and results in an almost complete depletion of free ubiquitin, leading to the inhibition of both ubiquitylation and deubiquitylation pathways^31^. Thus, the addition of recombinant ubiquitin to UbVS-treated extracts specifically enhanced ubiquitin signals, including UHRF1-mediated histone H3 ubiquitylation^23,25^. Chromatin lysates from UbVS-treated extracts in the presence (UbVS+Ub) or absence (UbVS) of free ubiquitin were subjected to a pull-down experiment using recombinant Flag-tagged wild-type *Xenopus* DNMT1 (rxDNMT1^WT^) purified from insect cells (Supplementary Fig. 1a–c). As reported previously^17,23^, rxDNMT1^WT^ specifically interacted with H3Ub2 in denatured chromatin lysates (Supplementary Fig. 1d, +sodium dodecyl sulfate (+SDS)). In native chromatin lysates, rxDNMT1^WT^ interacted with H3Ub2 as well as with unmodified and mono-ubiquitylated histone H3 (Supplementary Fig. 1d, −SDS), suggesting that indirect binding is also preserved under this condition.

**Fig. 1:**
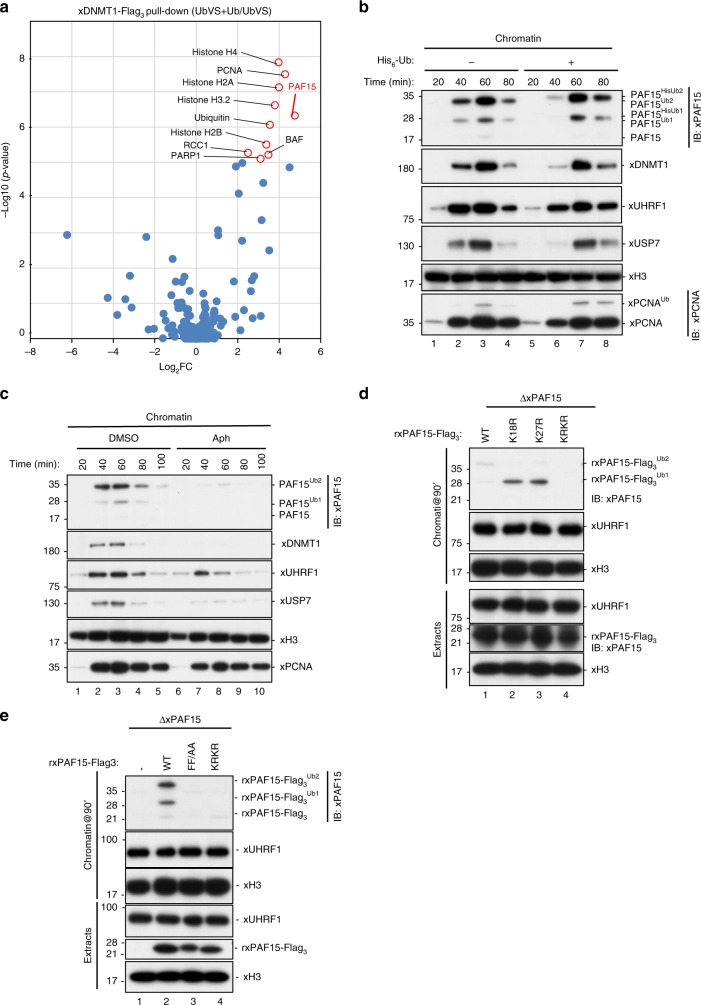
Dual mono-ubiquitylated PAF15 (PAF15Ub2) specifically binds to replicating chromatin. **a** xDNMT1 pull-downs from native chromatin extracts were analyzed by LC-MS/MS. The volcano plot summarizes the quantitative results and highlights the interacting proteins enriched upon addition of ubiquitin to UbVS-treated extracts. **b** *Xenopus* interphase egg extracts were added with sperm chromatin and incubated in the absence or presence of His_6_-ubiquitin (58 μM final). Chromatin-bound proteins were isolated and analyzed by immunoblotting using the indicated antibodies. For PAF15 levels in the extracts, see Supplementary Fig. 1f. **c** Interphase egg extracts were added with sperm chromatin and incubated in the presence of 15 μM aphidicolin (Aph) or in its absence (DMSO). Chromatin-bound proteins were isolated and analyzed by immunoblotting using the indicated antibodies. **d** PAF15-deleted extracts were supplemented with wild-type xPAF15-Flag_3_ and its variants (K18R, K27R, and K18R/K27R). After the addition of sperm chromatin, chromatin-bound proteins were isolated and analyzed by immunoblotting using the indicated antibodies. The extracts were also analyzed by immunoblotting. **e** PAF15-depleted extracts were supplemented with wild-type xPAF15-Flag_3_, its PIP-box mutant (FF/AA), or K18R/K27R mutant (KRKR). After the addition of sperm chromatin, chromatin-bound proteins were isolated and analyzed by immunoblotting using the indicated antibodies. The extracts were also analyzed by immunoblotting. Source data are provided as a Source Data file.

We next subjected the pull-downs of rxDNMT1^WT^ or endogenous xDNMT1 from native chromatin lysates to mass spectrometric analysis. We identified 2840 unique peptides (including 26 ubiquitylated and 17 phosphorylated peptides), which mapped to 303 protein groups in chromatin lysates from UbVS-treated extracts in the presence (UbVS+Ub) or absence (UbVS) of free ubiquitin (Supplementary Data 1). Of these xDNMT1-interacting chromatin proteins, 24 were highly enriched in the xDNMT1 pull-downs in response to the addition of ubiquitin to UbVS-treated extracts (log_2_fold-change >2, *p* value < 0.05; Fig. 1a, Supplementary Data 1). We also found an enrichment of eight ubiquitylated and two phosphorylated peptides in the data set (Supplementary Data 2 and 3). Histone H3 variants were identified, together with other histone proteins, validating the interactors (Fig. 1a and Supplementary Data 1). Among the identified proteins, we focused on PAF15, one of the most highly enriched proteins (log_2_fold-change = 4.75), because it was reported to be associated with both proliferating cell nuclear antigen (PCNA) and DNMT1^32^, and was targeted for dual mono-ubiquitylation at its H3-like N-terminal sequence during S phase in human cells (see also Supplementary Fig. 1e)^33^, suggesting that this interaction is conserved among vertebrates and is regulated in a ubiquitin signal-dependent manner.

The addition of sperm chromatin to cell-free *Xenopus* interphase egg extracts induces the assembly of replication-competent nuclei and a single round of DNA replication. Under these conditions, DNA replication typically begins approximately 40 min after sperm addition. After the completion of DNA replication and maintenance of DNA methylation, many chromosomal replication regulators including DNMT1 and UHRF1 dissociate from the chromatin^17,34^. Using interphase egg extracts, we first examined the S-phase chromatin binding and ubiquitylation of xPAF15 along with the proteins involved in maintenance of DNA methylation. We found that slow migrating forms of xPAF15 bound to chromatin (Fig. 1b) in line with results using human cells^33^. When an excess amount of recombinant His_6_-tagged ubiquitin was added to the egg extracts (Supplementary Fig. 1f), the slowly migrating xPAF15 bands were further upshifted (Fig.1b), suggesting that these slow forms correspond to ubiquitylated xPAF15 (Fig. 1b). The binding kinetics of xPAF15 were generally similar to those of xDNMT1 or xUSP7 over the same time course (Fig. 1b). The chromatin binding of xPAF15, as well as that of xDNMT1 and xUSP7, were lost in the presence of aphidicolin (Fig.1c), a DNA polymerase inhibitor, suggesting that the chromatin binding of these proteins requires ongoing DNA synthesis.

In order to explore the role of xPAF15 ubiquitylation in xDNMT1 recruitment, we first identified ubiquitylation sites in xPAF15. We determined that the highly conserved lysine residues, K15 and K24 of human PAF15 (hPAF15), correspond to K18 and K27 of xPAF15 (Supplementary Fig. 1e)^33^. Interphase egg extracts depleted of endogenous xPAF15 were supplemented with recombinant xPAF15-Flag_3_ purified from insect cells, then sperm chromatin was added. Wild-type recombinant xPAF15 (rxPAF15^WT^), as well as the endogenous xPAF15, underwent ubiquitylation and bound to chromatin during DNA replication (Fig. 1d). In contrast, mutant xPAF15 with a substitution of lysine to arginine at both K18 and K27 (KRKR) failed to do so (Fig. 1d). Single xPAF15 mutants with the substitution at either site (K18R or K27R) underwent mono-ubiquitylation and retained the chromatin-binding activity (Fig. 1d). Next, we examined how depletion of free ubiquitin from egg extracts affects xPAF15 chromatin binding. Pretreatment of egg extracts with UbVS completely suppressed the chromatin loading of both xPAF15 and xDNMT1, whereas xUHRF1 chromatin binding was maintained (Supplementary Fig. 1g, h). Addition of free ubiquitin to UbVS-treated extracts efficiently restored PAF15 chromatin binding (Supplementary Fig. 1h). These results demonstrate that the mono-ubiquitylation of xPAF15 at K18 and/or K27 is important for stable xPAF15 chromatin association.

We then examined the role of xPCNA binding in xPAF15 chromatin loading. xPAF15 formed a stable complex with xPCNA in the egg extracts (Supplementary Fig. 1i) as it did in human cells^32,33^. Substitution of phenylalanine with alanine at two conserved residues within the PCNA interacting peptide motif (PIP-box) of glutathione S-transferase (GST)-xPAF15 (FF/AA) abolished its interaction with xPCNA (Supplementary Fig. 1j). Although the WT rxPAF15 bound to the chromatin (Fig. 1e), the rxPAF15^FF/AA^ mutant failed to do so, as did the rxPAF15^KRKR^ mutant (Fig. 1e). These results suggest that xPAF15 chromatin loading requires interaction with xPCNA.

### UHRF1 recognizes the N-terminal H3-like sequence of PAF15

**Fig. 2:**
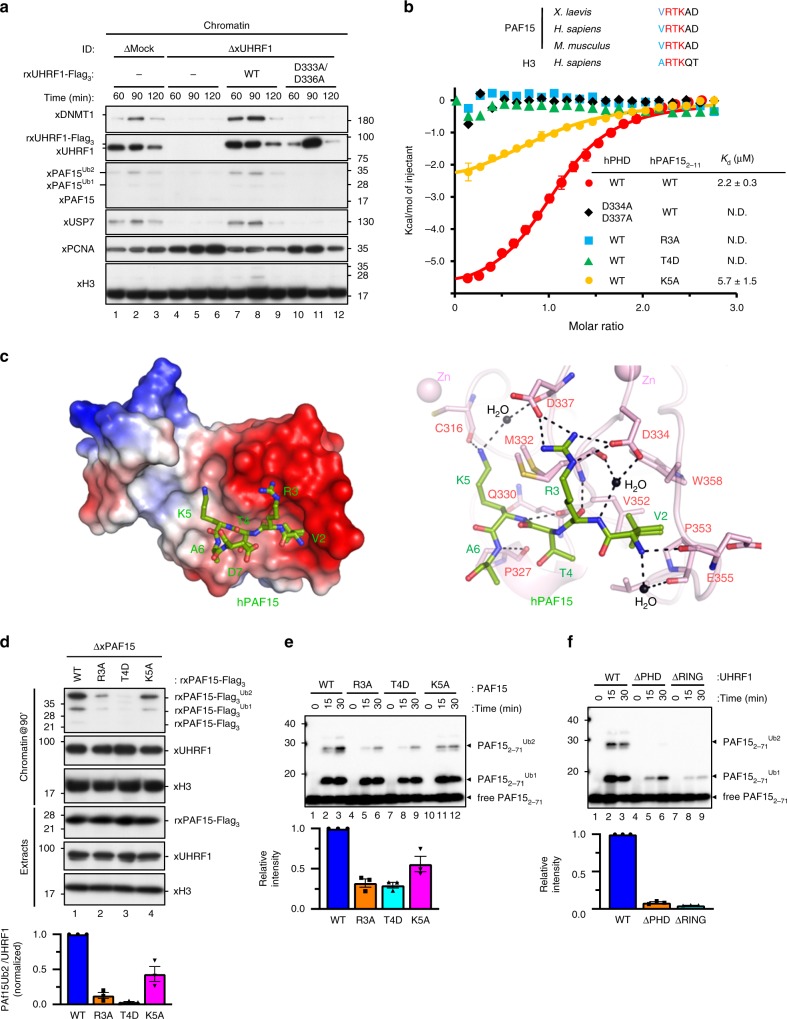
UHRF1 recognizes and ubiquitylates the N-terminal H3-like sequence of PAF15. **a** Mock-depleted or UHRF1-depleted extracts were supplemented with the indicated recombinant proteins (wt/D333A/D336A xUHRF1; see “Methods”) and chromatin was isolated. Chromatin-bound proteins were analyzed by immunoblotting using the indicated antibodies. For the protein levels of each protein in the extracts, see Supplementary Fig. 2a. **b** Comparison of the N-terminal sequence of PAF15 and histone H3 across different species. Residues mutated in the PAF15 mutants used in this study are shaded. Superimposition of plots of enthalpy changes in the interaction between hPHD and hPAF15_2-11_ peptides by ITC measurement. **c** Recognition of the N-terminus of hPAF15 by hPHD. The left panel shows the crystal structure of PHD in complex with hPAF15. hPHD as a surface model with electrostatic potential (red, negative; blue, positive). The right panel shows recognition of PAF15 N-terminus (green stick model) by hPHD (pink stick model). Hydrogen bonds and water molecules are shown as black lines and balls, respectively. **d** PAF15-deleted extracts were supplemented with wild-type PAF15-Flag_3_ and its variants (R3A, T4D, and K5A). After the addition of sperm chromatin, chromatin-bound proteins were isolated after 90 min and analyzed by immunoblotting using the indicated antibodies. The level of PAF15Ub2 on chromatin was quantified for each set of conditions as explained in the “Methods” section. **e**, **f** In vitro ubiquitylation assay using the indicated hUHRF1 E3-ligases and hPAF15 substrates. Lower panels show the relative intensity of the band corresponding to dual mono-ubiquitylated PAF15. Bars represent the means of three independent experiments with SEM. Source data are provided as a Source Data file.

We next examined the requirement of the E3 ubiquitin ligase xUHRF1 for xPAF15 ubiquitylation and chromatin loading. As we demonstrated previously, immunodepletion of UHRF1 from egg extracts inhibited DNMT1 recruitment and chromatin association of xPAF15 (Fig. 2a, Supplementary Fig. 2a). Addition of recombinant WT xUHRF1 (rxUHRF1^WT^) purified from insect cells to UHRF1-depleted extracts rescued the chromatin loading of xPAF15 (Fig. 2a, Supplementary Fig. 2a). We also tested the effect of recombinant xUHRF1 containing D333A/D336A, point mutations in the PHD finger that are expected to cause a loss of interaction with the histone H3 tail^13,14^. Strikingly, rxUHRF1^D333A/D336A^ did not support xPAF15 ubiquitylation and chromatin loading (Fig. 2a, Supplementary Fig. 2a), suggesting that UHRF1-PHD has a crucial role in the regulation of PAF15. In contrast, xDNMT1 depletion resulted in the accumulation of xUHRF1 and ubiquitylation of histone H3 on the chromatin (Supplementary Fig. 2b, c). Dual mono-ubiquitylated xPAF15 (xPAF15Ub2) also accumulated on the chromatin (Supplementary Fig. 2b, c). These effects were restored by the addition of rxDNMT1 (Supplementary Fig. 2b, c). Our results indicate that both xPAF15 ubiquitylation and its chromatin recruitment are xUHRF1 dependent.

The PHD finger of hUHRF1 (hPHD) has been shown to bind to N-terminal ^1^ARTK^4^ residues of histone H3^13,14^. Given that the N-terminal portion of PAF15 shares significant homology with the N-terminal tail of histone H3 (Fig. 2b), we reasoned that hPHD likely to bind the N-terminal portion of PAF15. Isothermal titration calorimetry (ITC) demonstrated that hPHD bound to human PAF15_2-11_ with *K*_d_ = 2.2 ± 0.3 μM. This value is comparable to that for the N-terminal histone H3 peptide (*K*_d_ = 1.5 ± 0.1 μM) (Fig. 2b, Supplementary Fig. 2d). In order to determine the binding mode of hPHD to PAF15, the crystal structure of hPHD bound to hPAF15_2-11_ was determined at 1.7 Å resolution (Table 1). The structure showed that the hPAF15_2-11_ peptide bound to the acidic surface of hPHD (Fig. 2c left), with the ^2^VRTK^5^ sequence of hPAF15_2-11_ being recognized in a manner similar to that of the ^1^ARTK^4^ of histone H3 (Supplementary Fig. 2e, f). The N-terminus of hPAF15_2-11_ formed a hydrogen bond with hPHD-E355, and hPAF15_2-11_-V2 was surrounded by the hydrophobic residues V352, P353, and W358 of the hPHD (Fig. 2c right). hPAF15_2-11_-R3 and -K5 formed an electrostatic interaction with hPHD-D334 and -D337 and a hydrogen bond with hPHD-C316, respectively (Fig. 2c right). The importance of the above interactions in the complex formation was further validated by mutation analysis. ITC data demonstrated that hPHD-D334A/D337A failed to bind to WT hPAF15_2-11_ while the WT hPHD was unable to bind to hPAF15_2-11_-R3A or -T4D (Fig. 2b). Consistently, rxPAF15^R3A^ and rxPAF15^T4D^ failed to bind to chromatin during S phase in xPAF15-depleted extracts (Fig. 2d). Highlighting the importance of the hPHD recognition of hPAF15, in vitro ubiquitylation assays revealed that UHRF1 D334A/D337A and PAF15 R3A or T4D mutations significantly decreased the ubiquitylation of hPAF15, as well as the UHRF1 H741A mutation that disrupted E3 activity (Fig. 2e, f). The PAF15 K5A mutation had only a small effect on chromatin binding, interaction with hPHD, and ubiquitylation (Fig. 2b, d, e). Together, these findings suggest that the PHD finger of UHRF1 is responsible for the association with the N-terminal end of PAF15 with a binding mode similar to that of histone H3 and plays a critical role in PAF15 ubiquitylation by UHRF1.

**Table 1:** Data collection and refinement statistics.

	PHD:PAF15 (PDB: 6IIW)
Data collection	
Beam line	PF-BL17A
Wavelength (Å)	0.98
Space group	*P*6_1_22
Cell dimensions	
* a*, *b*, *c* (Å)	36.7, 37.6, 220.2
Resolution (Å)	44.03–1.70 (1.73–1.70)^a^
* R*_sym_ or *R*_merge_ (%)	6.5 (49.7)^a^
* I*/σ(*I*)	27.5 (5.6)^a^
CC_1/2_	99.9 (97.6)^a^
Completeness (%)	100 (99.9)^a^
Redundancy	17.2 (17.6)^a^
Unique reflections	10,762 (538)
Refinement	
Resolution (Å)	36.69–1.70
No. of reflections	10,653
* R*_work_/*R*_free_ (%)	17.6/18.9
No. of atoms	
PHD	522
PAF15	51
Ion	4
Water	96
Average B factors (Å^2^)	
PHD	26.6
PAF15	26.4
Ion	19.4
Water	38.4
R.m.s. deviations	
Bond lengths (Å)	0.005
Bond angles (°)	0.928

^a^Values in parentheses are for the highest-resolution shell.

### PAF15Ub2 forms a complex with DNMT1

**Fig. 3:**
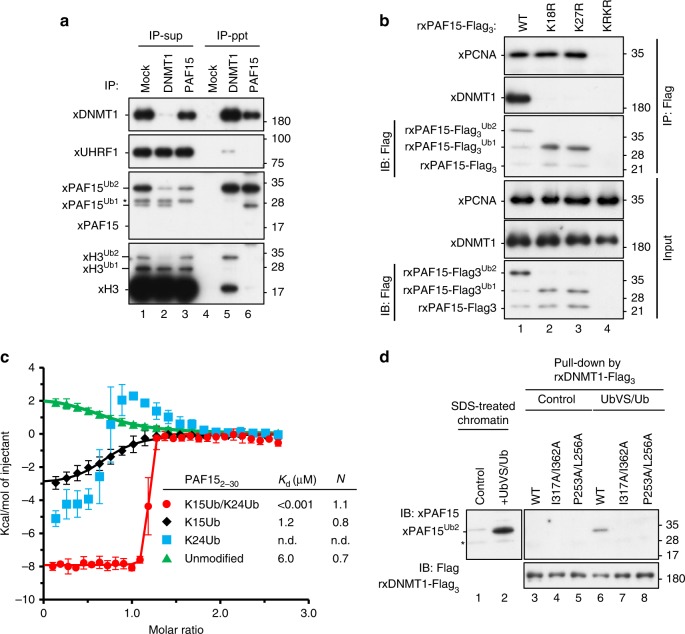
PAF15Ub2 forms a complex with DNMT1. **a** Reciprocal immunoprecipitation of PAF15 and DNMT1 from chromatin lysates. IP was performed with control (Mock), anti-xDNMT1 (DNMT1), or anti-xPAF15 (PAF15) antibody from chromatin lysates. Supernatants after immunoprecipitation (IP-sup) or immunoprecipitates (IP-ppt) were analyzed by immunoblotting using the indicated antibodies. **b** Sperm chromatin was replicated in interphase egg extracts containing xPAF15-Flag_3_ [wild-type, K18R, K27R, or K18RK27R (KRKR)]. Isolated and solubilized chromatin proteins were subjected to immunoprecipitation using anti-Flag antibodies. The resultant immunoprecipitates were analyzed by immunoblotting using the indicated antibodies. **c** Superimposition of plots of enthalpy changes in the interaction between hRFTS and hPAF15_2-30_ or its ubiquitylated analogs by ITC measurement. **d** Pull-down of ubiquitylated PAF15 from denatured chromatin extracts using recombinant wild-type xDNMT1-Flag_3_ and its ubiquitin-binding mutants (P253AL256A or I317AI362A). Source data are provided as a Source Data file.

We recently reported that DNMT1 specifically binds to H3Ub2 via the RFTS domain^23^. Given the similarity of PAF15 to the H3 tail and its ability to be dual mono-ubiquitylated, we asked whether PAF15 is also specifically recognized by the RFTS domain of DNMT1. Immunoprecipitation (IP)–western blotting analysis using solubilized chromatin revealed that the majority of xPAF15Ub2 bound to xDNMT1 (Fig. 3a, Supplementary Fig. 3a). Although xDNMT1 bound to H3Ub2, xPAF15Ub2 failed to do so (Fig. 3a), suggesting that xDNMT1-H3Ub2 and xDNMT1-xPAF15Ub2 complexes are mutually exclusive. Similar results were obtained using UbVS+Ub-treated egg extracts (Supplementary Fig. 3b). Next, we examined the DNMT1 binding of ubiquitylation-deficient xPAF15 mutants on chromatin. rxPAF15^K18RK27R^ failed to bind to chromatin as described above (Fig. 3b, see also Fig. 1d). Although rxPAF15^K18R^ or rxPAF15^K27R^ mutants bound to xPCNA on the chromatin as effectively as had rxPAF15^WT^, they failed to bind to xDNMT1(Fig. 3b). These results suggest that the binding of xPAF15 to xDNMT1 requires dual mono-ubiquitylation of PAF15 and that single mono-ubiquitylation of xPAF15 is not sufficient for the complex formation. This may also explain the apparently strong chromatin interaction of single mono-ubiquitylation of PAF15 K18R or K27R compared to dual mono-ubiquitylation of PAF15^WT^, likely due to defective recruitment of DNMT1/USP7 complex.

To further analyze the interaction between the RFTS of human DNMT1 (hRFTS) and PAF15Ub2, we prepared ubiquitylated hPAF15 (residues 2–30) analogs, in which G76C Ub was linked to K15C and/or K24C of hPAF15 by disulfide bonds (hPAF15_2-30_Ub2, hPAF15K15ub, and hPAF15K24Ub, Supplementary Fig. 3c, see “Methods”). The ITC experiment using hRFTS and hPAF15_2-30_Ub2 was performed under a condition with higher *c* value (*c* = *n*[titrand]/*K*_d_: 10,000) than that with an optimal value (1 < *c* < 1000) because the measurement using lower concentrations of proteins (even 1/4 of the original) resulted in an insufficient calorimetrical reaction for the reliable detection. Nevertheless, the results indicated that hRFTS binds to the hPAF15_2-30_Ub2 with high affinity (*K*_d_ = 1.4 ± 0.7 nM) in a 1:1 stoichiometric complex, which is comparable to that of hRFTS bound to H3Ub2^23^. In contrast, the binding affinity of hRFTS to hPAF15_2-30_K15Ub was much lower (*K*_d_ = 1.2 ± 0.8 μM) than that of hRFTS to hPAF15Ub2. Interaction of hRFTS with hPAF15_2-30_K24Ub resulted in a complex thermodynamic curve showing both exothermic and endothermic responses, which makes it difficult to determine its precise binding constant (*K*_d_ = n.d.). In addition, stoichiometric binding of 1:1 was abrogated in hRFTS:PAF15Ub1 at K15 or K24. These results indicate that dual mono-ubiquitylation of PAF15 is important for specific interaction with hRFTS. We then performed size-exclusion chromatography in line with small-angle X-ray scattering (SEC-SAXS) of hRFTS, hRFTS-hPAF15_2-30_Ub2, or hRFTS-H3_1-37W_Ub2 (dual mono-ubiquitylated at K18 and K23; Supplementary Table 1, see “Methods”). The molecular weight estimation based on *I*(0)/*c* (*c*: the concentration of protein) of Ovalbumin as a standard confirmed that the hRFTS-hPAF15_2-30_Ub2 or hRFTS-H3_1-37W_Ub2 formed the complex structure (Supplementary Fig. 3d, e and Supplementary Table 1). SAXS demonstrated that the radius of gyration (*R*_g_), the shape of distance distribution function *P*(*r*), and the maximum dimension *D*_max_ of hRFTS-hPAF15_2-30_Ub2 were almost identical to those of hRFTS-H3_1-37W_Ub2 (Supplementary Fig. 3f). We then confirmed whether xDNMT1 binds to xPAF15Ub2 with a binding mode similar to that used for binding xH3Ub2. Although rxDNMT1^WT^ bound to xPAF15Ub2 in denatured UbVS/Ub-treated chromatin lysates, xDNMT1 mutants harboring substitutions of amino acids within the RFTS essential for xH3Ub2 binding (P253AL256A or I317AI362A)^23^ failed to do so (Fig. 3d), indicating that the interaction of xDNMT1 with xPAF15Ub2 requires two mono-ubiquitin molecules that are conjugated on PAF15.

### xPAF15Ub2 is predominantly utilized for xDNMT1 recruitment

**Fig. 4:**
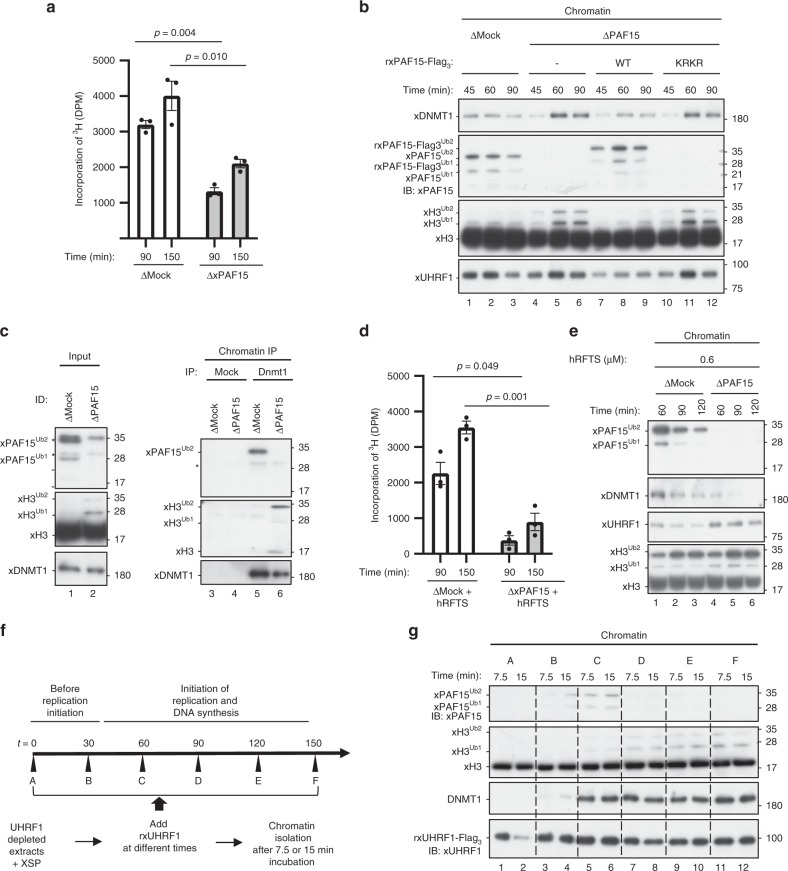
xPAF15Ub2 promotes recruitment of xDNMT1 and maintenance of DNA methylation. **a**, **d** Sperm chromatin was added to either mock- or xPAF15-depleted extracts containing radiolabeled S-[methyl-^3^H]-adenosyl-L-methionine in the absence (**a**) or presence of 0.6 μM hRFTS (**e**). The efficiency of DNA methylation was measured at the time points indicated. Bar graphs depict the quantification of incorporated SAM into genomic DNA with mean and SEM from three independent experiments. Statistical significance was determined using Student’s *t* test. **b**, **e** Sperm chromatin was added to mock- or xPAF15-depleted interphase extracts in the absence (**b**) or presence (**f**) of hRFTS. PAF15-depleted extracts were supplemented with either buffer alone (lanes 4–6), purified wild-type xPAF15-Flag_3_ or K18R/K27R(KRKR) mutant xPAF15-Flag_3_ (320 nM final concentration, lanes 7–9 or 10–12, respectively) in the experiment described in **b**. At the indicated time points, chromatin fractions were isolated and subjected to immunoblotting using the antibodies indicated. For the PAF15 levels in extracts, see Supplementary Fig. 2a. **c** Sperm chromatin was replicated in mock- or PAF15-depleted interphase egg extracts. Isolated and solubilized chromatin proteins were subjected to immunoprecipitation using an anti-xDNMT1 antibody. The resultant immunoprecipitates were analyzed by immunoblotting using the indicated antibodies. Asterisks, non-specifically detected proteins. **f** Schematic of experimental approach to test the differential regulation through UHRF1 during the progression of S phase. **g** Sperm chromatin was added to xUHRF1-depleted extracts and incubated for 0, 30, 60, 90, 120, or 150 min. Extracts were then supplemented with recombinant xUHRF1-Flag_3_ and further incubated for 7.5 or 15 min. Chromatin fractions were isolated and chromatin-bound proteins were analyzed by immunoblotting using the antibodies indicated. Source data are provided as a Source Data file.

We next examined the role of xPAF15Ub2 in the recruitment of xDNMT1 and subsequent maintenance of DNA methylation in *Xenopus* egg extracts. When xPAF15 was almost completely depleted from the extracts (Supplementary Fig. 4a), DNA replication-dependent DNA methylation of sperm DNA was partially suppressed compared to the control (Fig. 4a). Very importantly, although xH3Ub2 was hardly detected in the mock-depleted chromatin in clear contrast to xPAF15Ub2 (Fig. 4b, lanes 1–3), xH3Ub2 was drastically enhanced when xPAF15 was depleted (Fig. 4b, lanes 4–6). Addition of rxPAF15^WT^ to the endogenous xPAF15-depleted extracts suppressed the enhanced xH3Ub2, whereas that of rxPAF15^K18RK27R^ failed to do so (Fig. 4b, Supplementary Fig. 4b), suggesting that depletion of xPAF15 was complemented by xH3Ub2. Interestingly, the kinetics of xDNMT1 chromatin loading appeared to correlate with the dual mono-ubiquitylation of either xPAF15 or xH3. Consistent with this, DNMT1 predominantly interacted with PAF15Ub2, not with H3Ub2, on chromatin in mock-depleted extracts, whereas the level of H3Ub2 in the DNMT1 complex significantly increased in the absence of PAF15 (Fig. 4c). Taken together, our results reveal an essential role for PAF15Ub2 in maintenance of DNA methylation, which can only be partially compensated for by H3Ub2.

Complementation of xDNMT1 recruitment by xH3Ub2 in the PAF15-depleted extracts suggests that residual DNA methylation activity in these extracts originated from the enhanced xH3Ub2. To address this, we aimed to suppress the xH3Ub2-dependent pathway. As we previously observed that the deletion of the C-terminal region of DNMT1 largely increased the binding of the RFTS domain to unmodified H3 and H3Ub2^35^, it might be possible to preferentially suppress the xH3Ub2 pathway in extracts by supplying an optimal amount of recombinant hRFTS. We estimated that *Xenopus* interphase egg extracts contained ~0.1 μM of xDNMT1. Addition of 0.6 μM of hRFTS to the extracts resulted in the persistent presence of H3Ub2 on chromatin over the duration of S phase (Supplementary Fig. 4c), presumably due to suppression of xDNMT1 binding to xH3Ub2 and its deubiquitylation by xDNMT1-bound USP7^25^. Under these conditions, xPAF15Ub2 was also upregulated, but the effect appeared to be transient and much weaker than in the case of xH3Ub2 (Supplementary Fig. 4c). These results suggest that the addition of an optimal amount of RFTS selectively inhibited the xH3Ub2 pathway in terms of its ability to recruit xDNMT1(Supplementary Fig. 4c), apparently without affecting DNA replication and maintenance of DNA methylation. Importantly, the addition of 0.6 μM of hRFTS and the concomitant depletion of PAF15 dramatically reduced DNA methylation in the egg extracts (Fig. 4d). Consistent with this, chromatin loading of both xPAF15Ub2 and xDNMT1 was readily detectable in extracts in the presence of hRFTS, whereas that of xDNMT1 was not when xPAF15 was depleted (Fig. 4e). Taken together, our results indicate that UHRF1 primarily ubiquitylates PAF15 during S phase, promoting DNMT1 recruitment and subsequent maintenance of DNA methylation in *Xenopus* egg extracts.

We next addressed whether UHRF1 differentially ubiquitylates PAF15 and histone H3 during S-phase progression. To this end, we depleted endogenous xUHRF1 from interphase extracts and supplemented the reaction mixture with rxUHRF1^WT^ at various time points after the addition of sperm chromatin. Subsequently, chromatin fractions were isolated at the indicated time points, and the ubiquitylation of both PAF15 and H3 on the chromatin was assessed (Fig. 4f). rxUHRF1^WT^ added to UHRF1-depleted extracts before the start of DNA replication (*t* = 0 min, Supplementary Fig. 4d, e) did not effectively ubiquitylate PAF15 or histone H3 (Fig. 4g, lanes 1–2). In contrast, when rxUHRF1 was added back in early S phase (*t* = 30 or 60 min, Fig. 4g, lanes 3–6, Supplementary Fig. 4d, e), it restored PAF15 ubiquitylation and chromatin recruitment. However, the addition of rxUHRF1^WT^ at later time points (*t* = 90–150 min, lanes 7–12, Supplementary Fig. 4d, e) failed to restore PAF15 ubiquitylation and instead induced significant histone H3 ubiquitylation. Notably, unlike rUHRF1^WT^, the addition of rxUHRF1^D333A/D336A^ failed to induce histone H3 ubiquitylation under these conditions (Supplementary Fig. 4f). Consistent with the recruitment of DNMT1 via both PAF15 and histone H3 ubiquitylation, we found that DNMT1 loading was restored by the addition of rUHRF1 in both early and late S phase (Fig. 4g). UHRF1 therefore efficiently promotes PAF15 ubiquitylation during early S phase but prefers histone H3 as its substrate in late S phase for DNMT1 chromatin recruitment.

### PAF15 is important for maintenance of DNA methylation in mouse embryonic stem cells (mESCs)

**Fig. 5:**
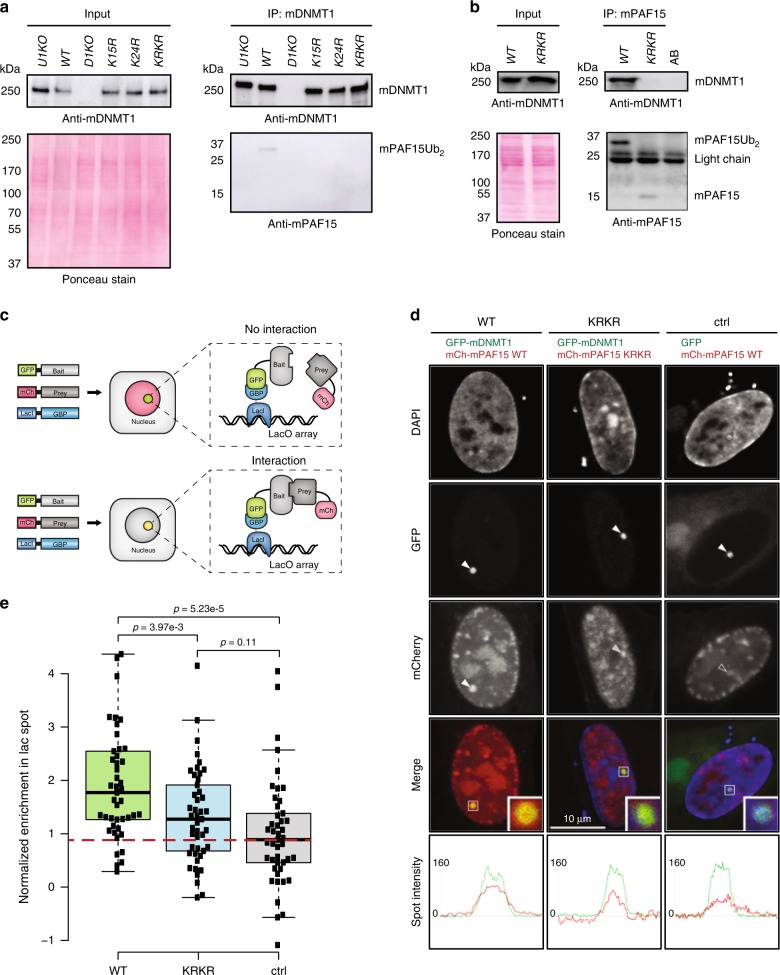
Dual mono-ubiquitylation of mPAF15 is required for the mPAF15–mDNMT1 interaction in mouse ESCs. **a** Immunoprecipitation of endogenous DNMT1 from whole-cell lysates of wild-type J1 (WT), *Dnmt1* KO (D1KO), *Uhrf1* KO (U1KO), *Paf15 *K15R (K15R), *Paf15 *K24R (K24R), and *Paf15 *K15/24R (KRKR) mESCs using an anti-mDNMT1 nanobody. Bound fractions were subjected to immunoblotting with anti-mDNMT1 and anti-mPAF15 antibodies. The anti-mDNMT1 blot and Ponceau staining are shown as loading controls. **b** Immunoprecipitation of endogenous mPAF15 from WT and KRKR mESC nuclear extracts using an anti-mPAF15 antibody. Bound fractions were subjected to immunoblotting with anti-mDNMT1 and anti-mPAF15 antibodies. The anti-mDNMT1 blot and Ponceau staining are shown as loading controls. **c** Schematic of the fluorescent-3-hybrid (F3H) assay for the in vivo determination of protein–protein interactions. GFP-tagged bait protein is immobilized at an array of Lac operator (LacO) sequences by a GFP-binding protein (GBP) coupled to the lac repressor (LacI). When the GFP-tagged bait protein does not interact with the prey protein, only a GFP signal is visible at the LacO locus, whereas a yellow spot (combination of GFP and mCherry signal) is visible at the LacO locus in the case of a positive interaction. **d**, **e** F3H assay for a BHK cell-based analysis of ubiquitylation-mediated recruitment of mPAF15 to mDNMT1. **d** Cells containing a stably integrated lacO array were transfected with the GBP-LacI, a GFP-tagged bait (GFP-mDNMT1 or GFP), and an mCherry-tagged prey (mCherry-mPAF15 wild-type (WT) or mCherry-mPAF15 K15R/K24R double mutant (KRKR)). Line intensity profiles for GFP and mCherry in the respective spots are shown below the confocal images. Scale bar, 10 μm. **e** Quantification of the F3H assay. Background subtracted mCherry/GFP ratios within the spots were normalized to the control and plotted with *n* = 45 from 3 independent replicates (per replicate, *n* = 15). In the boxplots, horizontal black lines within boxes represent median values, boxes indicate the upper and lower quartiles, and whiskers indicate the 1.5× interquartile range. Statistical significance was determined using Student’s *t* test. Source data are provided as a Source Data file.

We had previously shown that murine UHRF1 (mUHRF1) ubiquitylates two neighboring lysines at the N-terminus of mPAF15 (K15 and K24)^36^ with a similar spacing as in histone H3, suggesting a similar role in the recruitment of DNMT1 and the maintenance of DNA methylation in murine cells. To investigate the interaction between murine DNMT1 (mDNMT1) and mPAF15 and the role of ubiquitylation, we used CRISPR/Cas9-based gene editing to introduce K15R, K24R, or both K15R/K24R (KRKR) mutations into the endogenous *Paf15* gene in mESCs (Supplementary Fig. 5a–g). Co-IP experiments with antibodies against mDNMT1 yielded a faint band of ~34 kDa corresponding to mPAF15Ub2 in WT ESCs, but no co-precipitation was detected with mPAF15 lacking either a single (K15R or K24R) or both ubiquitylation sites (KRKR) or in cells without mUHRF1 (U1KO) (Fig. 5a). As we could detect mPAF15 only in precipitates but not in the less concentrated input controls, we performed the reciprocal experiment. Upon IP with antibodies against mPAF15, we detected mPAF15Ub2 co-precipitating mDNMT1 in WT ESCs (Fig. 5b). In the KRKR mutant line, however, we detected a weaker band at ~15 kDa corresponding to the unmodified mPAF15, which did not co-precipitate mDNMT1 (Fig. 5b). The weaker signal obtained for mPAF15 KRKR was mostly due to losses during nuclear extract preparation as half of the unmodified mPAF15 was in the cytosol while the ubiquitylated mPAF15Ub2 was bound in the nucleus (Supplementary Fig. 6a). This diffuse distribution of the unmodified mPAF15 may in part be caused by reduced interactions with nuclear proteins. To be able to better compare the interaction and precipitation efficiency of WT mPAF15 and mutant mPAF15 KRKR, we titrated precipitates to comparable levels and could show that the modified WT mPAF15Ub2 clearly binds and precipitates mDNMT1 more efficiently (Supplementary Fig. 6b).

To validate the above findings with an independent approach, we applied a fluorescent-3-hybrid (F3H) assay^37^ to assess the interaction of mDNMT1 and mPAF15 in vivo. In brief, the F3H assay provides a means of quantifying protein–protein interactions in living cells by measuring the efficiency with which a green fluorescent protein (GFP)-tagged “bait” protein immobilized at a nuclear spot (lacO array) is able to recruit a different, fluorescently labeled “prey” protein (schematic in Fig. 5c). For the negative control, cells were transfected with monomeric GFP and mCherry-tagged WT mPAF15. As expected, GFP was effectively immobilized at the lacO spot yet failed to efficiently recruit mCherry-tagged WT PAF15 (Fig. 5d, e). We next co-expressed GFP-mDNMT1 in addition to either mCherry-tagged WT or the ubiquitylation-deficient mPAF15 (KRKR) harboring both K15R and K24R substitutions. In contrast to monomeric GFP, immobilized GFP-mDNMT1 readily recruited a significant fraction of WT mPAF15 to the lacO spot, but not mPAF15-KRKR (Fig. 5d, e). These results demonstrate that mammalian DNMT1 and PAF15 interact in vivo and show this interaction to be dependent, as in *Xenopus* (Fig. 3b), on dual mono-ubiquitylation of PAF15.

We next sought to determine whether the endogenous interaction of mDNMT1 and mPAF15Ub2 serves a role in the maintenance of DNA methylation in mESCs. To this end, we first used high-content immunofluorescence-based detection of 5-methylcytosine (5-mC) to measure DNA methylation levels in our *Paf15* mutant ESC lines as well as in control cell lines, *Uhrf1* knockout (*Uhrf1* KO) and *Dnmt1* knockout (*Dnmt1* KO) ESCs, lacking maintenance of DNA methylation activity (Supplementary Fig. 6c). As expected, *Dnmt1* KO and *Uhrf1* KO ESCs exhibited a near complete loss of DNA methylation (Supplementary Fig. 6c). Strikingly, *Paf15* single (K15R and K24R) and double mutant (KRKR) ESCs also displayed a substantial global reduction in DNA methylation when compared with WT ESCs (Supplementary Fig. 6c). However, global DNA methylation levels in *Uhrf1* KO ESCs, in which both mH3Ub2 and mPAF15Ub2 are absent, were lower than those in *Paf15* mutant ESCs, suggesting that the H3Ub2 pathway can partially compensate for the loss of mPAF15Ub2 (Supplementary Fig. 6c). Taken together, these results revealed that mPAF15Ub2 has an essential and largely non-redundant role in ensuring proper maintenance of DNA methylation in mESCs.

**Fig. 6:**
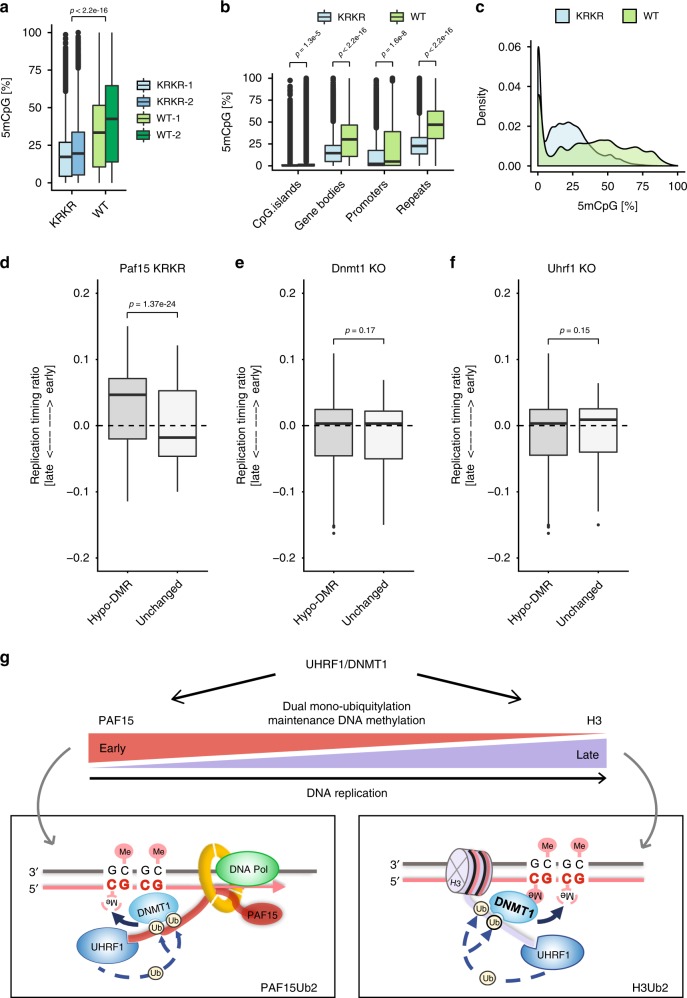
mPAF15Ub2 is required for the proper maintenance of DNA methylation in mouse ESCs. **a**, **b** DNA methylation levels (%) as measured by RRBS in wild-type (WT) and *Paf15* K15R/K24R (KRKR) double mutant ESCs. **a** Global DNA methylation levels and **b** CpG methylation levels at CpG islands, promoters, gene bodies, and repeats in wt and KRKR ESCs. *p* Values based on ANOVA with post hoc Tukey’s test. **c** Density plot depicting the distribution of DNA methylation levels of individual CpG sites in wt and KRKR ESCs. **d**–**f** Replication timing of hypomethylated vs. unchanged tiles in **d** *Paf15* KRKR ESCs, **e** *Dnmt1* KO ESCs, and **f** *Uhrf1* KO ESCs. For comparisons between hypomethylated and unchanged tiles, Welch’s two-sided *t* test was used for calculating *p* values. Differentially methylated tiles losing DNA methylation (hypomethylated tiles) were defined as those with *p* < 0.05 and a methylation loss >25%; *p* values were derived from a methylKit package (see “Methods”). **g** Model of the two pathways of dual mono-ubiquitylation facilitating maintenance of DNA methylation. Both requiring UHRF1, PAF15Ub2 and H3Ub2 preferentially contribute to the DNMT1-mediated maintenance of DNA methylation of early and late replicating regions, respectively. For the boxplots in **a**, **b**, **d**–**f**, the horizontal black lines within boxes represent median values, boxes indicate the upper and lower quartiles, and whiskers indicate the 1.5× interquartile range. Source data are provided as a Source Data file.

To assess how mPAF15Ub2 shapes the methylome of mESCs at single-nucleotide resolution, we performed reduced representation bisulfite sequencing (RRBS) on WT and *Paf15* KRKR mESCs (Supplementary Table 2). Consistent with our immunofluorescence measurements, RRBS analysis revealed a significant loss of global DNA methylation in *Paf15* KRKR ESCs compared to WT ESCs (Fig. 6a, c, Supplementary Fig. 6d). Furthermore, we observed a significant decrease in DNA methylation levels at all genomic regions examined, including repetitive elements, gene bodies, promoters, and CpG islands in *Paf15* KRKR mESCs (Fig. 6b). To determine whether mPAF15Ub2-dependent methylation is associated with particular chromatin features, we analyzed the levels of several histone modifications (H3K9me2^38^, H3K9me3^39^, and H3K14ac^40^) at regions differentially methylated in *Paf15* KRKR ESCs (*p* < 0.05 and methylation difference >25%). However, we found neither active nor repressive histone modifications to be enriched at hypomethylated regions resulting from mPAF15Ub2 loss (Supplementary Fig. 6f). We then analyzed how the loss of mPAF15Ub2 affects DNA methylation levels of lamina-associated, late-replicating regions found to be hypomethylated in a multitude of cancer types^41^. These hypomethylated regions, referred to as partially methylated domains (PMDs), differ from the heavily methylated domains (HMDs) comprising the bulk of the remaining genome^42^. Our RRBS analysis demonstrated a stark reduction in DNA methylation at both PMDs and HMDs in *Paf15* KRKR ESCs (Supplementary Fig. 6e), suggesting that mPAF15Ub2 contributes to the maintenance of DNA methylation at both PMDs and HMDs in mESCs.

To investigate the relationship between replication timing and mPAF15Ub2-dependent maintenance of methylation, we compared our *Paf15* KRKR methylome data with Repli-seq maps from ESCs^43^. Remarkably, regions hypomethylated in *Paf15* KRKR ESCs were associated with a significantly earlier replication timing than regions of unchanged DNA methylation, which on average tended to replicate later in S phase (Fig. 6d). These results indicate that mPAF15Ub2 has an essential role in the maintenance of DNA methylation, especially at early replicating sequences, and imply that mH3Ub2 is sufficient to sustain DNA methylation at late replicating regions in the absence of mPAF15Ub2. In contrast to Paf15 KRKR ESCs, the average replication timing of hypomethylated regions in *Uhrf1* KO and *Dnmt1* KO ESCs was essentially identical to that of regions of unchanged DNA methylation (Fig. 6e, f). These results indicate that the complete disruption of maintenance of DNA methylation by genetic ablation of DNMT1 or UHRF1, which abolishes both mPAF15Ub2 and mH3Ub2, leads to genome-wide hypomethylation irrespective of replication timing. Together, these data show that mPAF15Ub2 and mH3Ub2 constitute two distinct pathways of mDNMT1 recruitment that together accomplish complete maintenance of DNA methylation throughout every cell cycle (Fig. 6g).

## Discussion

Our current study provides clear evidence that PAF15 within DNA replication machinery complexes plays a pivotal role in the maintenance of DNA methylation. We have recently reported that UHRF1-mediated H3Ub2 recruits DNMT1 to DNA methylation sites, which likely functions independently of DNA replication fork progression. PAF15 in a complex with PCNA also undergoes UHRF1-mediated dual mono-ubiquitylation, which is essential for DNMT1 recruitment, and subsequent maintenance of DNA methylation. Thus, our results suggest that dual mono-ubiquitylation at two lysine residues spaced by 4–9 amino acids (mH3 K14~K18~K23 and mPAF15 K15~K24) in the flexible region of the proteins serves as a specific code for the maintenance of DNA methylation. This notion is supported by the finding that the recognition of PAF15Ub2 by RFTS was very similar to that of H3Ub2.

The fact that UHRF1 targets two distinct proteins, histone H3 and PAF15, for generating a specific code is consistent with the previous report that there are two modes of maintenance of DNA methylation^29^. As to why UHRF1 would have two modes of usage, our results strongly suggest that PAF15Ub2 and H3Ub2 function in different contexts depending on the replication timing, as PAF15 ubiquitylation occurs only during early S phase, whereas histone H3 ubiquitylation can be induced in late S phase. It has been reported that DNA methylation levels are different between early and late replicating domains, with the former containing a much higher degree of DNA methylation than the latter^44^. The enrichment of DNA methylation sites in early replicating domains would explain why cells expressing mPAF15-K15R/K24R have a substantial loss of DNA methylation as observed in mESCs, as well as in *Xenopus* egg extracts. Although H3Ub2 is markedly increased when PAF15Ub2 is perturbed, it might occur with less efficiency in early replicating domains.

H3Ub2 might also play a dominant role in the recruitment of DNMT1 under particular conditions in which PAF15 is not functional. For example, the replication block induced by ultraviolet (UV) irradiation leads to PAF15 poly-ubiquitylation and subsequent proteasomal degradation^33^. Therefore, replication fork stalling across heterochromatin at late replicating domains might induce PAF15 degradation^45^, which might then be compensated for by H3 ubiquitylation. Alternatively, H3 ubiquitylation could function as a proofreader for the failure of DNA methylation by PAF15Ub2-dependent DNMT1 recruitment. Consistent with this idea, the level of xH3Ub2 on chromatin as well as in complex with xDNMT1 increased upon xPAF15 depletion and the masking of H3Ub2 by RFTS in the absence of PAF15 resulted in an almost complete loss of DNA methylation. Whereas deletion of *Dnmt1* or *Uhrf1* causes embryonic lethality^7,8,46^, it is noteworthy that *Paf15* knockout mice remain viable despite abnormal hematopoietic stem cell function^47^. These observations suggest that loss of PAF15 function in the recruitment of DNMT1 could partly be compensated for by histone H3, ensuring the stable inheritance of DNA methylation.

We found that both the interaction with PCNA and dual mono-ubiquitylation by UHRF1 are essential for PAF15 function in the maintenance of DNA methylation. PAF15 is an intrinsically disordered protein and binds to trimeric PCNA via the PIP-box motif at the front face and its N-terminus interacts with the inner ring of PCNA and exits the clamp from the back face^48^, suggesting that the ubiquitylation sites of PAF15 could locate near the nascent strand where a methyl group does not yet exist. Thus, PAF15Ub2 could directly recruit DNMT1 to the back face of PCNA, facilitating the processivity of DNMT1-mediated DNA methylation on the nascent DNA (Fig. 6g). These structural features are also consistent with the fact that early replicating domains contain a much higher degree of DNA methylation at which time PAF15Ub2 is predominantly recruiting DNMT1. We also note that, during the revision of our manuscript, a recent study has also shown that full-length hPAF15Ub2 binds DNMT1 in vitro^49^.

In conclusion, we propose that maintenance of DNA methylation is coordinated with S-phase progression via UHRF1-dependent dual mono-ubiquitylation of two distinct proteins, PAF15 and histone H3, which may contribute to the robustness of DNA maintenance methylation by ensuring the recruitment and activation of DNMT1 (Fig. 6g). Further research is required to clarify how the different modes of DNMT1 recruitment are chosen and to identify potential additional factors contributing to the dual mono-ubiquitylation signaling of DNA maintenance methylation.

## Methods

### Primers

All oligonucleotide sequences are listed in Supplementary Table 3.

### *Xenopus* egg extracts

*Xenopus laevis* was purchased from Kato-S Kagaku and handled according to the animal care regulations at the University of Tokyo. Preparation of interphase egg extracts, chromatin isolations, immunodepletions, and UbVS reactions was performed as described previously^23^ with minor modifications. Briefly, all extracts were supplemented with energy regeneration mix (2 mM ATP, 20 mM phosphocreatine, and 5 μg/ml creatine kinase). Demembranated sperm nuclei (3000–4000 sperm/μl in the final reaction) were added to egg extracts and incubated at 22 °C. For chromatin spin-down from the egg extracts, sperm nuclei were incubated in 15–25 μl of the extract preparation. The extracts were diluted with ten volumes of ice-cold chromatin purification buffer (CPB; 50 mM KCl, 5 mM MgCl_2_, 20 mM HEPES-KOH, pH 7.7) containing 2% sucrose, 0.1% NP-40, and 2 mM *N*-ethylenemaleimide (NEM) and kept on ice for 5 min. Diluted extracts were underlayed with 1.5 ml of a 30% sucrose cushion in CPB and centrifuged at 15,000 × *g* for 10 min at 4 °C using a swing–bucket rotor. The pellets were resuspended in Laemmli sample buffer. For xPAF15 depletion, 250 μl of antiserum were coupled to 50 μl of recombinant protein A-sepharose (rPAS, GE Healthcare). Antibody beads were washed three times in phosphate-buffered saline (PBS) and added with 5 μl fresh rPAS. Beads were washed twice in CPB, split into three portions, and 100 μl extracts were depleted in three rounds at 4 °C, each for 1 h. For xUHRF1 depletion, 170 μl of antiserum were coupled to 35 μl of rPAS. Antibody beads were washed three times in PBS and added with 4 μl fresh rPAS. Beads were washed twice in CPB, split into two portions, and 100 μl extracts were depleted in two rounds at 4 °C, each for 1 h. For xDNMT1 depletion, 250 μl of antiserum were coupled to 50 μl of rPAS. Antibody beads were washed three times in PBS and added with 5 μl fresh rPAS. Beads were washed twice in CPB, split into three portions, and 100 μl extracts were depleted in three rounds at 4 °C, each for 1 h. For add-back experiments, recombinant xPAF15 was added to xPAF15-depleted extracts at 320 nM, recombinant xUHRF1 was added to xUHRF1-depleted extracts at 110 nM, and recombinant xDNMT1 was added to xDNMT1-depleted extracts at 85 nM.

For UbVS reactions, egg extracts were incubated with 20 μM UbVS (Boston Biochem, Cambridge, MA, USA) for 30 min at 22 °C. Sperm nuclei were then added to egg extracts with or without 58 μM ubiquitin (Boston Biochem). For quantification of PAF15Ub2 on chromatin, immunoblot films from three independent experiments were scanned. The pixel intensity of protein bands was then quantified with Image J, and the average intensity normalized to UHRF1 was calculated for each set of conditions. Antibodies against xPAF15 were raised in rabbits by immunization with a GST-tagged recombinant full-length xPAF15. Antisera were further affinity-purified with the recombinant protein immobilized on a nitrocellulose membrane (1:500 dilution for western blots). Rabbit polyclonal antibodies raised against *Xenopus* DNMT1 and UHRF1 have been previously described^17^ (1:500 dilution for western blots). Rabbit polyclonal USP7 antibody A300-033A was purchased from Bethyl Laboratories (1:1000 dilution for western blots). Mouse monoclonal antibody against PCNA PC-10 was purchased from Santa Crutz Biotechnology and used for immunoblotting (1:1000 dilution for western blots). Rabbit polyclonal histone H3 antibody ab1791 was purchased from Abcam (1:3000 dilution for western blots). The following antibodies were generous gifts: xPCNA antibody used for IPs (TS. Takahashi, Kyusyu Univ.), xORC2 (J. Maller, University of Colorado, 1:1000 dilution for western blots), and xCdt1 (Marcel Mechali, CNRS, 1/2000 dilution for western blots). For IP, 10 μl of Protein A agarose (GE Healthcare) was coupled with 2 μg of purified antibodies or 5 μl of antiserum. The agarose beads were washed twice with CPB buffer containing 2% sucrose. The antibody beads were incubated with egg extracts for 1 h at 4 °C. The beads were washed four times with CPB buffer containing 2% sucrose and 0.1% Triton X-100 and resuspended in 20 μl of 2× Laemmli sample buffer.

### Pull-down of DNMT1-interacting proteins from chromatin

MNase-digested chromatin fractions were prepared as described previously^23,35^. Briefly, the chromatin pellet was resuspended and digested in 100 μl of digestion buffer (10 mM HEPES-KOH, 50 mM KCl, 2.5 mM MgCl_2_, 0.1 mM CaCl_2_, 0.1% Triton X-100, pH 7.5, and 10 μM PR-619) containing 4 U/ml micrococcal nuclease (MNase) at 22 °C for 20 min. The reaction was stopped by the addition of 10 mM EDTA, and the mixture was centrifuged at 17,700 × *g* for 10 min. To prepare denatured chromatin lysates, the supernatant was treated with 1% SDS and then immediately diluted with lysis buffer (150 mM NaCl, 1% Triton X-100, 1 mM EDTA, 15 mM Tris-HCl, pH 8.0). For the pull-down experiment, 5 μg purified recombinant xDNMT1 or its mutant were coupled with 20 μl of anti-FLAG M2 affinity resin at 4 °C for 1 h. The beads were collected and washed with CPB buffer containing 2% sucrose and then incubated with MNase-digested chromatin. After incubation at 4 °C for 1 h, beads were washed with CPB buffer containing 0.1% Triton X-100. Bound proteins were analyzed by immunoblotting.

### Mass spectrometry

Liquid chromatography–tandem mass spectrometry (LC-MS/MS) analyses were performed essentially as previously described^50^ with some modifications. Immunoprecipitated proteins were separated by SDS-polyacrylamide gel electrophoresis (PAGE) and stained with Bio-Safe Coomassie (Bio-Rad). Gels were washed in Milli-Q water, and excised. The gel pieces were washed in 50 mM ammonium bicarbonate (AMBC)/30% acetonitrile (ACN) for >2 h, and subsequently with 50 mM AMBC/50% ACN for >1 h. The gels were then dehydrated in 100% ACN for 15 min. Trypsin digestion was performed by incubation at 37 °C for 12–15 h with 20 ng/μl modified sequence-grade trypsin (Promega) in 50 mM AMBC and 5% ACN, pH 8.0. After digestion, the peptides were extracted four times with 0.1% trifluoroacetic acid/70% ACN. Extracted peptides were concentrated by vacuum centrifugation. For the LC-MS/MS analyses, a Nanoflow UHPLC, Easy nLC 1000 (Thermo Fisher Scientific) was connected online to a quadrupole-equipped Orbitrap MS instrument (Q Exactive, Thermo Fisher Scientific) with a nanoelectrospray ion source (Thermo Fisher Scientific). Peptides were separated on C18 analytical columns (Reprosil-Pur 3 μm, 75 μm id × 12 cm packed tip column, Nikyo Technos Co., Ltd.) in a 90-min three-step gradient (0–40% Solvent B for 72 min, 40–100% for 12 min, and 100% for 6 min) at a constant flow rate of 300 nl/min. The Q Exactive was operated using the Xcalibur software (Thermo Fisher Scientific) in data-dependent MS/MS mode, and the top 10 most intense ions with a charge state of +2 to +5 were selected with an isolation window of 2.0 *m*/*z* and fragmented by higher-energy collisional dissociation with a normalized collision energy of 28. Resolution and automatic gain control targets were set to 70,000 and 3E6, respectively. The data were analyzed using the Sequest HT search program in Proteome Discoverer 2.2 (Thermo Fisher Scientific). The maximum of missed cleavage sites of trypsin was set to three. Acetylation (Protein N-term and Lys), oxidation (Met), GlyGly modification (Lys), phosphorylation (Ser, Thr, Tyr), and pyroglutamate conversion (N-term Gln) were selected as variable modifications. Peptide identification was filtered at a false discovery rate <0.01. Non-label protein quantification was performed using the Precursor Ions Quantifier node in Proteome Discoverer 2.2. The RAW files have been deposited to the ProteomeXchange Consortium^51,52^.

### Recombinant *Xenopus* proteins

The *X. laevis* *Paf15* cDNA was amplified by PCR from a *X. laevis* cDNA library using primers 3621 and 3622 and ligated into pTA2 vector. For GST-xPAF15 expression, the amplified *xPaf15* genes with primers 3667 and 3636 were gel-isolated and ligated into linearized pGEX4T-3 using In-Fusion (Clontech) according to the manufacturer’s instructions. Protein expression in *Escherichia coli* (BL21-CodonPlus) was induced by the addition of 0.1 mM isopropyl β-d-1-thiogalactopyranoside to media followed by incubation for 12 h at 20 °C. For purification of GST-tagged proteins, cells were collected and resuspended in lysis buffer (20 mM HEPES-KOH pH 7.6, 0.5 M NaCl, 0.5 mM EDTA, 10% glycerol, 1 mM dithiothreitol (DTT)) supplemented with 0.5% NP40 and protease inhibitors and were then disrupted by sonication on ice. After centrifugation, the supernatant was applied to glutathione Sepharose beads (GE Healthcare) and rotated at 4 °C for 2 h. Beads were then washed three times with wash buffer 1 (20 mM Tris-HCl pH 8.0, 150 mM NaCl, 1% TritionX-100, 1 mM DTT) and once with wash buffer 2 (100 mM Tris-HCl (pH 7.5), 100 mM NaCl). Bound proteins were released in elution buffer (100 mM Tris-HCl pH 7.5, 100 mM NaCl, 5% glycerol, 1 mM DTT) containing 42 mM reduced glutathione, and the purified protein was loaded on a PD10 desalting column equilibrated with EB buffer (10 mM HEPES/KOH at pH 7.7, 100 mM KCl, 0.1 mM CaCl_2_, 1 mM MgCl_2_) containing 1 mM DTT and then concentrated with Vivaspin (Millipore).

For protein expression in insect cells, C-terminally 3× Flag-tagged *xPaf15* genes were transferred from pKS104 vector into pVL1392 vector. The amplified *xPaf15* genes with primers 3720 and 3581 were gel-isolated and ligated into linearized pVL1392 using In-Fusion. R3A, T4D, K5A, K18R, K27R, K18R/K27R, and F72AF73A mutations in pKS104-xPAF15 or pVL1392-xPAF15 constructs were introduced using a KOD-Plus Mutagenesis Kit (Toyobo). All mutations were confirmed by Sanger sequencing. Baculoviruses were produced using a BD BaculoGold Transfection Kit and a BestBac Transfection Kit (BD Biosciences), following the manufacturer’s protocol. Proteins were expressed in Sf9 insect cells by infection with viruses expressing xPAF15 WT-3× Flag or its mutant for 72 h. Sf9 cells from a 500 ml culture were collected and lysed by resuspending them in 20 ml lysis buffer (20 mM Tris-HCl, pH 8.0, 100 mM KCl, 5 mM MgCl_2_, 10% glycerol, 1% Nonidet P40 (NP-40), 1 mM DTT, 10 μg/ml leupeptin, and 10 μg/ml aprotinin), followed by incubation on ice for 10 min. A soluble fraction was obtained after centrifugation of the lysate at 15,000 × *g* for 15 min at 4 °C. The soluble fraction was incubated for 4 h at 4 °C with 250 μl of anti-FLAG M2 affinity resin (Sigma-Aldrich) equilibrated with lysis buffer. The beads were collected and washed with 10 ml wash buffer (20 mM Tris-HCl, pH 8.0, 100 mM KCl, 5 mM MgCl_2_, 10% glycerol, 0.1% NP-40, 1 mM DTT) and then with 5 ml EB (20 mM HEPES-KOH, pH 7.5, 100 mM KCl, 5 mM MgCl_2_) containing 1 mM DTT. The recombinant xPAF15 was eluted twice in 250 μl EB containing 1 mM DTT and 250 μg/ml 3× FLAG peptide (Sigma-Aldrich). Eluates were pooled and concentrated using a Vivaspin 500 (GE Healthcare Biosciences). We note that all PAF15 mutant proteins was purified as efficiently as the WT protein from insect cells. For expression of C-terminally 3× Flag-tagged xUHRF1 (Wt and D333A/D336A), C-terminally 3× Flag-tagged *xUhrf1* genes were transferred from pKS104 vector into pVL1392 vector. The amplified *xUHRF1* genes with primers 4620 and 3581 were gel-isolated and ligated into linearized pVL1392 using In-Fusion. The D333A/D336A substitution was introduced using a KOD-Plus Mutagenesis Kit and confirmed by Sanger sequencing. Recombinant xUHRF1 proteins were also purified as described above.

### DNA methylation and replication in *Xenopus* egg extracts

DNA methylation was monitored by the incorporation of S-[methyl-^3^H]-adenosyl-L-methionine, incubated at room temperature, and the reaction was stopped by the addition of CPB containing 2% sucrose up to 300 μl. Genomic DNA was purified using a Wizard Genomic DNA Purification Kit (Promega) according to the manufacturer’s instructions. Incorporation of radioactivity was quantified with a liquid scintillation counter. DNA replication was assayed by adding [α-^32^P]-dCTP to egg extracts containing sperm chromatin. The reaction was stopped by adding 1% SDS, 40 mM EDTA and spotted onto Whatman glass microfiber filters followed by trichloroacetic acid (TCA) precipitation with 5% TCA containing 2% pyrophosphate. Filters were washed in ethanol, dried, and TCA-precipitated radioactivity was counted in scintillation liquid.

### Structure of the UHRF1 PHD finger bound to PAF15_2-11_

The PHD finger of human UHRF1 (residues 299–366) was expressed as a fusion protein with GST and small ubiquitin-like modifier-1 (SUMO-1) at its N-terminus. Cell culture and purification were performed according to our previous report^14,23^. Briefly, hPHD was purified using GST affinity column of glutathione Sepharose 4B (GS4B: GE Healthcare). The GST-SUMO-1 fused hPHD was eluted with reduced glutathione and then GST-SUMO-1 tag was removed by the SUMO-specific protease GST-SENP2. The protein was further purified by anion-exchange chromatography using a HiTrap Q HP column and by SEC using a HiLoad 26/60 Superdex75 column (GE Healthcare). PAF15_2-11_ was synthesized at Toray Research Center (Tokyo, Japan). The PHD finger:PAF15_2-11_ complex was prepared by adding a 1.5-molar excess of the PAF15_2-11_ peptide to the protein before its concentration using an Amicon concentrator with a 3000 Da cutoff (Millipore). The crystal was obtained using a 30 mg/ml concentration of the complex at 20 °C and the hanging drop vapor diffusion method with a reservoir solution containing 0.1 M HEPES-NaOH (pH 7.5) and 70% (v/v) 2-methyl-2,4-pentanediol. The crystal was directly frozen in liquid nitrogen. The X-ray diffraction data were collected at a wavelength of 0.98000 Å on a Pilatus3 6M detector in beam line BL-17A at Photon Factory (Tsukuba, Japan) and scaled at 1.70 Å resolution with the program XDS package^53^ and Aimless^54^. This was followed by molecular replacement by PHASER^55^ and several cycles of model refinement by PHENIX^56^. The final model converged at 1.70 Å resolution with a crystallographic *R*-factor of 17.6% and a free *R*-factor of 18.7%. The crystallographic data and refinement statistics are given in Table 1. The figures were generated using PyMOL (http://www.pymol.org).

### ITC measurements

Preparation and purification of the disulfide linked K15 and K24 mono-ubiquitylated analog of human PAF15_2-30_ and the human DNMT1 RFTS domain, residues 351–600, were performed according to our previous report^23^. hRFTS was purified using GST-affinity column of GS4B. After removing the GST-SUMO1 tag by GST-SENP2, the protein was further purified by anion-exchange chromatography of HiTrap Q HP column and HiLoad 26/60 Superdex75 column. hPAF15_2-30_ K15C/K24C mutant was synthesized at Toray Research Center (Tokyo, Japan). Ubiquitin G76C mutant activated by 5,5′-dithiobis-(2-nitrobenzoic acid) (Wako) and hPAF15_2-30_ K15C/K24C were incubated for 1 h at room temperature and then purified by cation-exchange chromatography of Mono-S (GE Healthcare) to separate from the by-products. The UHRF1 PHD finger was buffer-exchanged using Superdex 200 Increase 10/300 GL (GE Healthcare) equilibrated with 10 mM HEPES-NaOH (pH 7.5), 150 mM NaCl, and 0.25 mM tris(2-carboxyethyl)phosphine hydrochloride and lyophilized PAF15 (residues 2–11) peptide was dissolved in the same buffer. hRFTS and PAF15_2-30_Ub2 were equilibrated with 10 mM HEPES-NaOH (pH 7.5), 150 mM NaCl, and 10 μM zinc acetate. A MicroCal LLC calorimeter, VP-ITC (MicroCal), was used for the ITC measurements. The data were analyzed with the software ORIGIN (MicroCal) using a one-site model.

### In vitro ubiquitylation assay

Protein expression in *E. coli* and purification of mouse UBA1 (E1), human UHRF1 (WT and its mutants), and ubiquitin were performed according to the previous reports^17^. E1 enzyme was expressed in *E. coli* Rosetta 2 (DE3) (Novagen) as a six histidine-tag fusion protein. The protein was purified using TALON^®^ (Clontech), HiTrap Q HP, and SEC using HiLoad 26/60 Superdex200 column (GE Healthcare). hUHRF1 WT and mutants, D334A/D337A and H741A, were expressed in *E. coli* Rosetta 2 (DE3) as a GST-fusion protein. The protein was purified GST-affinity chromatography of GS4B column. After removal of GST-tag by HRV-3C protease, the protein was further purified by HiTrap Heaparin HP column (GE Healthcare) and HiLoad 26/60 Superdex200 column. Purification procedure of ubiquitin was as follows: after cell lysis and centrifugation, the supernatant was boiled at 85 °C for 15 min. After removing the debris by centrifugation, ubiquitin was further purified using cation-exchange chromatography of HiTrap SP HP (GE Healthcare) and HiLoad 26/60 Superdex75 column. UBCh5 (E2) was purified using TALON^®^ and SEC of HiLoad 26/60 Superdex75 column. The cDNA encoding amino acids 2–71 of human PAF15 harboring HA-tag at the C-terminus was cloned into the modified pET21b vector, pET-N^pro^ vector^57^. The N^pro^-fused PAF15 was purified from the pellet fraction. The inclusion body was then solubilized in buffer containing 8 M urea, 50 mM Tris-HCl (pH 7.5), and 25 mM DTT by stirring overnight at 4 °C. Then the denatured fusion proteins were purified by Ni Sepharose 6 Fast Flow (GE Healthcare). The eluents were dialyzed in a step-wise manner to gradually remove the urea. The solution was additionally incubated with a buffer containing 100 mM Tris-HCl (pH7.5), 200 mM NaCl, and 2 mM DTT for 12–24 h at room temperature for completing autocleavage of N^pro^. The protein was further purified using HiTrap SP HP and HiLoad 16/60 Superdex 30 (GE Healthcare).

Standard ubiquitylation reaction mixtures contained 116 μM ubiquitin, 200 nM E1, 6 μM E2, 3 μM E3, 5 mM ATP, and 50 μM PAF15-HA as a substrate in ubiquitylation reaction buffer (50 mM HEPES [pH 8.0], 150 mM NaCl, 5 mM MgCl_2_, 0.1% Triton X-100, 2 mM DTT). The mixture was incubated at 30 °C for 30 min, and the reaction was stopped by adding 3× SDS loading buffer. The reaction was analyzed by SDS-PAGE, followed by western blotting using 1/20,000 diluted anti-HA antibody (MBL, #M180-3).

### SEC-SAXS data collection, processing, and interpretation

SAXS data were collected on Photon Factory BL-10C using a UPLC^®^ ACQUITY (Waters) integrated SAXS set-up. Fifty μl of a 6 mg/ml sample were loaded onto a Superdex 200 Increase 5/150 GL (GE Healthcare Science) pre-equilibrated with 20 mM Tris-HCl (pH 8.0), 150 mM NaCl, and 5% glycerol at a flow rate of 0.25 ml/min at 4 °C. The flow rate was reduced to 0.025 ml/min at an elution volume of 1.63–2.30 ml. X-ray scattering was collected every 20 s on a PILATUS3 2 M detector over an angular range of *q*_min_ = 0.00690 Å^−1^ to *q*_max_ = 0.27815 Å^−1^. UV spectra at a range of 200–450 nm were recorded every 10 s. Circular averaging and buffer subtraction were carried out using the program SAngler^58^ to obtain one-dimensional scattering data *I*(*q*) as a function of *q* (*q* = 4*π*sin*θ*/*λ*, where 2*θ* is the scattering angle and *λ* is the X-ray wavelength 1.5 Å). The scattering intensity was normalized on an absolute scale using the scattering intensity of water^59^. The multiple concentrations of the scattering data around the peak at A_280_ and *I*(0) were extrapolated to zero-concentration using a Serial Analyzer^60^. The molecular weights of samples were calculated from the *I*(*q*) data of Ovalbumin (Sigma) at the highest values of A_280_ and *I*(0). The radius of gyration *R*_g_ and the forward scattering intensity *I*(0) were estimated from the Guinier plot of *I*(*q*) in the smaller angle region of *qR*_g_ < 1.3. The distance distribution function *P*(*r*) of the sample at the highest peak of A_280_ and *I*(0) was calculated using the program GNOM^61^, where the experimental *I*(*q*) data were used in a *q*-range of 0.00885–0.17670 Å^−1^. The maximum particle dimension *D*_max_ was estimated from the *P*(*r*) function as the distance *r* for which *P*(*r*) = 0. The molecular weight of the sample was estimated by comparing the *I*(0)/*c* (where *c* is the protein concentration) of the sample to that of Ovalbumin.

### Cell culture

The mESC line J1 was originally provided by the laboratory of Dr. Rudolf Jaenisch (Whitehead Institute). *Dnmt1* KO mESCs were described in ref. ^62^ and *Uhrf1* KO mESCs were described in ref. ^36^. All mESC lines were maintained on 0.2% gelatin-coated dishes in Dulbecco’s modified Eagle’s medium (Sigma) supplemented with 16% fetal bovine serum (FBS, Sigma), 0.1 mM ß-mercaptoethanol (Invitrogen), 2 mM L-glutamine (Sigma), 1× Minimum Essential Medium non-essential amino acids (Sigma), 100 U/ml penicillin, 100 mg/ml streptomycin (Sigma), recombinant LIF (ESGRO, Millipore), and 2i (1 mM PD032591 and 3 mM CHIR99021 (Axon Medchem, Netherlands)). Baby hamster kidney (BHK) cells containing a stably integrated lac operator (lacO) array used for the F3H assay were kindly provided by the laboratory of Dr. David L. Spector^63^. BHK cells were grown in a humidified atmosphere at 37 °C and 5% CO_2_ in Dulbecco’s modified Eagle’s medium supplemented with 1 mM Gentamycin (Serva GmbH) and 10% FBS (Sigma). All cell lines were regularly tested for mycoplasma contamination.

### CRISPR/Cas9 gene editing and excision

For generation of *Paf15* K15R and K24R mutant mESCs, specific gRNAs for each mutation were cloned into a modified version of the SpCas9-T2A-Puromycin/gRNA vector (px459;^64^ Addgene plasmid #62988), in which SpCas9 is fused to truncated human Geminin (hGem) to preferentially generate double-strand breaks when homology-directed repair is active^65^. To generate targeting donors for each desired mutation, single-stranded oligonucleotides harboring either the K15R or K24R substitution and ~100 bp homologous to the respective genomic locus were synthesized (IDT, Coralville, IA, USA). Cells were transfected with a 4:1 ratio of donor oligo and Cas9/gRNA construct. RNA vector was obtained via cut-ligation. Two days after transfection, cells were plated at clonal density and subjected to a transient puromycin selection (1 mg/ml) for 40 h. Colonies were picked out 6 days after transfection. Cell lysis in 96-well plates, PCRs of lysates, and restriction digestion were performed as previously described^62^. Successful insertion of *Paf15* K15R and K24R mutations was confirmed by Sanger sequencing. For generation of the *Paf15* K15R/K24R double-mutant ESC lines, three characterized *Paf15* K24R single mutants were subjected to a second round of gene editing to achieve the K15R substitution as described above.

### Quantitative real-time PCR (qRT-PCR) analysis

Total RNA was isolated using a NucleoSpin Triprep Kit (Macherey-Nagel) according to the manufacturer’s instructions. cDNA synthesis was performed with a High-Capacity cDNA Reverse Transcription Kit (with RNase Inhibitor; Applied Biosystems) using 2 µg of total RNA as input. qRT-PCR assays with the oligonucleotides listed in Supplementary Table 3 were performed in 8 µl reaction volumes with 5 ng of cDNA used as input. For SYBR green detection, FastStart Universal SYBR Green Master Mix (Roche) was used. The reactions were run on a LightCycler480 (Roche).

### Co-IP and western blotting of mouse samples

For co-IP of DNMT1, 1.5 × 10^7^ of mESCs were lysed in 250 µl of lysis buffer (10 mM Tris/Cl pH7.5, 150 mM NaCl, 0.5 mM EDTA, 0.5% NP40, 1.5 mM MgCl_2_, 0.5 µg/ml Benzonase (Sigma-Aldrich), 1 mM PMSF, 1× mammalian Protease Inhibitor Cocktail (e.g., Serva®), 5 mM NEM (Sigma)) at 4 °C for 30 min. Lysates were cleared by centrifugation at 20,000 × *g* for 15 min at 4 °C, and the protein concentration was measured using Pierce™ 660 nm Protein Assay Reagent according to the manufacturer’s instructions. For DNMT1 IP, we used an anti-DNMT1 nanobody (commercial name: DNMT1-Trap, ChromoTek), which is an antigen-binding domain (V_H_H) derived from the heavy chain of an alpaca antibody raised against DNMT1. Equal amounts of protein extracts were incubated with 25 µl of DNMT1-Trap (undiluted) for 2 h at 4 °C under constant rotation. Beads were washed three times with washing buffer (10 mM Tris/Cl pH7.5, 150 mM NaCl, 0.5 mM EDTA) and boiled in Laemmli buffer at 95 °C for 10 min. Bound fractions were separated and visualized as a western blot.

To isolate cytoplasmic and nuclear fractions, 2 × 10^7^ of mESCs were treated with 400 µl of hypotonic buffer (10 mM Tris-HCl pH 8, 10 mM KCl, 1.5 mM MgCl_2_, 1 mM DTT, 1× Protease Inhibitor, 2 mM PMSF, 5 mM NEM, and 0.1% Triton X-100) at 4 °C for 5 min. The cytoplasmic fraction was separated from nuclei by centrifugation at 1300 × *g* for 10 min at 4 °C, then supplemented with 150 mM NaCl and clarified by centrifugation at 20,000 × *g* for 15 min at 4 °C. Nuclei were lysed as described above. Anti-mPAF15 antibody (Santa Cruz, sc-390515, 2 µg) was added to the cytoplasmic and nuclear lysate and incubated for 2 h at 4 °C under constant rotation. To precipitate mPAF15-bound proteins, 20 µl of protein G beads (GE17-0618-06) were added to the lysate for an overnight incubation at 4 °C under constant rotation.

Western blots for mDNMT1 were performed as described previously^18^ using a monoclonal antibody (rat anti-DNMT1, 14F6, 1:10 dilution) and a polyclonal antibody (rabbit anti-DNMT1, Abcam, ab87654, 1:2500 dilution). Other antibodies used for detection were mouse anti-PAF15 antibody (Santa Cruz, sc-390515, 1:1500 dilution), polyclonal rabbit-anti-H3 (Abcam, ab1791, 1:5000 dilution), and a monoclonal mouse-anti-tubulin (Sigma, T9026, 1:2000 dilution). The following secondary antibodies conjugated to horseradish peroxidase were used: goat polyclonal anti-rat IgG (Dianova, 112-035-003, 1:5000), goat polyclonal anti-rabbit IgG (Bio-rad), and rabbit polyclonal anti-mouse IgG (Sigma, A9044, 1:5000). For detection of horseradish peroxidase-conjugated antibodies, an ECL Plus reagent (GE Healthcare, Thermo Scientific) was used.

### Reduced representation bisulfite sequencing

For RRBS, genomic DNA was isolated using a QIAamp DNA Mini Kit (QIAGEN), after an overnight lysis and proteinase K treatment. Preparation of the RRBS library was carried out as described previously^66^, with the following modifications: bisulfite treatment was performed using an EZ DNA Methylation-Gold™ Kit (Zymo Research Corporation) according to the manufacturer’s protocol except that libraries were eluted in 2× 20 mL M-elution buffer. RRBS libraries were sequenced on an Illumina HiSeq 1500 in 50 bp paired-end mode.

### RRBS alignment and analysis

Raw RRBS reads were first trimmed using Trim Galore (v.0.3.1) with the “--rrbs” parameter. Alignments were carried out with the mouse genome (mm10) using bsmap (v.2.90) and the parameters “-s 12 -v 10 -r 2 -I 1.” CpG-methylation calls were extracted from the mapping output using bsmaps methratio.py. Analysis was restricted to CpG with a coverage >10. A methylKit^67^ was used to identify differentially methylated regions between the respective contrasts for the following genomic features: (1) repeats (defined by Repbase), (2) gene promoters (defined as gene start sites −2 kb/+2 kb), and (3) gene bodies (defined as the longest isoform per gene) and CpG islands (as defined by ref. ^68^). Differentially methylated regions were identified as regions with *p* < 0.05 and a difference in methylation means between two groups >25%.

### Data processing and analysis

Chromatin IP–sequencing reads for H3K9me2^38^, H3K9me3^39^ and H3K14ac^40^ in ESCs and EpiLCs were downloaded from GSE6020467, GSE2394368, and GSE3128469, respectively. Reads were aligned to the mouse genome (mm10) with Bowtie (v.1.2.2) with parameters “-a -m 3 -n 3 -best -strata.” Peak calling and signal pile-up was performed using MACS2 callpeak^69^ with the parameters “-extsize 150‘-nomodel -B -nolambda” for all samples. Tag densities for 1 kb Tiles detected in RBBS were calculated using custom R scripts. Replication domain data for mouse ESCs (mm10) for replication timing analysis was taken from http://www.replicationdomain.org/^69^. The average replication timing ratio was calculated over 1 kb Tiles detected in RBBS using custom R scripts. Data of partially methylated domains and highly methylated domains (mm10) was downloaded from https://zwdzwd.github.io/pmd^42^ and used to calculate average DNA methylation levels (RRBS) over these regions.

### High-throughput immunofluorescence and image analysis

ESCs were grown in 96-well microplates (μClear, Greiner Bio-One), washed with PBS, and fixed with 3.7% formaldehyde. After three washing steps with PBST, cells were permeabilized (0.5 % Triton-X100), treated with denaturing solution (2 N HCl) for 40 min, and incubated with renaturing solution (150 mM Tris-HCl, pH 8.5) for 20 min. Cells were then blocked in 2% bovine serum albumin for 1 h and incubated with primary antibody (mouse-anti 5mC, Diagenode 33D3) for 1 h at 37 °C. After washing three times with PBST, cells were incubated with secondary antibody (goat-anti-mouse coupled to Alexa647, Thermo Fisher) for 1 h at 37 °C. Cells were washed three times with PBST, counterstained with 200 ng/ml 4,6-diamidino-2-phenylindole (DAPI), and finally covered with PBS. Images were acquired by automation with an Operetta High-Content Image Analysis System (PerkinElmer, ×40 high NA objective) followed by analysis with the Harmony software (PerkinElmer). DAPI was used for the detection of single nuclei and 5mC modifications were measured in selected nuclei based on the antibody signal intensity.

### F3H assay

The F3H assay was performed as described previously^37^. In brief, BHK cells containing multiple lac operator repeats were transiently transfected on coverslips using polyethyleneimine and fixed with 3.7% formaldehyde 24 h after transfection. For DNA counterstaining, coverslips were incubated in a solution of DAPI (200 ng/ml) in PBST and mounted in Vectashield. Cell images were collected using a Leica TCS SP5 confocal microscope. To quantify the interactions within the lac spot, the following intensity ratio was calculated for each cell: (mCherry_spot_ − mCherry_background_)/(GFP_spot_ − GFP_background_) in order to account for different expression levels. The following constructs used in the F3H assay have been described previously: pCAG-eGFP-IB^70^, pCAG-eGFP-mDNMT1^70^, and pGBP-LacI^37^. To generate the mCherry-mPAF15 WT and KRKR expression constructs, the coding sequences of mPAF15 WT and KRKR were excised via AsiSI and NotI restriction digest from the GFP-PAF15 WT and KRKR constructs^36^ and ligated into the pCAG‐Cherry‐IB vector^70^.

### Reporting summary

Further information on research design is available in the Nature Research Reporting Summary linked to this article.

## Supplementary information

Supplementary Information

Peer Review

Reporting Summary

Description of Additional Supplementary Files

Supplementary Data 1

Supplementary Data 2

Supplementary Data 3

## Data Availability

The data that support this study are available from the corresponding authors upon reasonable request. The crystal structures of the human UHRF1 PHD in complex with PAF15(2-11) has been deposited in the Protein Data Bank under accession code 6IIW. Sequencing data reported in this paper (wt and PAF15KRKR RRBS) are available at ArrayExpress (EMBL-EBI) under accession number E-MTAB-7930. The mass spectrometric proteomics data have been deposited at the ProteomeXchange Consortium via the PRIDE partner repository with dataset identifier PXD015282. The source data underlying Figs. 1b–e, 2a–f, 3a–d, 4a–e, f, and 5a, b, e and Supplementary Figs. 1b–d, f–g, 2a–c, 3a, b, d, e, 4a–f, 5c, d, g, and 6a–c are provided as a Source Data file.

## Source Data

Source Data
