# Supplementary material for: Two distinct modes of DNMT1 recruitment ensure stable maintenance DNA methylation
Source: Nat Commun. 2020 Mar 6;11:1222. doi: 10.1038/s41467-020-15006-4 (PMC7060239; doi:10.1038/s41467-020-15006-4)
Supplement: Supplementary file 1 — Supplementary Information [file 41467_2020_15006_MOESM1_ESM.pdf]

Supplementary Information

Two distinct modes of DNMT1 recruitment ensure stable maintenance DNA methylation

Nishiyama et al.

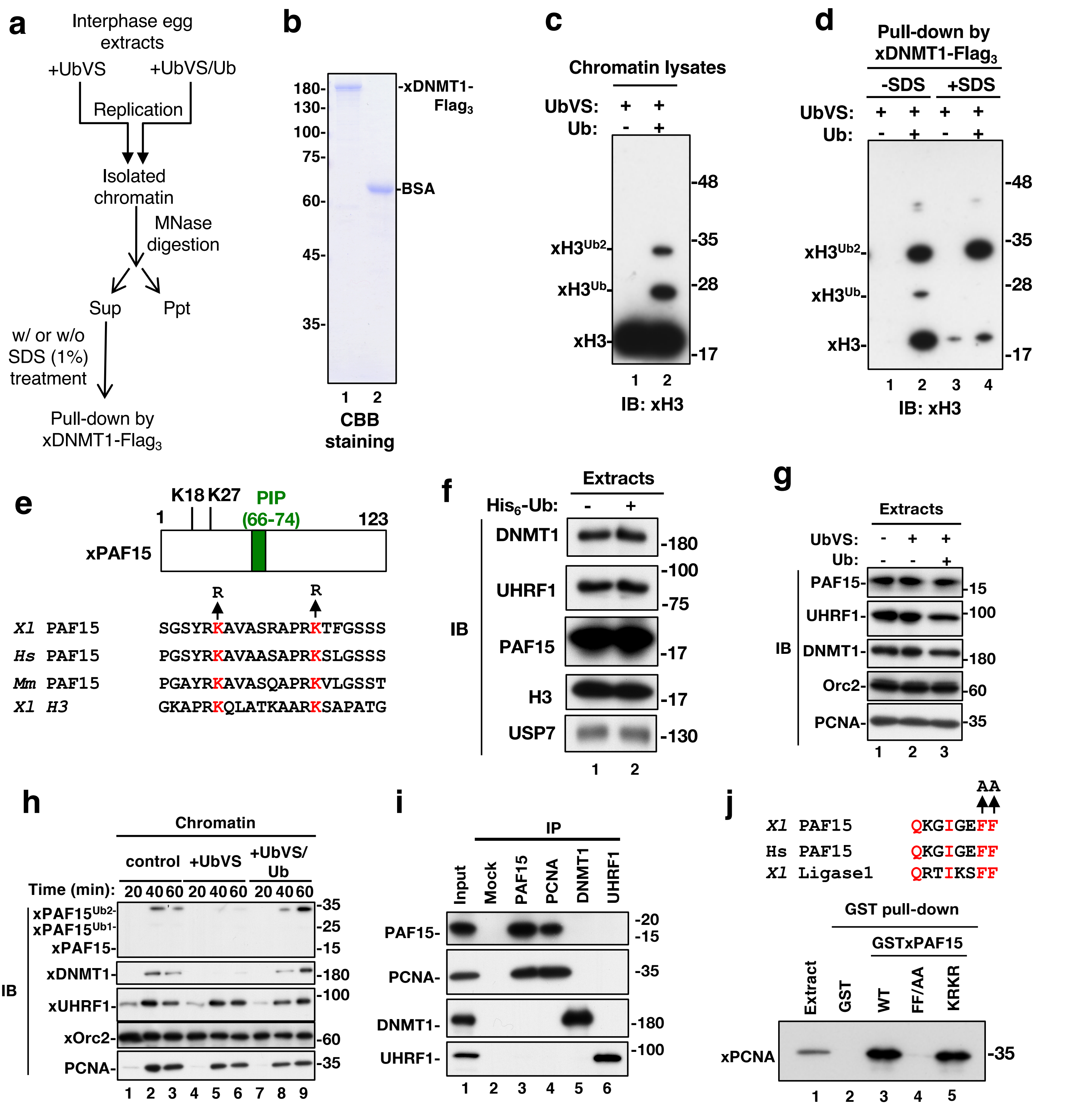

Supplementary Fig. 1 | Isolation of ubiquitin signaling-dependent DNMT1-interacting proteins from chromatin lysates, and characterization of xPAF15. a, Schematic of assay to isolate DNMT1-interacting proteins from chromatin lysates. **b,** 1 µg of recombinant xDNMT1-Flag3 (lane 1), and BSA were separated by SDS-PAGE, and gel was stained with CBB. **c**, Sperm chromatin was added to interphase egg extracts pretreated with UbVS (14 µM) in the presence or absence of free ubiquitin (58 µM). Isolated chromatin fractions were subjected to MNase digestion and solubilized proteins were analyzed by immunoblotting using anti-histone H3 antibody. **d**, Chromatin lysates were subjected to a pull-down experiment using Flag-tagged recombinant wild-type xDNMT1 coupled with anti-Flag M2 beads. The resultant immunoprecipitates were analyzed by immunoblotting using anti-H3 antibody. **e**, The domain structure of xPAF15 and sequence alignment of the conserved ubiquitylation sites across different species. Ubiquitylation sites are shown in red. **f and g,** Extracts used in Fig. 1b and Supplementary Fig. 1h were analyzed by immunoblotting using indicated antibodies. **h**, Sperm chromatin was replicated in interphase egg extracts containing buffer (lanes 1-3) or 14 µM UbVS in the absence (lanes 4-6) or presence of 58 µM recombinant ubiquitin (lanes 7-9), and chromatin-associated proteins were analyzed by immunoblotting using the indicated antibodies. **i,** Immunoprecipitates from *Xenopus* interphase egg extracts using anti-xPAF15 (lane 3), anti-xPCNA (lane 4), anti-xUHRF1 (lane 5), and anti-xDNMT1 (lane 6) antibodies, or control IgG (lane 2) as well as egg extracts (lane 1) were subjected to immunoblotting using the antibodies indicated. **j**, Sequence alignment of the PIP box of PAF15. A PIP box of *Xenopus* DNA ligase 1 (Lig1) is also aligned. Red residues in the PIP boxes are conserved. GST-tagged full-length xPAF15 wild-type (WT, lane 3), F72AF73A(FF/AA, lane 4), or K18RK27R(KRKR, lane 5) were immobilized on GSH beads and incubated with interphase egg extracts. Bound proteins were analyzed by immunoblotting with PCNA antibodies. Source data are provided as a Source Data file.

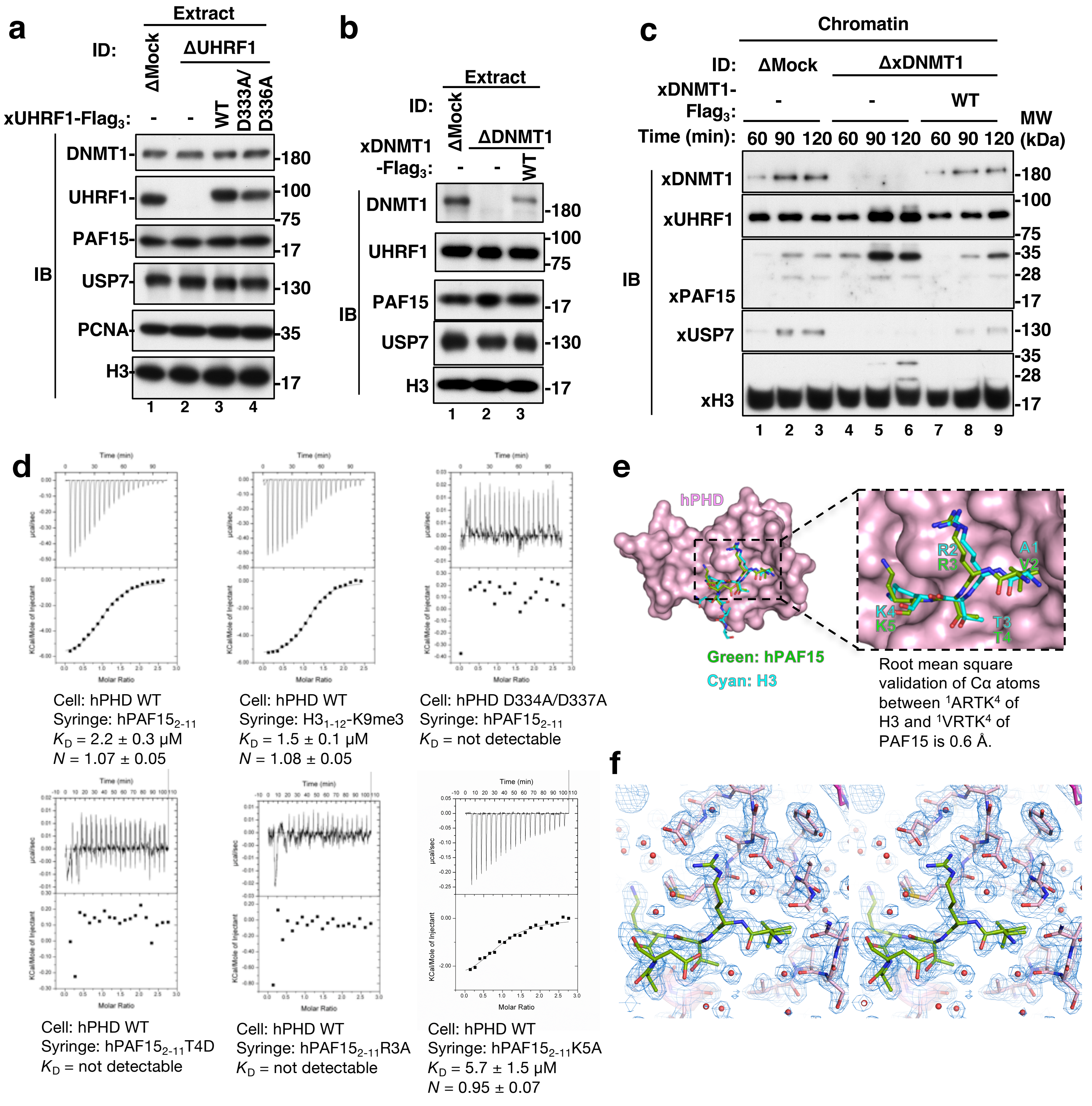

Supplementary Fig. 2 | UHRF1-dependent regulation of PAF15 in egg extracts, ITC thermograms and a structural comparison

a, Mock- and UHRF1-depleted extracts used as shown in Fig. 2a were analyzed by immunoblotting using the indicated antibodies. **b,** Mock- and DNMT1-depleted extracts used in **c** were analyzed by immunoblotting using the indicated antibodies. **c,** Mock-depleted or DNMT1-depleted extracts were supplemented with the indicated recombinant proteins (wt xDNMT1) and chromatin was isolated. Chromatin-bound proteins were analyzed by immunoblotting using the indicated antibodies. **d,** Representative ITC thermograms (upper) and plots of corrected heat values (lower) for the indicated binding experiments. The first data point of each measurement was omitted from the plots in the lower panels and parameter fittings. **e,** Crystal structure of hPHD (pink surface model) in complex with PAF15$_{2-11}$ is superposed on that in complex with H3 peptide (PDB: 3ASL[https://www.rcsb.org/structure/3ASL]). Close-up view shows a structural comparison of the 1-4 N-terminal residues of H3 (cyan) and PAF15 (green). **f,** Stereo view of the PAF15 recognition site of hPHD.

hPHD and PAF15 are depicted as light-pink and green stick models, respectively. water molecules are shown as red sphere. 2|Fo| - |Fc| map contoured at 1.0 σ (light-blue) is superimposed on the models. Source data are provided as a Source Data file.

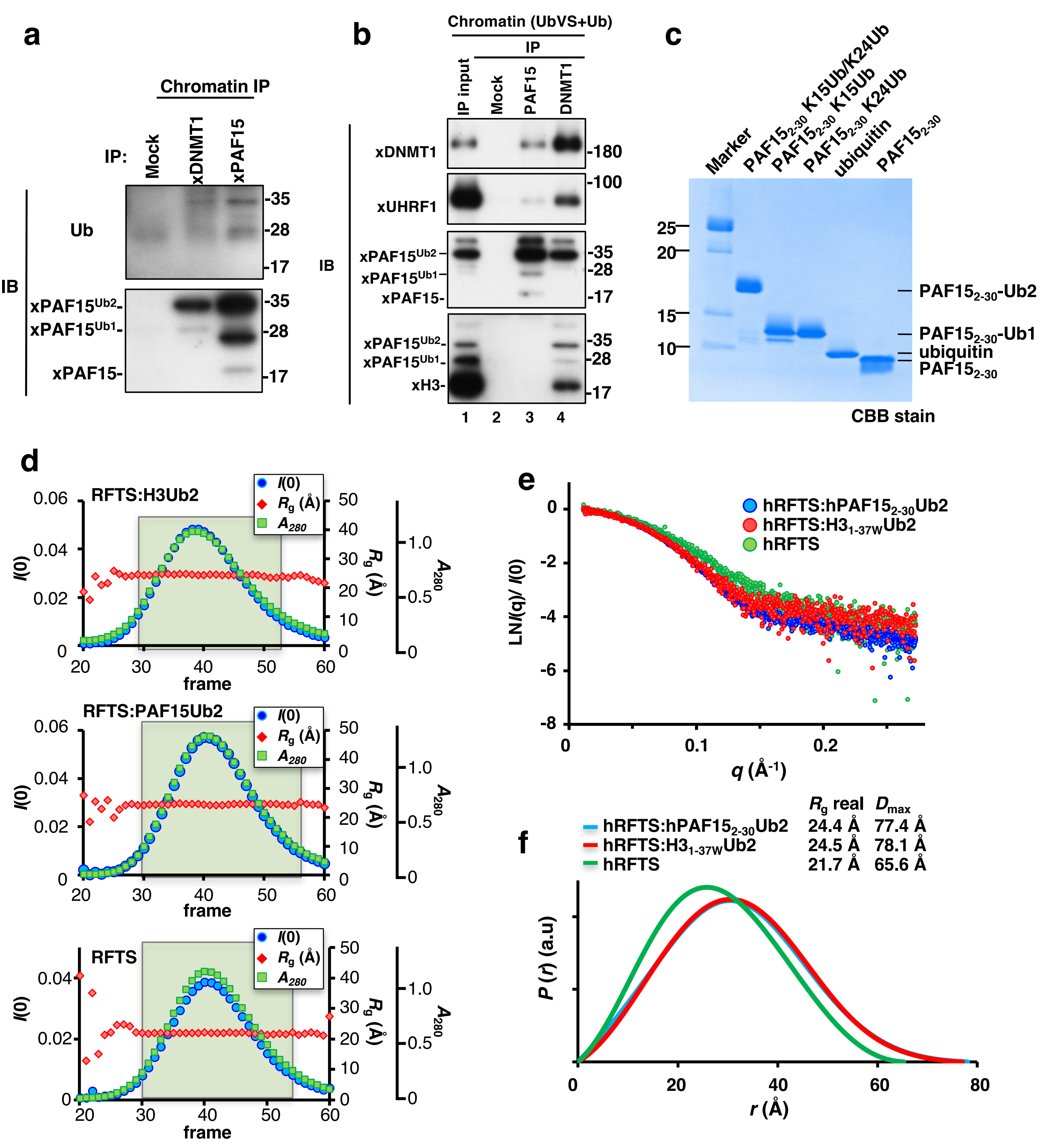

Supplementary Fig. 3 | DNMT1 specifically interacts with two mono-ubiquitylated PAF15

a, Immunoprecipitates from chromatin lysates (Chromatin-IP) with control (Mock), anti-xDNMT1 (DNMT1), or anti-xPAF15 (PAF15) antibody were analyzed by immunoblotting. **b**, Sperm chromatin was replicated in interphase egg extracts containing 14 μM UbVS and 0.2 mg/ml ubiquitin. Isolated and solubilized chromatin proteins were subjected to immunoprecipitation using anti-PAF15 or DNMT1 antibodies. The resultant immunoprecipitates were analyzed by immunoblotting using the indicated antibodies. c, Purity check of disulfide-mediated ubiquitinated PAF15 analyzed by SDS-PAGE under oxidative condition. **d**, R_g (blue) and $I(0)$ (red) plots for SEC-SAXS of hRFTS:H3$_{1\text{-}37W}$Ub2 (top), hRFTS:PAF15$_{2\text{-}30}$Ub2 (middle), and RFTS (bottom). X-ray scattering frames highlighted as green were used for extrapolation to zero-concentrations. **e,** Experimental X-ray scattering curves of hRFTS:hPAF15$_{2\text{-}30}$Ub2 (cyan circle), hRFTS:H3$_{1\text{-}37W}$Ub2 (red circle) and apo-hRFTS (green circle). The q-range is scattering curves are collected from 0.0113 to 0.2729 Å^{-1}. Vertical and horizontal axes indicate absolute intensity $\ln I(q)/I(0)$ and scattering angle $q = 4\pi\sin\theta/\lambda$, respectively. **f**, Pair distance distribution functions $P(r)$ of hRFTS:PAF15$_{2\text{-}30}$Ub2 (cyan), hRFTS:H3$_{1\text{-}37W}$Ub2 (red) and hRFTS alone (green) determined from SAXS data. The $P(r)$ functions were normalized by $I(0)$ calculated from each scatter plot. Source data are provided as a Source Data file.

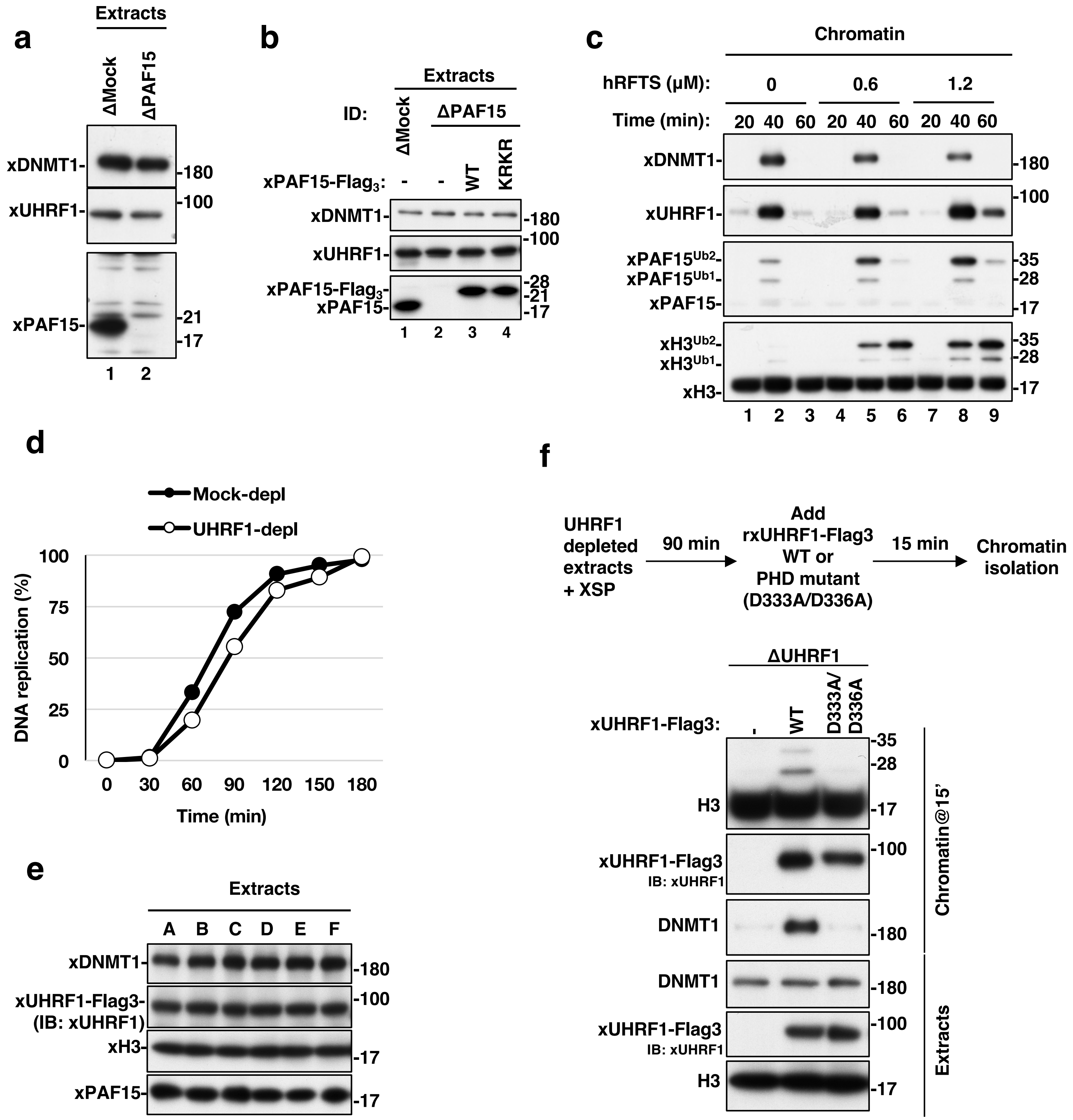

Supplementary Fig. 4 | Immunodepletion of xPAF15 or UHRF1 from *Xenopus* interphase egg extracts and effect of recombinant hRFTS addition to *Xenopus* egg extracts

a, 0.25 μl of mock-depleted or xPAF15-depleted interphase egg extracts were immunoblotted with the indicated antibodies. **b,** Mock- and PAF15-depleted extracts used in Figure 4b were analyzed by immunoblotting using the indicated antibodies. **c**, Interphase egg extracts containing 0.6 or 1.2 μM hRFTS were treated with sperm chromatin. Chromatin fractions were isolated at the indicated times and analyzed by immunoblotting. d, Sperm chromatin was incubated in the indicated interphase egg extracts in the presence of [α-^{32}P]dCTP. The percentage of input DNA replicated at various times is plotted. **e**, Extracts used in Fig. 4f and g were analyzed by immunoblotting using the indicated antibodies. **f**, Schematic of experimental approach to test the role of UHRF1 in histone H3 ubiquitylation. Sperm chromatin was added to xUHRF1-depleted extracts which were incubated for 90 min. The extracts were then supplemented with recombinant xUHRF1-Flag3-WT or –D333A/D336A and further incubated for 15 min. Chromatin fractions were isolated and chromatin-bound proteins were analyzed by immunoblotting using the antibodies indicated. Extracts were also analyzed by immunoblotting. Source data are provided as a Source Data file.

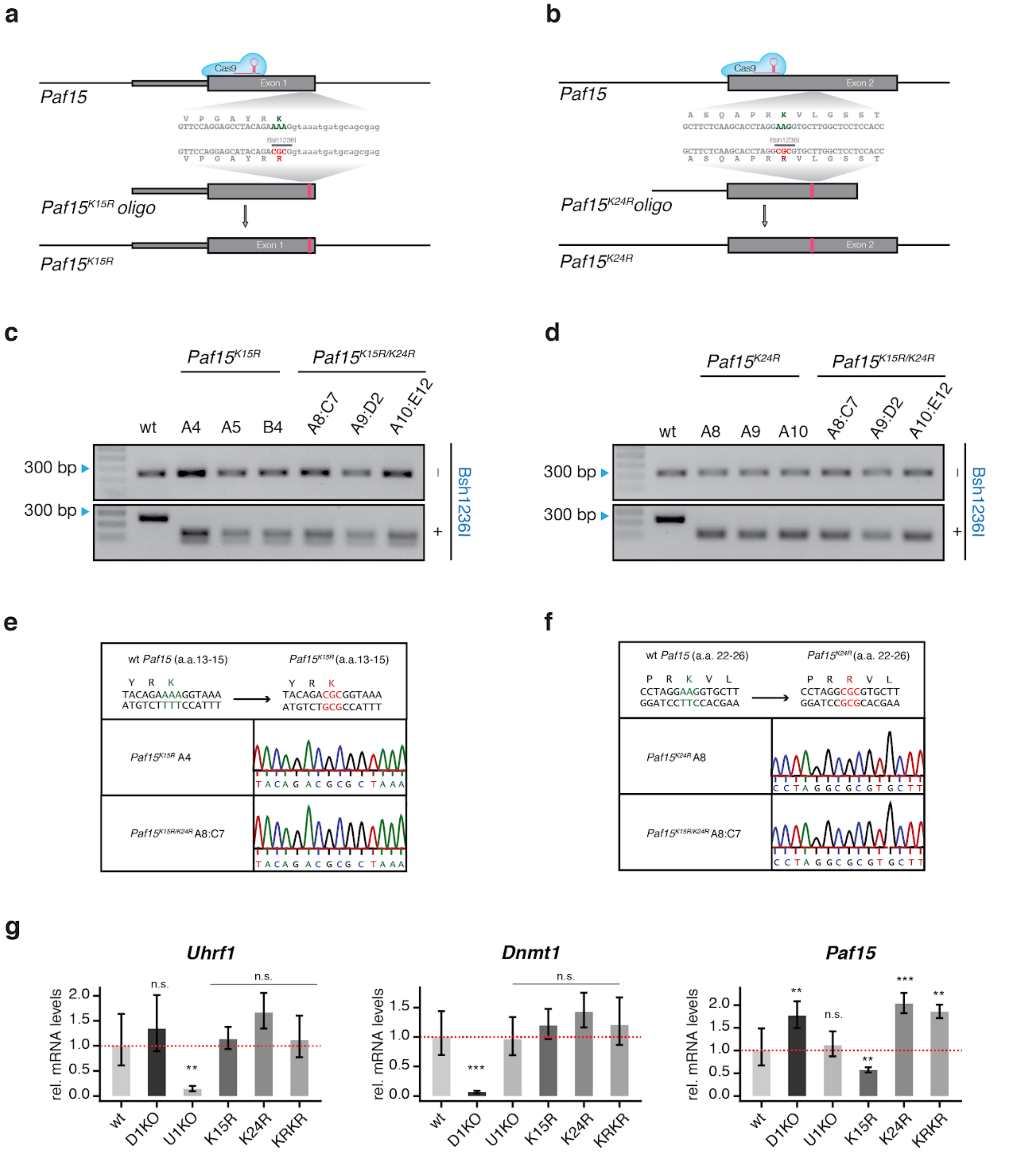

Supplementary Fig. 5 | Generation and characterization of *Paf15*^{K15R}, *Paf15*^{K24R}, and *Paf15*$^{K15R/K24R}$ (KRKR) mutant mESCs.

a,**b**. Schematic representation of the CRISPR/Cas9 gene editing strategy used for generating Paf15 (**a**) K15R and (**b**) K24R substitutions in mouse embryonic stem cells (mESCs). Restriction enzyme recognition sites generated by gene editing for restriction fragment length polymorphism (RFLP) screening and amino acid substitutions are shown. **c**,**d**. Genotyping of (**c**) *Paf15*^{K15R} and (**d**) *Paf15*^{K24R} mutant clones via RFLP analysis. **e**,**f.** Confirmation of successful insertion of (**e**) K15R and (**f**) K24R substitutions in the indicated *Paf15* mutants as assessed by Sanger sequencing. **g.** Expression of *Uhrf1*, *Dnmt1*, and *Paf15* in wt, *Dnmt1* KO (D1KO), *Uhrf1* KO (U1 KO), *Paf15*^{K15R} (K15R), *Paf15*^{K24R} (K24R) and *Paf15*$^{K15R/K24R}$ (KRKR) mESCs as assessed by qRT-PCR. Error bars represent means ± SD from n=3 biological replicates. $^{**}P < 0.01$, $^{***}P < 0.001$, P-values calculated by two-tailed Student's t-test between wild-type and the indicated mutant. n.s., not significant. Source data are provided as a Source Data file.

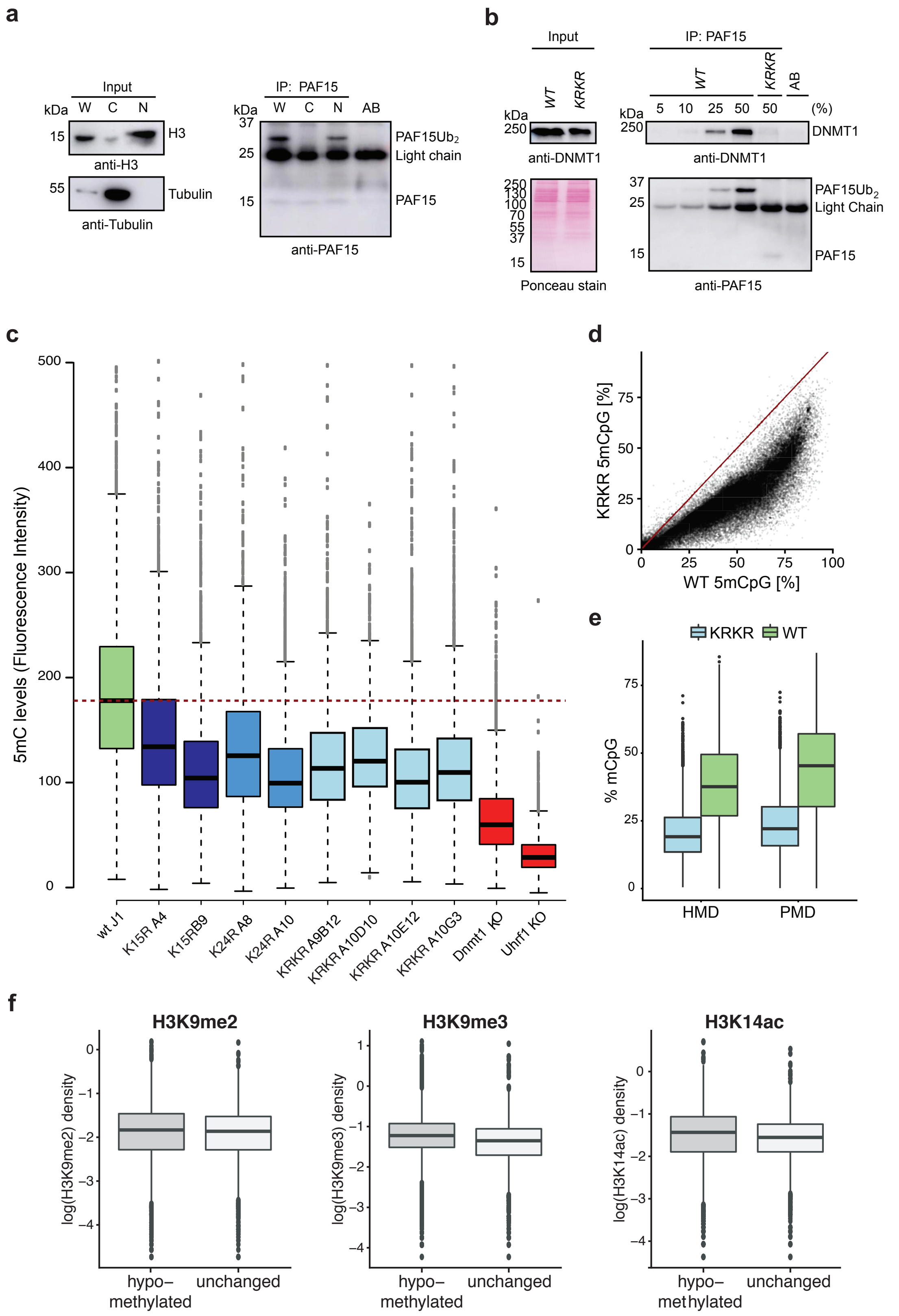

Supplementary Figure 6: PAF15 dual-monoubiquitylation is critical for interacting with DNMT1 and maintaining global DNA methylation in mouse ESCs
a, Whole-cell (W), cytoplasmic (C), and nuclear (N) extracts from wild-type mESCs were subjected to immunoprecipitation of endogenous mPAF15 using an anti-mPAF15 antibody. mPAF15 was detected in the bound fraction with an anti-mPAF15 antibody. mH3 and mTubulin blots were used as indicators of successful nuclear and cytoplasmic fractionation. AB, antibody. **b,** Immunoprecipitation of endogenous mPAF15 from wild-type (WT) and *Paf15* KRKR(KRKR) mESC nuclear extracts using an anti-mPAF15 antibody. Bound fractions were subjected to immunoblotting with anti-mDNMT1 and anti-mPAF15 antibodies. The anti-mDNMT1 blot and Ponceau staining are shown as loading controls. Prior to loading, anti-mPAF15 immunoprecipitated material from wild-type ESCs was titrated (percentage indicated) to achieve levels of mPAF15 comparable to those from *Paf15* KRKR ESCs. AB, antibody. **c,** Quantification of anti-5mC staining in wild-type, *Dnmt1* KO, *Uhrf1* KO ESCs and two independent clones of *Paf15*K15R (K15R), *Paf15*K24R (K24R) and *Paf15*K15R/K24R (KRKR) mutant ESCs with n = 500-2500 cells per replicate. **d,** Comparison of the DNA methylation levels of individual CpG sites in wild-type and *Paf15* KRKR ESCs. **e,** DNA methylation levels of highly methylated domains (HMDs) and partially methylated domains (PMDs) in *Paf15* KRKR and wild-type (WT) ESCs. **f,** H3K9me2, H3K9me3 and H3K14ac density (log10) at hypomethylated and unchanged tiles of *Paf15* KRKR mESCs. Differentially methylated tiles losing DNA methylation (hypomethylated tiles) were defined as those with $P<0.05$ and a methylation loss >25%; P-values were derived from methylKit package (see Materials and Methods). For the boxplots in c,e and f, the horizontal black lines within boxes represent median values, boxes indicate the upper and lower quartiles, and whiskers indicate the 1.5x interquartile range. Source data are provided as a Source Data file.

Supplementary Table 1. SAXS data collection parameters

	**hRFTS: $hPAF15_{2\text{-}30}Ub2$**	**hRFTS: $H3_{1\text{-}37W}$-Ub2**	**hRFTS**	**Ovalbumin**
**Data collection parameters**				
Instrument	Photon Factory BL-10C			
Wavelength (Å)	1.5			
q range ($Å^{-1}$)	0.0069–0.2781			
Detector	Pilatus3 2 M			
Detector distance (mm)	2,027			
Exposure (s per image)	20			
SEC Column	5/150GL INCREASE Superdex200			
Flow rate ($mL.min^{-1}$)	0.025			
Injected sample conc. ($mg.mL^{-1}$)	6.0			
Injection volume (μL)	50			
Temperature (K)	277			
**Structural parameters (data of extrapolated to zero concentration)**				
R_g (Å) [from $P(r)$]	24.4 ± 0.2	24.5 ± 0.2	21.7 ± 0.1	24.3 ± 0.1
R_g (Å) [from Guinier]	24.4 ± 0.2	24.3 ± 0.2	21.7 ± 0.2	24.1 ± 0.2
D_{max} (Å)	77.4	78.1	65.6	75.1
Porod volume estimate ($Å^3$)	59,300	57,700	36700	63,300
**Molecular-mass determination**				
*Molecular mass M_r [$I(0)/c$ of standard]	43.9 ± 1	43.9 ± 3	29.6 ± 1	-
*$I(0)/^{\#}c$ [from Guinier]	0.046204 ± 0.001351	0.046238 ± 0.003432	0.031144 ± 0.001024	0.04679
Calculated M_r from sequence	48.0	48.9	28.0	44.4
**Software employed**				
Primary data reduction	SAnglar			
Data processing	PRIMUS			
Ab initio analysis	DAMMIF			
Validation and averaging	DAMAVER & DAMMIN			
Computation of model intensities	CRYSOL			

[1]average and deviation are derived from the indicated frames in Supplementary Fig. 3d.

[2]sample concentration (mg/ml)

Supplementary Table 2: RRBS Information				
Sample_ID	Genotype	# total reads	#mapped reads	% of mapped reads
KRKR_1	PAF15_KRKR_ESC	28,140,727.00	19135694	68
KRKR_2	PAF15_KRKR_ESC	35,670,848.00	26753136	75
WT-1	wt_J1_ESC	33,012,500.00	24429250	74
WT-2	wt_J1_ESC	21,910,914.00	15118530	69
D1KO_r1	DNMT1_KO_ESC	3,686,943.00	2617730	71
D1KO_r2	DNMT1_KO_ESC	3,846,150.00	2499998	65
D1KO_r3	DNMT1_KO_ESC	3,688,377.00	2508097	68
U1KO_r1	UHRF1_KO_ESC	3,248,968.00	2436726	75
U1KO_r2	UHRF1_KO_ESC	3,581,465.00	2399582	67
U1KO_r3	UHRF1_KO_ESC	3,680,511.00	2539553	69
wt_r1	wt_J1_ESC	3,229,351.00	2615775	81
wt_r2	wt_J1_ESC	2,956,850.00	2247206	76
wt_r3	wt_J1_ESC	3,081,518.00	2280324	74

combined coverage of all samples over genomic elements	
Genomic Element	coverage [fraction of total]
Repeats	0.013915533
CpG islands	0.610142975
Promoters	0.430718894
Gene bodies	0.445765294

Supplementary Table 3: Oligonucleotides used in this study

**Name**	**Sequence 5′-3′**	**Description**
3621	ATAGCGCTGGAGGGAATTCAGTGTAACGCA	xPAF15 amplification
3622	AAAAGCAGCATGAATGCTCTAGTCCAGGCTT	xPAF15 amplification
3667	CGTGGATCCCCGAATTCCATGGTGCGGACTAAGGCAGA	GST-xPAF15
3636	GGCCGCTCGAGTCGATTATTTACAAATATACAAAGC	GST-xPAF15
3720	ggcgcggatcagatctcATGGTGCGGACTAAGGCAGACT	pVL1392-xPAF15-Flag3
3581	GGGCCCTCTAGAATTCTACTTGTTATCGTCATCCT	pVL1392-xPAF15-Flag3
3707	AgGGCTGTTGCTGCCAGAGCACCAAGGA	xPAF15 K18R mutation
3708	CCTGTAGCTCCCCGATGAAGAGCCC	xPAF15 K18R mutation
3709	AgAACATTTGGGAGTAGTTCCAGTGGTT	xPAF15 K27R mutation
3710	CCTTGGTGCTCTGGCAGCAACAGCC	xPAF15 K27R mutation
3711	gcCgcTGGATCACCATCCACAAGTCAGCCTG	xPAF15F72AF73A mutation
3712	GTCTCCTATACCTTTCTGCCAGGTA	xPAF15F72AF73A mutation
3896	gcGACTAAGGCAGACTGCGCGGGCTCTT	pKS104-xPAF15R3A mutation
3897	CACCATGAATTCTCGAGTGCAAAAA	xPAF15R3A mutation
3898	CGGgaTAAGGCAGACTGCGCGGGCTCTT	xPAF15T3D mutation
3899	CGGACTgcGGCAGACTGCGCGGGCTCTT	xPAF15K4A mutation
4449	CACCATGAGATCTGATCCGCGCCCG	pVL1392-xPAF15R3A mutation
4620	GGCGCGGATCAGATCTCATGTGGATACAGGTGCGTAC	pVL1392-xUHRF1-Flag3
4271	GCCATGGCGTTTCACATTTATTGCCTTA	xUHRF1D333A mutation
4272	ACACTCATCACAAAGAAGTTGTTTC	xUHRF1D333A mutation
4273	GCTGAGTGTGCCATGGCGTTTCACATTT	xUHRF1D333AD336A mutation
4274	ACAAAGAAGTTGTTTCTCTGGGTCC	xUHRF1D333AD336A mutation
Dnmt1_F	GGCGGAAATCAAAGGAGGAT	RT-qPCR
Dnmt1_R	CCTGGGTCTGGAACTTCTTTTATC	RT-qPCR
Uhrf1_F	GGCAGCTGAAGCGGATGA	RT-qPCR
Uhrf1_R	CCATGCACCGAAGATATTGTCA	RT-qPCR

PAF15_F	CAAGTTCGTCGAGAAAAGCTGA	RT-qPCR
PAF15_R	ACAGCCTGAAGAATTCCCCG	RT-qPCR
Gapdh_F	CATGGCCTTCCGTGTTCCTA	RT-qPCR
Gapdh_R	CTTCACCACCTTCTTGATGTCATC	RT-qPCR
PAF15_K15_gRNA_F	CACCGCATCATTTACCTTTTCTGT	Cloning gRNAs
PAF15_K15_gRNA_R	aaacACAGAAAAGGTAAATGATGC	Cloning gRNAs
PAF15_K24_gRNA_F	CACCGGAGCCAAGCACCTTCCTAG	Cloning gRNAs
PAF15_K24_gRNA_R	aaacCTAGGAAGGTGCTTGGCTCC	Cloning gRNAs
PAF15_K15_scrF	CGGGAAAGAGACCCATTTAAAC	PCR and RFLP Analysis
PAF15_K15_scrR	GCCTTCTAGCTGCTCAATGG	PCR and RFLP Analysis
PAF15_K24_scrF	CTGGCCTGGGACTGTTGTAG	PCR and RFLP Analysis
PAF15_K24_scrR	CAGGTTAGTACTGCCTTGCC	PCR and RFLP Analysis
Paf15_K15R_Donor	CCTGCCTTCTAGCTGCTCAATGGGAGGCAGCCATGGGCGTCTCCACCCCT GGACAGGCTGCCTAGGGAACCCCCTGCCACCTCGCTGCATCATTTACCgc gTCTGTATGCTCCTGGAACGTAGTTTGCTTTGGTCCGCACCATGTTCACA CAAGAAGAGACAACTTTCACCGTCACCCCAACTGCAGATGTCTCAATTAG	ssDNA Donor Oligonucleotides
Paf15_K24R_Donor	ATGCTCTCGGGGTGTTACTTCAGAAGCTTCCACGACCCTTCCTACCTTTT CTCGACGAACTTGAAGAATTGGTGACAAAGGTGGAGGAGCCAAGCACgcg CCTAGGTGCTTGAGAAGCCACCGCTGCAGAGAGAGATAAATAGGGGCGTT CAGAAAAGGCAGGAGGGTTCGGATCCCCGAGCTTTGTTCTACAACAGTC	ssDNA Donor Oligonucleotides
hPAF15_F	ATGGTGCGGACTAAAGCAGACAGTGTTCCAGGCACTTACAGAAAA	hPAF15 amplification
hPAF15_R	CTATTCTTTTTCATCATTTGTGTGATCAGGTTGCAAAGGACATGC	hPAF15 amplification
hPAF15_72stop_F	TAGGTTGTAACCTAAAGATTCTGAAAAAGA	$hPAF15_{2-71}$
hPAF15_72stop_R	CTTTAGGTTACAACCTAAAGAATTCTCCAA	$hPAF15_{2-71}$
N^{pro}-hPAF15_F	CATTATGGGTAACTAGTTGCGTGCGGACTAAAGCAGACAGTGTTC	pET21b-N^{pro}-$hPAF15_{2-71}$
N^{pro}-hPAF15_R	TCCGACATTTGGTCTTATTACAACCTAAAGAATTCTCCAATTCCT	pET21b-N^{pro}-$hPAF15_{2-71}$
hPAF15_K15C/K24C-F	TACAGATGTGTGGTGGCTGCTCGAGCCCCCAGATGTGTGCTTGGTTCTTC	hPAF15 K15C/K24C mutation
hPAF15_K15C/K24C-R	AAGCACACATCTGGGGGCTCGAGCAGCCACCACACATCTGTAAGTGCCTG	
hPAF15_C54S-F	CCCCGTTTCCGTGCGCCCAACTCCCAAGTG	hPAF15 C54S mutation
hPAF15_C54S-R	GGCGCACGGAAACGGGGTTCCCTCCTGCAT	
PAF15_HA_F	TACCCATACGATGTTCCAGATTACGCTTAATAAGACCAAATGTCGGATCCACTAGTG	$hPAF15_{2-71}$-HA
PAF15_HA_R	AGCGTAATCTGGAACATCGTATGGGTACAACCTAAAGAATTCTCCAATTCCTTTTTG	$hPAF15_{2-71}$-HA

PAF15_R2A_F	TTGCGTGGCCACTAAAGCAGACAGTGTTCC	hPAF15 R2A mutation
PAF15_R2A_R	CTTTAGTGGCCACGCAACTAGTTACCCATA	hPAF15 R2A mutation
PAF15_T3D_F	CGTGCGGGACAAAGCAGACAGTGTTCCAGG	hPAF15 T3D mutation
PAF15_T3D_R	CTGCTTTGTCCCGCACGCAACTAGTTACCC	hPAF15 T3D mutation
PAF15_K4A_F	GCGGACTGCCGCAGACAGTGTTCCAGGCAC	hPAF15 K4A mutation
PAF15_K4A_R	TGTCTGCGGCAGTCCGCACGCAACTAGTTA	hPAF15 K4A mutation
UBCh5a-F	ATGGCGCTGAAGAGGATTCAGAAAGAATTGAGTGATCTACAGCGC	hUBCh5 amplification
UBCh5a-R	TTACATTGCATATTTCTGAGTCCATTCTCTTGCATGTCTGTTGTA	hUBCh5 amplification
UHRF1_D334A/D337A_F	TGTGCGCTGAGTGCGCCATGGCCTTCCACA	hUHRF1 D334A and D337A mutation
UHRF1_D334A/D337A_R	CCATGGCGCACTCAGCGCACATGAGCTGCT	
UHRF1_H741A_F	GTGCCAGGCCAACGTGTGCAAGGACTGCCT	hUHRF1 H741A mutation
UHRF1_H741A_R	ACACGTTGGCCTGGCACACGGTCGTGATGG	
