# Supplementary material for: Two distinct modes of DNMT1 recruitment ensure stable maintenance DNA methylation
Source: Nat Commun. 2020 Mar 6;11:1222. doi: 10.1038/s41467-020-15006-4 (PMC7060239; doi:10.1038/s41467-020-15006-4)
Supplement: Supplementary file 4 — Description of Additional Supplementary Files [file 41467_2020_15006_MOESM4_ESM.pdf]

Description of Additional Supplementary Files

File Name: Supplementary Data 1
Description: This file contains the MS-based quantification of DNMT1 interacting chromatin proteins.

File Name: Supplementary Data 2
Description: This file contains the list of identified ubiquitylated peptides in xDNMT1 pull-down.

File Name: Supplementary Data 3
Description: This file contains the list of identified phosphorylated peptides in xDNMT1 pull-down.
