# Supplementary material for: Two distinct modes of DNMT1 recruitment ensure stable maintenance DNA methylation
Source: Nat Commun. 2020 Mar 6;11:1222. doi: 10.1038/s41467-020-15006-4 (PMC7060239; doi:10.1038/s41467-020-15006-4)
Supplement: Supplementary file 2 — Peer Review [file 41467_2020_15006_MOESM2_ESM.pdf]

Editorial Note: Parts of this peer review file have been redacted as indicated to maintain the confidentiality of unpublished data.

Reviewers' comments:

Reviewer #1 (Remarks to the Author):

This paper by Nishiyama et al examines mechanisms of DNA methylation in eukaryotes.
Two proteins are known to be essential for DNA methylation maintenance in eukaryotes: the enzyme DNMT1, and the ubiquitin ligase UHRF1. Some of the molecular mechanisms have been elucidated. In particular, it has been shown that UHRF1 ubiquitinates H3. H3Ub is then bound by the ubiquitin-interaction motif (UIM) of DNMT1, activating the enzyme and promoting methylation maintenance. However, some gaps in knowledge persist, and this paper brings a new actor into the picture: PAF15. There are only 6 papers on PAF15, but it is known to play a role in the DNA damage response (Povlsen NCB 2012), and to be ubiquitinated by UHRF1 (Karg JMB 2017).

The findings in this new paper are the following:
1-PAF15Ub is found in chromatin during replication in a Xenopus in vitro replication system. In this system, it is modified on K18 and K27. Mutating the residues prevents both ubiquitination and incorporation into replicating chromatin.
2-The ubiquitination and loading of PAF15 requires UHRF1. Molecularly, the PHD domain of UHRF1 recognizes the histone-like N-terminus of PAF15, and a crystal structure for this is provided. Preventing the PHD-PAF15 interaction diminishes ubiquitination and chromatin loading (without eliminating them).
3-PAF15Ub binds the UIM of DNMT1, with high affinity and presumably by the same mechanism that H3Ub employs to bind the UIM.
4-There is some sort of balance between PAF15 and H3 ubiquitination: removing PAF15 from xenopus extracts leads to increased H3 ubiquitination. Kinetically, PAF15 ubiquitination seems to occur early in replication of the Xenopus system.
5-Mutation of the critical PAF15 residues in ES cells show that the mechanisms appear conserved in mouse. DNMT1 interacts with PAF15 only if it can be ubiquitinated. Cells with an ubiquitination-resistant PAF15 have decreased levels of DNA methylation. The regions that are affected tend to be early-replicating, consistent with previous findings.

The paper is written clearly. The experiments are well presented, adequately controlled, and correctly interpreted. The paper does a good job of combining in vitro replication data with structural analysis and mouse ES cell work. I think together the work provides an interesting advance for the field.

Most of the conclusions are very firmly supported by the data, and I am convinced that PAF15 is indeed involved in DNA methylation maintenance. One minor point that remains a little hazy is how this overlaps with previously discovered mechanisms, and especially H3 ubiquitination by UHRF1. I do not feel that the arguments saying that PAF15 acts in early replication, and H3 later, are extremely compelling. Maybe a different analysis of the RRBS data could make the point stronger, but I'm not sure.

There are also some minor gaps concerning the structure data of figure 2. For instance, the K4A mutant, which is only mildly impaired for chromatin recruitment (2d), is not tested in ITC. We would expect it to retain some interaction. Also, the proof that the PHD-PAF15 interaction is functionally important would be strengthened by showing that a PHD mutant of UHRF1 fails to cause PAF15Ub in the Xenopus replication assay.

Unless I am mistaken, the second row of Fig 5a shows GFP-mDNMT1, and not GFP-UHRF1 as labeled. It would be useful to remind the readers in the figure what the F3H approach is. Please explain more clearly what DNMT1-Trap is. Statistics are needed in Fig 5d. Please indicate the p-value for 5e directly in the figure, rounding it to 2 decimals.

Reviewer #2 (Remarks to the Author):

In this manuscript, Nakanishi and colleagues describe an interaction between DNMT1 and PAF15. They showed that PAF15 is important for DNMT1 recruitment to DNA using several orthogonal approaches and supported by convincing biochemical data.

The interaction between PAF15 and DNMT1 was identified using a mass spectrometry screen. Could the authors clarify whether the interaction between PAF15 and DNMT1 has been identified before or is it a novel interaction.

Only two peptides support PAF15 identification. Is it because there are only 6-7 “theoretical” tryptic peptides which could be generated from PAF15 protein sequence? could it be related to protein abundance or strength of the association. Intensity based MS data analysis could be very revealing for such a case in contrast to spectral counts based analysis.

The reader would benefit from more detailed description of MS data in the main text. Please note in the text how many proteins were identified in total in the MS analysis, what criteria was used to distinguish proteins binding in a UbVS+Ub dependent manner and how many proteins were found to bind in a UbVS+Ub dependent manner.

Please deposit the raw data and search results in a public repository such as PRIDE.

Please describe what it is shown in columns C to N in a supplementary table.

In a supplementary table please provide more information from search engine results. If it is present in the output of PD 1.3 please provide a number of unique peptides identified, an identification quality score, the sequence coverage and the number of spectral counts for every protein.

In the method description it is noted that the raw data was searched for phosphorylated and di-Gly modified peptides. Could you please comment on how many of modified peptides were identified and whether you identified any modified peptides dependent on UbVS+Ub treatment.

It is generally accepted in proteomics community to present protein enrichment in a volcano plot. Please see Figure 4 in a recent publication in Nature Communications (DOI: 10.1038/s41467-018-04619-5, Papachristou et. al 2018). A Volcano plot well summarizes protein enrichment data in terms of how many total proteins were identified and what fraction was differentially regulated between the conditions. In addition, it provides a good perspective on ranking of strong interactors. However, it is important to note that proteins may not appear significantly enriched in a volcano plot due to transient nature of the interaction or protein instability during the sample preparation. I am convinced that the main findings of the manuscript are clear without a volcano plot, however I would recommend to add it. There are many software packages one can use to generate a volcano plot. In case you would like to use a user friendly solution here is a recommendation: http://www.coxdocs.org/doku.php?id=perseus:user:use_cases:interactions.

Reviewer #3 (Remarks to the Author):

The authors have accumulated a very large body of data, but individual experiments are often poorly documented and described superficially. Therefore, it is very difficult to assess the scientific value of this work in the present form. However, I have some doubts whether key claims are backed up by data and key findings are sufficiently supported. Overall, I have the impression that

this work is better presented in two or three focused manuscripts in biochemical journals.

Detailed comments
1) Key claims are not backed up by data. This point is illustrated by inspecting the last sentences of the abstract:
PAF15Ub2 … controls the recruitment of DNMT1 in a DNA replication-coupled manner-not shown
Loss of PAF15Ub2 results in impaired DNA methylation at sites replicating during early S phase-not shown
In contrast, outside of S phase or when PAF15 ubiquitylation is perturbed, UHRF1 ubiquitylates histone H3 to promote DNMT1 recruitment-changes in H3 ubiquitination are shown, but it is not shown they are caused by UHRF1
Together, we identify replication-coupled and uncoupled mechanisms of maintenance DNA methylation, both of which collaboratively ensure the stable DNA methylation – not shown
2) Fig. 1A: It is necessary to document statistics and p-values. Finding one or two peptides of PAF15 is not very impressive. Based on this picture it is difficult to understand why the authors did focus on PAF15.
3) All IP need to be documented better. Protein sizes must be indicated. In most cases it is unclear which antibody was used (e.g. in 1B upper panel, was it xPAF15, His, Ub, something else). Similar problems appear with all images. Also specific Ub product need to be validated by two separate western blots against the protein and Ub.
All IP reaction are based on chromatin extracts. Controls are always needed showing the loading. In cases where recombinant proteins are added, input controls are also needed.
4) In Fig. 1B and several following: Explain why there is a decline in signal at later time points.
5) Line 162 (similar claims are made at other places) "These results 162 demonstrate that the dual mono-ubiquitylation of xPAF15 at K18 and K27, is required for xPAF15 to efficiently bind chromatin" – This is not shown. To make this claim the authors need to study monoubiquitylated and show that they both do not bind, but di-Ub does.
6) The data in Fig. 2 are nice and a highlight of the work. Still questions appear. Why is there a band at xDNMT1 in 2A in the Delta DNMT1 sample? D is lacking pulldown control. In F the R2A effect is not clear. Experiments in d-f should be repeated, appropriately quantified and also reported as averages with error bar.
7) Questions regarding Fig. 3: The pulldown of UHRF1 with DNMT1 in 3A is very faint. This should be compared with literature findings. What is shown in the FLAG panel in 3B? The ITC curve in 3C is not convincing. Additional titrations with smaller injections should be conducted. Line 233: QC of the synthetic peptide should be shown (Gel, mass spec, etc). Line 237: Again it is not shown here that Di-Ub is needed. The two mono-Ub peptides need to be prepared and investigated to make this claim.
8) Line 243-248 and Fig. 3e: This part is not convincing and should be removed.
9) Fig. 3D: Comparisons with unbound RFTS must be made and shown. This experiment provides only weak evidence. Why is this low resolution data shown, if the structure is solved?
10) Paragraph starting with line 258. Pulse chase experiments need to be conducted to make this kind of statements. These experiments are necessary also given the claims listed in the abstract. The complicated kinetics in 4D need to be explained better and more controls are needed. Fig. 4C and D are lacking input controls. It needs to be shown that the H3 ubiquitylation is caused by UHRF1.
11) The mouse data constitute a strong break. Methods are different and the approach is only partially overlapping. I see some value in these data but they are preliminary and need to be further developed. The co-localisation in 5A should be validated by FRET or PLA. The pulldown of PAF15 in 5B is very weak and again no input controls are shown. The RRBS data must be presented in much more details, documenting read depth, statistics of DMRs. The expression levels of critical factors like DNMT1 in the cell lines used in Fig. 5 must be validated and shown.

Point-by-point responses

For reviewer 1

1. One minor point that remains a little hazy is how this overlaps with previously discovered mechanisms, and especially H3 ubiquitination by UHRF1. I do not feel that the arguments saying that PAF15 acts in early replication, and H3 later, are extremely compelling. Maybe a different analysis of the RRBS data could make the point stronger, but I'm not sure.

This point is important and well taken. To better support our conclusion that PAF15Ub2 plays a role in maintaining DNA methylation during early replication, we identified chromatin sites showing changes in methylation in the absence of UHRF1 or DNMT1 and examined whether they are correlated with replication timing. Although we found that hypomethylated sites in the absence of PAF15Ub2 are correlated with early replication timing, those in the absence of UHRF1 or DNMT1 were no longer correlated with any replication timing. These results suggest that DNMT1 recruitment is mediated by functionally distinct pathways dependent on the replication timing. Thus, we revised the Abstract section as follows: "Together, our results suggest that there are two distinct mechanisms underlying replication timing-dependent recruitment of DNMT1 through PAF15Ub2 and H3Ub2, both of which are prerequisite for high fidelity DNA methylation inheritance.".

2. There are also some minor gaps concerning the structure data of figure 2. For instance, the K4A mutant, which is only mildly impaired for chromatin recruitment (2d), is not tested in ITC. We would expect it to retain some interaction.

This point is well taken. According to the suggestion, we repeated the ITC experiment with K4A (now K5A). Indeed, the K5A mutant retained some interaction with hPHD. The results are shown in the revised Fig. 2b and described in the revised text (page 10, lines 208-209).

3. Also, the proof that the PHD-PAF15 interaction is functionally important would be strengthened by showing that a PHD mutant of UHRF1 fails to cause PAF15Ub in the Xenopus replication assay.

To address this concern, we analyzed PAF15 ubiquitylation and DNMT1 chromatin recruitment during S phase in UHRF1-depleted extracts supplemented with purified recombinant xUHRF1-Flag3-WT or its PHD mutant harboring D333A/D336A. This experiment clearly showed that both UHRF1-dependent PAF15 ubiquitylation and DNMT1 chromatin recruitment require functional PHD. The results are shown in the revised Fig. 2a and described in the revised text (pages 8-9, lines 169-185).

4. Unless I am mistaken, the second row of Fig 5a shows GFP-mDNMT1, and not GFP-UHRF1 as labeled.

We corrected the labeling in the revised Figure 5f.

5. It would be useful to remind the readers in the figure what the F3H approach is.

We added a detailed description of the principle of the F3H assay in the main text (page 15, lines 344-348) as well as a schematic representation of the F3H approach in the revised Fig. 5e.

6. Please explain more clearly what DNMT1-Trap is.

DNMT1-Trap is the commercial name for a resin consisting of a high affinity anti-DNMT1 nanobody coupled to agarose beads used for immunoprecipitation of DNMT1. We have now changed the term "DNMT1-Trap" to "DNMT1 nanobody" in the revised Fig. 5e and in its respective figure legend. In addition, we provide a description of the DNMT1-Trap in revised Materials and Methods section (page 40, lines 1033-1036).

7. Statistics are needed in Fig 5d.

We have now included p-values in all RRBS comparison plots (see revised Figs. 6a and 6b).

8. Please indicate the p-value for 5e directly in the figure, rounding it to 2 decimals.

According to the reviewer's comment, we add the p-value for the results shown in the revised Figs. 6d-f (the previous Fig. 5e).

For reviewer 2

1.The interaction between PAF15 and DNMT1 was identified using a mass spectrometry screen. Could the authors clarify whether the interaction between PAF15 and DNMT1 has been identified before or is it a novel interaction.

In Emanuele et al (2011), the authors performed affinity purification of HA-tagged PAF15 from HEK-293T cells and identified DNMT1 as one of the co-purified proteins. However, to our knowledge, PAF15 was not detected as an interactor in the inverse experiment with DNMT1 affinity purification. Moreover, our finding suggests that PAF15-DNMT1 interaction is ubiquitylation-dependent. We mention this point in the revised text (page 6, lines 124-125).

2. Only two peptides support PAF15 identification. Is it because there are only 6-7 "theoretical" tryptic peptides which could be generated from PAF15 protein sequence? could it be related to protein abundance or strength of the association. Intensity based MS data analysis could be very revealing for such a case in contrast to spectral counts based analysis.

This point is very important and well taken. As suggested by the reviewer, we now show ubiquitylation-dependent PAF15 enrichment in xDNMT1 pull-down using a volcano plot based on label-free quantification with the newer version of software (Proteome Discoverer 2.2). In agreement with our previous reports, this plot indicated that DNMT1 specifically binds to various histone proteins,

presumably due to a complex formation of these histones with ubiquitylated histone H3. Furthermore, this analysis did indeed reveal that PAF15 was significantly enriched in xDNMT1 pull-down depending on the addition of ubiquitin to UbVS-treated extracts. The results are shown in the revised Fig. 1a and discussed in the text (pages 6, lines 113-127).

3. The reader would benefit from more detailed description of MS data in the main text. Please note in the text how many proteins were identified in total in the MS analysis, what criteria was used to distinguish proteins binding in a UbVS+Ub dependent manner and how many proteins were found to bind in a UbVS+Ub dependent manner.

This point is important. In the MS analysis, we identified 2840 unique peptides (including 26 ubiquitylated and 17 phosphorylated peptides) that mapped to 303 proteins in chromatin lysates from UbVS-treated extracts in the presence (UbVS+Ub) or absence (UbVS) of free ubiquitin (revised Supplementary Table 1). Of these, 24 proteins were highly enriched in the DNMT1 pull-downs depending on the addition of ubiquitin to UbVS-treated extracts (log_2fold-change>2, p-value<0.05) (revised Fig. 1a, revised Supplementary Table 1). We also found an enrichment of 8 ubiquitylated and 2 phosphorylated peptides in the data set (the revised Supplementary Tables 2 and 3). We describe this information in the revised Results section (pages 6, lines 113-127).

4. Please deposit the raw data and search results in a public repository such as PRIDE.

We have deposited our data set including the raw data and search results at the ProteomeXchange Consortium via the PRIDE partner repository (dataset identifier “PXD015282").

5. Please describe what it is shown in columns C to N in a supplementary table. In a supplementary table please provide more information from search engine results. If it is present in the output of PD 1.3 please provide a number

of unique peptides identified, an identification quality score, the sequence coverage and the number of spectral counts for every protein.
We describe this information in the revised Supplementary Table 1.

6. In the method description it is noted that the raw data was searched for phosphorylated and di-Gly modified peptides. Could you please comment on how many of modified peptides were identified and whether you identified any modified peptides dependent on UbVS+Ub treatment.
According to the reviewer's comment, we describe this information in the revised text (pages 6, lines 113-127) and the revised Supplementary Tables 2 and 3.

7. It is generally accepted in proteomics community to present protein enrichment in a volcano plot. Please see Figure 4 in a recent publication in Nature Communications (DOI: 10.1038/s41467-018-04619-5, Papachristou et. al 2018). A Volcano plot well summarizes protein enrichment data in terms of how many total proteins were identified and what fraction was differentially regulated between the conditions. In addition, it provides a good perspective on ranking of strong interactors. However, it is important to note that proteins may not appear significantly enriched in a volcano plot due to transient nature of the interaction or protein instability during the sample preparation. I am convinced that the main findings of the manuscript are clear without a volcano plot, however I would recommend to add it. There are many software packages one can use to generate a volcano plot. In case you would like to use a user friendly solution here is a recommendation:
http://www.coxdocs.org/doku.php?id=perseus:user:use_cases:interactions.
According to the suggestion, we show protein enrichment data as a volcano plot based on label-free quantification.

For reviewer 3
1. Key claims are not backed up by data. This point is illustrated by inspecting the last sentences of the abstract:

PAF15Ub2 … controls the recruitment of DNMT1 in a DNA replication-coupled manner-not shown

In the previous Fig. 1c (revised Fig. 1c), we showed that PAF15Ub2 and DNMT1 chromatin loading were replication-dependent. The role of PAF15Ub2 in the DNMT1 chromatin loading was also shown in the previous Figs. 3 and 4 (revised Figs. 3 and 4).

2. Loss of PAF15Ub2 results in impaired DNA methylation at sites replicating during early S phase-not shown

In the previous Fig. 5e (revised Fig. 6d), we showed that the substitution of amino acids necessary for PAF15Ub2 resulted in DNA hypomethylation at early replicating domains.

3. In contrast, outside of S phase or when PAF15 ubiquitylation is perturbed, UHRF1 ubiquitylates histone H3 to promote DNMT1 recruitment-changes in H3 ubiquitination are shown, but it is not shown they are caused by UHRF1

We showed that both PAF15 and H3 ubiquitylation were dependent on the presence of UHRF1 in the previous Figs. 2a, and 4d (revised Fig.2a, revised Supplementary Figs. 2a-c, and revised Fig. 4g) as well as in our past works (Nishiyama et al Nature 2013, Ishiyama et al. Mol Cell 2017). In the revised version, we describe the additional experiments performed in order to further confirm the role of UHRF1 in the ubiquitylation of histone H3. We depleted UHRF1 from egg extracts and added purified recombinant xUHRF1 90 min after the start of DNA replication. Addition of xUHRF1-WT induced efficient histone H3 ubiquitylation whereas that of the xUHRF1-D333A/D336A mutant failed to do so. These results suggest that histone H3 ubiquitylation is indeed mediated by UHRF1. The results are shown in the revised Supplementary Fig. 4f and described in the revised text (page 14, lines 307-308).

4. Together, we identify replication-coupled and uncoupled mechanisms of maintenance DNA methylation, both of which collaboratively ensure the stable DNA methylation – not shown

This point is very important and thus well taken. The depletion of xPAF15 from egg extracts showed only a partial loss of DNA methylation. The level of xH3Ub2 on chromatin as well as in the complex with xDNMT1 increased upon xPAF15 depletion and the masking of H3Ub2 by RFTS in the absence of PAF15 resulted in an almost complete loss of DNA methylation. These observations suggest that loss of PAF15 function in the recruitment of DNMT1 could be rescued by histone H3, ensuring the stable inheritance of DNA methylation. We also showed that UHRF1 efficiently ubiquitylated PAF15 during early S-phase but preferred histone H3 as its substrate in late S-phase. The results are shown in the revised Fig. 4 and the revised Supplementary Figs. 4d-4f and discussed in the revised text (pages 12-14).

5. Fig. 1A: It is necessary to document statistics and p-values. Finding one or two peptides of PAF15 is not very impressive. Based on this picture it is difficult to understand why the authors did focus on PAF15.

This point is almost the same as that of reviewer 2 (comment 2). Please see our above response.

6. All IP need to be documented better. Protein sizes must be indicated. In most cases it is unclear which antibody was used (e.g. in 1B upper panel, was it xPAF15, His, Ub, something else). Similar problems appear with all images.

This point is important. According to the reviewer's indication, we have now included protein sizes, and the antibodies used in all images.

7. Also specific Ub product need to be validated by two separate western blots against the protein and Ub.

This point is well taken. We performed two separate western blots against the immunoprecipitated PAF15Ub2 and PAF15Ub1 from chromatin lysates and found that PAF15 bands were recognized by anti-ubiquitin antibody. Taken together with results showing an upshift of PAF15Ub2 with the addition of His_6-tagged ubiquitin (the revised Fig. 1b), these results suggest that PAF15

undergoes ubiquitylation. The results are shown in the revised Supplementary Fig. 3a and discussed in the text (page 10, lines 217-219).

8. All IP reaction are based on chromatin extracts. Controls are always needed showing the loading. In cases where recombinant proteins are added, input controls are also needed.

According to the reviewer's indication, in all the revised chromatin binding experiments, ORC2 or histone H3 are shown as a loading control for chromatin samples. Input controls are also shown in the revised Figs. 1b (see Supplementary Fig.1f), 1d, 1e, 2a (see Supplementary Fig. 2a), 2d, 3b, 4b (see Supplementary Fig. 4b), 4c, 4g (see Supplementary Fig. 4e), 5d, and 5e, respectively.

9. In Fig. 1B and several following: Explain why there is a decline in signal at later time points.

Upon the addition of sperm chromatin to *Xenopus* interphase egg extracts, replication-competent nuclei are assembled and a single round of DNA replication occurs. Under these conditions, DNA replication typically starts approximately 40 min after the sperm addition. After completion of DNA replication, most factors involved in chromosomal replication, including DNMT1, and UHRF1, are released from chromatin. We have incorporated this explanation into the revised text (page 7, lines 130-135).

10. Line 162 (similar claims are made at other places) "These results demonstrate that the dual mono-ubiquitylation of xPAF15 at K18 and K27, is required for xPAF15 to efficiently bind chromatin" – This is not shown. To make this claim the authors need to study monoubiquitylated and show that they both do not bind, but di-Ub does.

This is important and well taken. During the revision of our manuscript, we found that the chromatin binding of in-vitro translated recombinant xPAF15 is highly variable among the extracts used. Therefore, in place of this, we repeated the experiments using recombinant xPAF15-WT, K18R, K27R, or K18RK27R

(KRKR) purified from insect cells. These proteins were added to interphase extracts depleted of xPAF15 at the same level as endogenous PAF15 (~300 nM). Although we found that most xPAF15-WT bound to chromatin as a dual mono-ubiquitylated form, xPAF15-K18R and K27R underwent single mono-ubiquitylation, and also efficiently bound to chromatin. These results suggest that single mono-ubiquitylation of xPAF15 at K18 and/or K27 is sufficient for its chromatin loading. We mentioned these points in the revised manuscript (pages 7-8, lines 144-154).

11. The data in Fig. 2 are nice and a highlight of the work. Still questions appear. Why is there a band at xDNMT1 in 2A in the Delta DNMT1 sample?
In *Xenopus* egg extracts, immunodepletion of xDNMT1 using its specific antibodies could not reach 100 %, meaning that a very low level of xDNMT1 still remained in the depleted extracts. Depletion of the majority of xDNMT1 resulted in a remarkable accumulation of UHRF1 on the chromatin, which in turn increased levels of PAF15Ub2 and H3Ub2 on the chromatin and revealed the faint band of xDNMT1. Nevertheless, we performed an additional experiment to show that PAF15 ubiquitylation was enhanced in xDNMT1-depleted extracts. The results are shown in revised Supplementary Figs. 2b and c and discussed in the text (page 9, lines 180-184).

12. D is lacking pulldown control.

According to the reviewer's indication, we repeated the experiment with histone H3 as a loading control for chromatin samples.

13. In F the R2A effect is not clear. Experiments in d-f should be repeated, appropriately quantified and also reported as averages with error bar.
According to the reviewer's comment, we repeated the experiments shown in the previous Figs. 4d-f and the results with error bars are shown in the revised Figs. 4d-f.

14. Questions regarding Fig. 3: The pulldown of UHRF1 with DNMT1 in 3A is very faint. This should be compared with literature findings.

This point was extensively analyzed in our previous works (Nishiyama et al., Nature 2013, Ishiyama et al., Mol Cell 2017). Although direct interaction between UHRF1 and DNMT1 has been proposed as a means of recruiting DNMT1 to chromatin, we failed to detect a stable interaction between UHRF1 and DNMT1 both in egg extracts and on chromatin.

15. What is shown in the FLAG panel in 3B?

We addressed this point in the revised Fig. 3b.

16. The ITC curve in 3C is not convincing. Additional titrations with smaller injections should be conducted. Line 233: QC of the synthetic peptide should be shown (Gel, mass spec, etc).

According to the comment, we now provide a gel image of mono- and dual monoubiquitinated PAF15 in Supplementary Fig. 3c in order to demonstrate the quality of the proteins. Although we also performed the ITC measurements at lower concentrations of dual-monoubiquitinated PAF15 (syringe: 62.5 μM) and hRFTS (cell: 5 μM), we could not obtain the correct heat derived from the interaction. However, our current ITC data clearly show high binding affinity and a 1:1 stoichiometric interaction between hRFTS and dual monoubiquitinated PAF15, which provides convincing evidence that dual monoubiquitination of PAF15 is required for the specific interaction with the DNMT1 RFTS domain.

17. Line 237: Again it is not shown here that Di-Ub is needed. The two mono-Ub peptides need to be prepared and investigated to make this claim.

According to the reviewer's comments, we performed ITC measurements using K15 or K24 monouniquitinated PAF15 peptides. The purity of the ubiquitinated PAF15 peptides is shown in the revised Supplementary Fig. 3c. The results are

shown in the revised Figure 3c.

18. Line 243-248 and Fig. 3e: This part is not convincing and should be removed.

We have attempted crystallization of the hRFTS bound to dual monoubiquitinated PAF15, but failed in our efforts. Instead of high-resolution structural evidence, we succeeded in obtaining good quality SAXS data on the complex. The SAXS data is indeed low resolution, however, the advantage of this method is that the overall shape and structural change of the macromolecule is obtained without crystallization. The method of ab initio model construction from the SAXS data was established by the Svergun group (D. I. Svergun. Restoring low resolution structure of biological macromolecules from solution scattering using simulated annealing. Biophys J. 2879-2886. 1999) and is useful for obtaining structural information on macromolecules. In this study, we were able to show the structural commonality between hRFTS:PAF15Ub2 and hRFTS:H3Ub2; the profile of the distance distribution function and the bead models show that the overall shapes closely resemble each other. Thus, we demonstrate that two ubiquitins on PAF15 are bound to a ubiquitin-binding module of hRFTS in a manner similar to that of dual mono-ubiquitylated H3 seen in the crystal structure, which shows the molecular mechanism of DNMT1 recruitment to replication sites by dual mono-ubiquitylated PAF15. According to the reviewer's suggestion, we have now moved these panels from revised Fig. 3 to Supplementary Figs.3e and f.

19. Fig. 3D: Comparisons with unbound RFTS must be made and shown. This experiment provides only weak evidence. Why is this low resolution data shown, if the structure is solved?

We added SEC-SAXS data on apo-hRFTS in the revised Supplementary Figs. 3d-f. Supplementary Fig. 3f shows that the ab initio model of hRFTS derived from the SAXS data was not well superimposed on the crystal structure of apo-hRFTS (PDB ID: 3EPZ), which indicates that the relative spatial arrangement of the two-lobe structure in hRFTS is flexible in solution, however,

the binding of dual mono-ubiquitinated PAF15 to hRFTS leads to conformational stabilization of hRFTS. PAF15 is known to be an intrinsically disordered protein, thus two ubiquitins on the PAF15 seem to not interact with each other because of the flexibility of the PAF15. Recently, during our revision process, another group reported that the ubiquitins on PAF15 form transient dimers due to a high local concentration, which was analyzed using a SAXS-derived conformational ensemble of dual mono-ubiquitinated PAF15 (Gonzalez et al., ACS Chem Biol 2019). We refer the structural evidence in our manuscript.

20. Paragraph starting with line 258. Pulse chase experiments need to be conducted to make this kind of statements. These experiments are necessary also given the claims listed in the abstract. The complicated kinetics in 4D need to be explained better and more controls are needed. Fig. 4C and D are lacking input controls.

This point is very important and well taken. We repeated the experiments shown in the previous Fig. 4d with a slight modification as follows. At various times after the initiation of DNA replication, purified recombinant xUHRF1 was added to the UHRF1-depleted extracts and ubiquitylation of PAF15 and H3 was examined. UHRF1 added to UHRF1-depleted extracts before replication initiation (A; t=0 min) failed to ubiquitylate either PAF15 or H3. When UHRF1 was added to the reaction during early S phase (B or C; t=30 or 60 min), UHRF1 specifically ubiquitylated PAF15, but not H3. Importantly, UHRF1 added during late S phase (D-F; t=90-150 min) predominantly ubiquitylated H3 but not PAF15. These data indicate that UHRF1 on the replicating chromosome during early S phase tends to ubiquitylate PAF15, but during middle to late S phase preferentially ubiquitylates H3, supporting our conclusion that DNMT1 relies on two functionally distinct pathways, comprising PAF15Ub2 and H3Ub2, to ensure a high fidelity of maintenance DNA methylation during S phase. These results are shown in the revised Figs. 4f-g and the revised Supplementary Figs. 4d-f and are discussed in the text (pages 13-14, lines 296-313). We show input controls in the revised Fig. 4c and Supplementary Fig. 4e (for revised Fig. 4g).

21. It needs to be shown that the H3 ubiquitylation is caused by UHRF1.
Please see above response (comment 3 to reviewer 3).

22. The co-localisation in 5A should be validated by FRET or PLA.
Since we agree with the reviewer that it would be interesting to study the molecular dynamics of the PAF15-DNMT1 complex in vivo, we tried to perform FRET and PLA measurements using fluorescent proteins conjugated to DNMT1 and PAF15. Unfortunately, the experiments did not work well due to a lack of specialized instrumentation for nanosecond fluorescence lifetime measurements and failure in generating high quality primary antibodies to DNMT1 and PAF15. We believe that the F3H assay constitutes an established means for assessing whether proteins interact in vivo[1], and this assay has often specifically been employed to provide in vivo validation of protein-protein interactions determined from in vitro biochemical and structural biology data[2-4]. As such, we feel that the application of the F3H assay in this study is an entirely appropriate means of demonstrating not only the existence of an in vivo PAF15 and DNMT1 complex but also the dependence of this complex on K15 and K24 residues within PAF15.

Nevertheless, we appreciated the reviewer's request for additional validation of the mPAF15-mDNMT1 interaction. As all DNA methylation analyses were performed with our set of endogenous PAF15 ubiquitylation mutant ESCs, we focused our experimental efforts on validating the endogenous mPAF15-DNMT1 interaction in ESCs. Importantly, our revised manuscript now includes additional biochemical evidence demonstrating the ability of endogenous mDNMT1 to specifically recognize mPAF15Ub2 in mouse ESCs (discussed in depth in the response below; see revised Figs. 5a-c).

References

1. Herce, H. D., Deng, W., Helma, J., Leonhardt, H. & Cardoso, M. C. Visualization and targeted disruption of protein interactions in living cells. *Nat Commun* 4, 2660 (2013).
2. Borgel, J. *et al.* KDM2A integrates DNA and histone modification signals through a CXXC/PHD module and direct interaction with HP1. *Nucleic Acids Research* 45, 1114–1129 (2017).

3. Ferry, L. *et al.* Methylation of DNA ligase 1 by G9a/GLP recruits UHRF1 to replicating DNA and regulates DNA methylation. *Molecular Cell* 67, 550–565.e5 (2017).
4. Kori, S. *et al.* Structure of the UHRF1 tandem tudor domain bound to a methylated non-histone protein, LIG1, reveals rules for binding and regulation. *Structure* 27, 485–496.e7 (2018).

23. The pulldown of PAF15 in 5B is very weak and again no input controls are shown.

We performed a reciprocal immunoprecipitation using the anti-PAF15 antibody in wild-type and PAF15 KRKR mouse ESC nuclear extracts. Wild-type PAF15 underwent efficient ubiquitylation and co-immunoprecipitated DNMT1, but PAF15 KRKR failed to do so. Taken together with the result showing ubiquitylated PAF15 in DNMT1 immunoprecipitates in the revised Fig. 5a, these results suggest the essential role of PAF15Ub2 in its interaction with DNMT1 in mouse ESC cells. The results including input controls are shown in the revised Figs. 5b-d and described in the revised text (pages 14-15, lines 316-342).

24. The RRBS data must be presented in much more details, documenting read depth, statistics of DMRs.

According to the reviewer's indication, we have now provided the number of total reads as well as the number and percentage of mapped reads for each RRBS sample in revised Supplementary Table 6. With respect to the statistics of differentially methylated regions, we included the information in the revised figure legend for revised Fig. 6 (page 29, lines 768-771), in place of the Methods section.

25. The expression levels of critical factors like DNMT1 in the cell lines used in Fig. 5 must be validated and shown.

According to the reviewer's indication, we examined the expression levels of *Dnmt1* as well as *Uhrf1* and *Paf15* in wt, UHRF1 KO, DNMT1 KO, PAF15 K15R, PAF15 K24R, and PAF15 KRKR ESCs using qRT-PCR (revised Supplementary Fig. 5g). Although we observed a slight upregulation of *Dnmt1* and *Uhrf1* transcripts in *Paf15* mutant ESCs compared with those of wt ESCs, these differences were not statistically significant. Furthermore, we have now provided immunoblots of endogenous DNMT1 using whole cell lysates from these cell lines (revised Figs. 5a-c, Input), showing no obvious change in DNMT1 protein abundance as a result of PAF15 mutations.

Reviewers' comments:

Reviewer #1 (Remarks to the Author):

The authors have made an earnest effort at addressing my questions. I am satisfied with their answers and have no further requests.

Reviewer #2 (Remarks to the Author):

The authors did an excellent job in revising the manuscript. The figure 1a looks very convincing. A minor detail is that one of the points is labeled as poly-ubiquitin. I am not sure if it is possible from a dataset based on tryptic peptides to confidently identified poly-ubiquitin. Please double check whether it is ubiquitin or poly-ubiquitin.

Reviewer #3 (Remarks to the Author):

The authors have collected an enormous body of data showing over 60 complex data panels. I appreciate that essential technical controls which were missing almost completely in the previous version of the manuscript have now been added. However, instead of being advantageous its large amount of data is the biggest weakness of the manuscript. The authors simply want too much for one publication. They collected too many experiments and want to make too many claims. It is simply impossible to describe all these experiments and discuss their results sufficiently in the context of one publication. Therefore, some of the experiments are described superficially, important information is not given and claims are too general for the data provided. Moreover many of the key claim were just documented with one experiment, where confirmation of the interpretation by a second or third experiment would have been needed. In the following I have collected some of the questions intimately connected with the presented data. I believe that the authors have an interesting and relevant set of data, but they should spend more care on the details instead of meshing all together, split the paper into 2 or 3 which then can make defined and carefully written claims that are backed up by data.
The data presented in Fig. 2 are very nice, convincing and would deserve their own paper, perhaps after showing some biological effects of R3A or T4D. It is unfortunate that due to the unfocused composition of this paper these data are not covered by the title of the manuscript and they are not mentioned in the abstract. This gives a high risk that they will be simply ignored by most other researchers.
The data shown in Fig. 5 and 6 are not focusing on chromatin interaction and use different model systems. They are only weakly connected to the rest of the paper and after appropriate revision they may be better presented as an independent paper.

Comments:
1. Introduction, p. 5: the points 2 and 3 listed here are not related to the present work.
2. p. 6, line 109: The reference to S1a and b is wrong. A figure would be needed that shows the purified protein.
3. Fig. S1b: The figure legend is not informative.
4. Fig. 1C: The relevance of SDS is not explained.
5. p. 7, first paragraph: References for the claims in the first sentenced are missing.
6. p. 7: The experimental system of studying DNA replication after addition of sperm chromatin is very special and non-physiological. It needs to be shown that reactions and interaction observed in this system do occur under normal conditions.
7. p. 7 and Fig. 1b: Contrary to what is stated in the manuscript ubiquitylation levels are not increased after addition of His-Ub. The authors imply that HisUb and Ub lead to a difference in the migration of the ubiquitinated PAF15. This should be confirmed.

8. p. 7 and Fig. 1D: Why is the ubiquitylation of wt PAF15 so weak? Why is there stronger ubiquitylation of the single mutants? Can it be excluded that the double mutant protein is misfolded and therefore lacks ubiquitylation?
9. Fig. 1g: PAF15 input is missing. I do not get why inhibition of deubiquitination should lead to release of PAF15 from chromatin. Indirect effects of UbVs need to be excluded.
10. Fig. 1f and p. 8: To make the claim of a stable complex formation a reciprocal pulldown would be needed as confirmation.
11. Fig. 1i and 1e: Can it be excluded that the FF mutant is misfolded and therefore fails to interact?
12. Fig. 1e and p. 8: It should be noted that the KR mutant also fails to bind to chromatin.
13. Fig. 3b: PCNA input is missing.
14. P. 11: "we prepared a single or mono-ubiquitylated hPAF15" – unclear
15. Fig. 3c: data should be presented together with error margins based on independent repeats of the experiment. The Kd of 1.4 nM +-0.7 for Ub2 listed on p. 11 line 233 cannot be determined in this setting, because it is covered by only one data point in the titration. Experiments must be repeated at lower protein concentrations or using a different method. The justification for using a two site fit in the case of K24Ub must be given.
16. p. 11, line 235: Contrary to what is stated in the text, the high binding constant of 3 nM of the K24Ub construct is not far away from the Ub2 construct.
17. p. 11 and Suppl. Fig. 3d: The gel filtration profiles should be shown to document that complex formation occurred.
18. p. 11-12: The interpretation of the low resolution SAXC data is not convincing and the conclusions at the end of p.11 and beginning of p. 12 are overinterpreted.
19. There is no reference to Fig. 3d in the paper.
20. Related to the response of the authors that chromatin binding of in-vitro translated recombinant xPAF15 is not reproducible, the figure panels reporting this type of data should be removed.
21. Fig. 4a and d: indicate what the error bars are showing. Specify the number of experiments. Provide p-values.
22. p. 13: The concept of the experiments adding hRFTS to the extracts is not convincing. This is prone to numerous side reactions and indirect effects.
23. Fig. 4g is interesting. Is this result reproducible?
24. The PAF15 pulldown in Fig. 5a and b is very weak. The PAF15 input is missing in both panels. It is needed to support the conclusions in the text. These are important data that should be improved.
25. Fig. 5c lacks the necessary input controls.
26. The F3H assay should be based on more than 20 cells. Experiments should be repeated independently and the results of the independent experiments documented separately not combined. These studies should be done blinded for the experimenter.
27. Fig. S6a: data are semiquantitative. LC-MS should be done.
28. For the RRBS statistics about the coverage and read depth of CpG sites should be provided. Suppl. Table S6 suggests that the read depth per CpG site is not very high and perhaps not sufficient for detailed conclusions. This should include the distribution of the covered CpG sites over genomic elements for the different samples to exclude sampling artefacts.

Point-by-point responses

Response to the Editor's concern

specifically, we would expect you to address all the technical concerns of the referee, such as important points on missing controls and need for clarifications.

We believe that this point is related to the criticisms of Reviewer 3. Please see the responses to this issue shown below.

For Reviewer 2

The authors did an excellent job in revising the manuscript. The figure 1a looks very convincing. A minor detail is that one of the points is labeled as poly-ubiquitin. I am not sure if it is possible from a dataset based on tryptic peptides to confidently identified poly-ubiquitin. Please double check whether it is ubiquitin or poly-ubiquitin.

As pointed out by the reviewer 2, poly-Ub is the protein name registered in Uniprot (Swissprot) and was not identified and quantified at the protein level. We corrected poly-ubiquitin to ubiquitin in the revised version.

For Reviewer 3

The authors have collected an enormous body of data showing over 60 complex data panels. I appreciate that essential technical controls which were missing almost completely in the previous version of the manuscript have now been added. However, instead of being advantageous its large amount of data is the biggest weakness of the manuscript. The authors simply want too much for one publication. They collected too many experiments and want to make too many claims. It is simply impossible to describe all these experiments and discuss their results sufficiently in the context of one publication. Therefore, some of the experiments are described superficially, important information is not given and claims are too general for the data provided. Moreover many of the key claim were just documented with one experiment, where confirmation of the interpretation by a second or third experiment would have been needed. In the following I have collected some of the questions intimately connected with the presented data. I believe that the authors have an interesting and relevant set of data, but they should spend more care on the details instead of meshing all together, split the paper into 2 or 3 which then can make defined and carefully written claims that are backed up by data.

The data presented in Fig. 2 are very nice, convincing and would deserve their own paper, perhaps after showing some biological effects of R3A or T4D. It is unfortunate that due

to the unfocused composition of this paper these data are not covered by the title of the manuscript and they are not mentioned in the abstract. This gives a high risk that they will be simply ignored by most other researchers.

The data shown in Fig. 5 and 6 are not focusing on chromatin interaction and use different model systems. They are only weakly connected to the rest of the paper and after appropriate revision they may be better presented as an independent paper.

We believe that all the data in the revised version are required in order to draw a simple and clear conclusion that PAF15 as well as histone H3 serve as a platform for the recruitment of DNMT1 to hemi-methylated sites, both of which are prerequisite for high fidelity of DNA methylation maintenance through distinct replication timing-dependent modes. We first identified PAF15 as a ubiquitin-dependent DNMT1 binding protein in *Xenopus* egg extracts (Figure 1). Then, we showed that dual mono-ubiquitylation of PAF15 was catalyzed by UHRF1 with a similar mode for histone H3 (Figure 2). DNMT1 also bound to dual mono-ubiquitylated PAF15 in a similar mode for dual mono-ubiquitylated histone H3 (Figure3). We then tried to clarify the distinct role of PAF15 and histone H3 on DNMT1 recruitment during maintenance DNA methylation in *Xenopus* system (Figure 4). We generalized these observations in different species such as mouse cells (Figures 5 and 6).

In summary, we assembled a comprehensive study that clearly shows the role of PAF15 in the maintenance of DNA methylation with consistent and complementary data from two organisms, *Xenopus* and mice. Our results also place this new mechanism into a broader context. We show that DNMT1 and UHRF1 maintain DNA methylation via two distinct and non-redundant pathways; while PAF15Ub2 preferentially serves early replicating DNA, histone H3Ub2 is mostly responsible for later replicating DNA, as outlined in summary figure 6g. Therefore, publishing these results as one paper is critical for drawing a clear conclusion as described above.

1. Introduction, p. 5: the points 2 and 3 listed here are not related to the present work.

According to the reviewer's comment, we removed the points 2 and 3 and revised the text as follows: "However, the existence of two distinct modes of DNMT1 recruitment to hemi-methylation sites, one coupled with and the other uncoupled from DNA replication machinery, has previously been suggested by the previous finding that DNMT1 co-localizes with LIG1 foci in early and mid S-phase, but not in late S-phase." (page 5)

2. p. 6, line 109: The reference to S1a and b is wrong. A figure would be needed that shows the purified protein.

We have now shown the CBB staining of purified recombinant xDNMT1-$Flag_3$ in the revised Supplementary Fig. 1b.

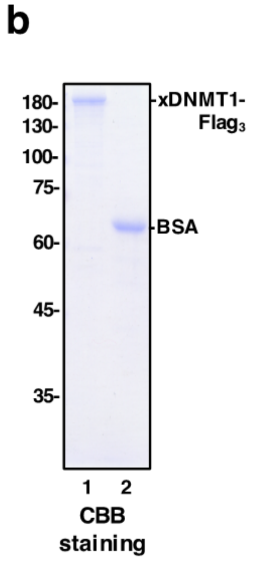

Figure legend

1 μg of recombinant xDNMT1-Flag3 (lane 1), and BSA were separated by SDS-PAGE, and gel was stained with CBB.

3. Fig. S1b: The figure legend is not informative.

According to the reviewer's suggestion, we added detailed information about experiments in the figure legend for revised Supplementary Fig. 1c as follows: "c, Sperm chromatin was added to interphase egg extracts pretreated with UbVS (14 μM) in the presence or absence of free ubiquitin (58 μM). Isolated chromatin fractions were subjected to MNase digestion and solubilized proteins were analyzed by immunoblotting using anti-histone H3 antibody to confirm histone H3 ubiquitylation."

4. Fig. 1C: The relevance of SDS is not explained.

According to the reviewer's comment, we explained the relevance of SDS in the revised text as follows: "rxDNMT1WT specifically interacted with H3Ub2 in denatured chromatin lysates which were treated with 1% SDS and then diluted (Supplementary Fig.1d, +SDS). In native chromatin lysates, rxDNMT1WT interacted with H3Ub2 as well

as with unmodified and mono-ubiquitylated histone H3 (Supplementary Fig. 1d, -SDS), suggesting that indirect binding is also preserved under this condition.” (page 6)

5. p. 7, first paragraph: References for the claims in the first sentenced are missing.

We put the references in the adequate site in the revised version.

6. p. 7: The experimental system of studying DNA replication after addition of sperm chromatin is very special and non-physiological. It needs to be shown that reactions and interaction observed in this system do occur under normal conditions.

In vitro cell free system using *Xenopus* egg extracts is a well-established method for reconstituting DNA replication, cell cycle checkpoints, and mitotic progression. Many papers using this system with an exceptionally high impact have been published and contributed to the advances of many biological fields. The relevance of DNA replication in this in vitro system has already been characterized in many papers. Therefore, we believe that this system is also very useful for analyzing maintenance DNA methylation as shown by our previous publications (Nishiyama et al Nature 2013, Ishiyama et al Mol Cell 2017).

7. p. 7 and Fig. 1b: Contrary to what is stated in the manuscript ubiquitylation levels are not increased after addition of His-Ub. The authors imply that HisUb and Ub lead to a difference in the migration of the ubiquitinated PAF15. This should be confirmed.

We did not mention that the levels of PAF15 ubiquitylation are increased after addition of His-Ub. We used His-Ub, solely for the confirmation that the upshift of PAF15 was due to ubiquitylation.

8. p. 7 and Fig. 1D: Why is the ubiquitylation of wt PAF15 so weak? Why is there stronger ubiquitylation of the single mutants? Can it be excluded that the double mutant protein is misfolded and therefore lacks ubiquitylation?

Dual mono-ubiquitylated PAF15 (wt PAF15) effectively recruits DNMT1 complexed with USP7. In a case with dual mono-ubiquitylated histone H3, we have previously shown that deubiquitylation of histone H3 coupled with conversion of hemimethylated DNA to fully methylated DNA (Yamaguchi et al. Sci Rep 2017). In contrast, single mono-ubiquitylated PAF15 failed to recruit DNMT1 complexed with USP7. Therefore, we speculate that this is also the case with PAF15, showing the apparently weak dual mono-ubiquitylation of PAF15 compared with that of single mono-ubiquitylation. This point is discussed in the revised text (page 11).

9. Fig. 1g: PAF15 input is missing. I do not get why inhibition of deubiquitination should lead to release of PAF15 from chromatin. Indirect effects of UbVs need to be excluded.

We have described this point in the previous version in detail as follows: "To identify factors capable of binding DNMT1 in a ubiquitin signal-dependent manner, we used ubiquitin vinyl sulfone (UbVS) treatment, a pan-deubiquitylation enzyme inhibitor[31], to specifically enrich the accumulation of ubiquitylated proteins in cell-free *Xenopus* extracts. In brief, incubation of *Xenopus* interphase egg extracts with UbVS results in an almost complete depletion of free ubiquitin, leading to the inhibition of both ubiquitylation and deubiquitylation pathways[32]." The treatment of egg extracts with UbVS has already well-established in the previous studies. To further clarify the effect of UbVS, we revised the text as follows: "In brief, pre-treatment of egg extracts with UbVS inhibits ubiquitin turnover and results in an almost complete depletion of free ubiquitin, leading to the inhibition of both ubiquitylation and deubiquitylation pathways[31]". Furthermore, we have shown that addition of Ub in the UbVS-treated extracts selectively activates ubiquitin signals and restores PAF15 and DNMT1 chromatin recruitment (revised Supplementary Fig. 1h), excluding the possibility that the results are a consequence of indirect effects of UbVS. We also show PAF15 input as revised Supplementary Fig. 1g.

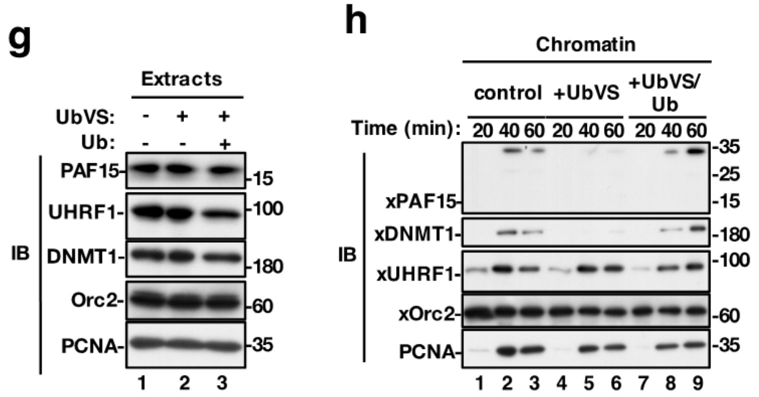

Figure legend

g, Extracts used in Supplementary Fig. 1h were analyzed by immunoblotting using indicated antibodies.

h, Sperm chromatin was replicated in interphase egg extracts containing buffer (lanes 1-3) or 14 μM UbVS in the absence (lanes 4-6) or presence of 58 μM recombinant ubiquitin (lanes 7-9), and chromatin-associated proteins were analyzed by immunoblotting using the indicated antibodies.

10. Fig. 1f and p. 8: To make the claim of a stable complex formation a reciprocal pulldown would be needed as confirmation.

According to the reviewer's indication, we have revised this point in the revised version, showing the results of a reciprocal immunoprecipitation with *Xenopus* PCNA antibody.

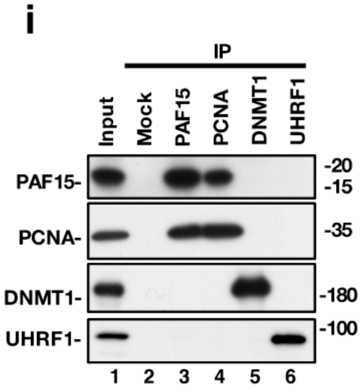

Figure legend

i, Immunoprecipitates from Xenopus interphase egg extracts using anti-xPAF15 (lane 3), anti-xPCNA (lane 4), anti-xUHRF1 (lane 5), and anti-xDNMT1 (lane 6) antibodies, or control IgG (lane 2) as well as egg extracts (lane 1) were subjected to immunoblotting using the antibodies indicated.

12. Fig. 1e and p. 8: It should be noted that the KR mutant also fails to bind to chromatin.

We have now included this information in the revised text.

13. Fig. 3b: PCNA input is missing.

We put PCNA input in the revised Figure. 3b.

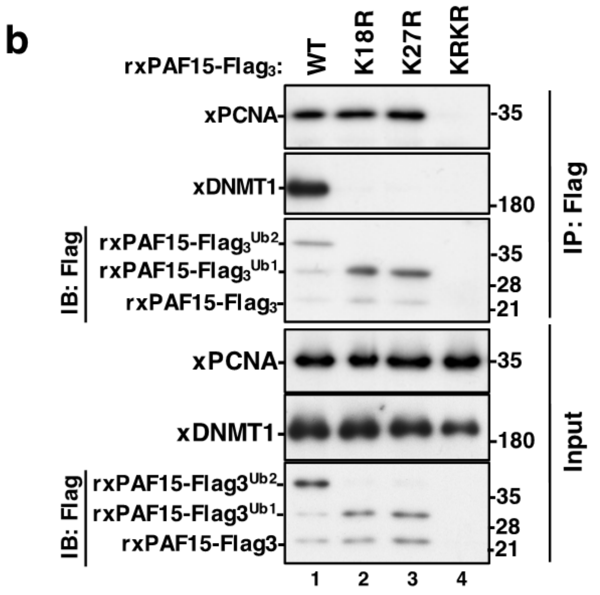

Figure legend

b, Sperm chromatin was replicated in interphase egg extracts containing xPAF15-Flag3 [wild-type, K18R, K27R or K18RK27R (KRKR)]. Isolated and solubilized chromatin proteins were subjected to immunoprecipitation using anti-Flag antibodies. The resultant immunoprecipitates were analyzed by immunoblotting using the indicated antibodies.

14. P. 11: "we prepared a single or mono-ubiquitylated hPAF15" – unclear

We have revised this point in the revised text as follows: "we prepared a single mono-ubiquitylated hPAF15".

15. Fig. 3c: data should be presented together with error margins based on independent

repeats of the experiment. The Kd of 1.4 nM +-0.7 for Ub2 listed on p. 11 line 233 cannot be determined in this setting, because it is covered by only one data point in the titration. Experiments must be repeated at lower protein concentrations or using a different method. The justification for using a two site fit in the case of K24Ub must be given.

This point is important and well taken. Indeed, as the reviewer indicated, we performed the ITC experiments using RFTS and K15Ub/K24Ub shown in the previous Figure 3C under the condition with a bigger c value (c=n[titrand]/Kd: 10000) than that with an optimal value (1<c<1000). Thus, according to the reviewer's suggestion, we repeated the experiments at lower protein concentrations and/or high salt concentrations as the reviewer suggested. Unfortunately, we failed to obtain reliable data due to a low level of calorimetrical reaction even when we used 1/4 protein concentration of what was used in the previous ITC experiments (as shown below). Similar results are frequently observed when the binding affinity is too strong (less than 10 nM)[1].

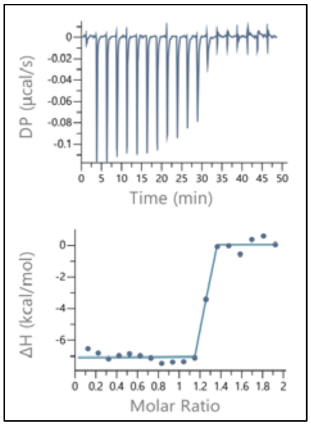

No1:
Cell: 10 µM PAF15Ub2
Syringe: 100 µM RFTS
K_d < 1 nM
Buffer: 20 mM Tris-HCl (pH7.5), 150 mM NaCl, 10µM ZnOAce

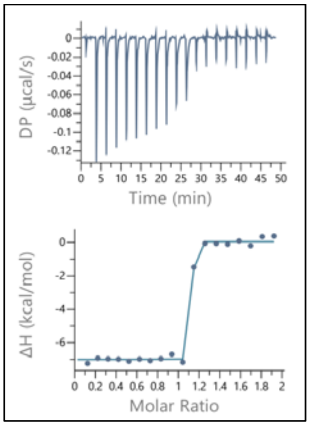

No2:
Cell: 10 µM PAF15Ub2
Syringe: 100 µM RFTS
K_d < 1 nM
Buffer: 20 mM Tris-HCl (pH7.5), 300 mM NaCl, 10µM ZnOAce

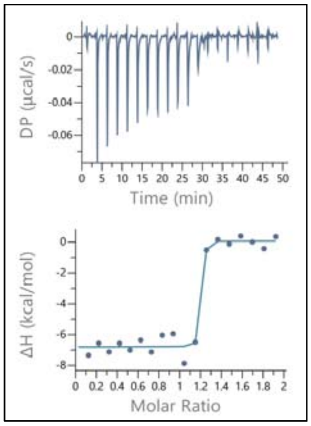

No3:
Cell: 5 µM PAF15Ub2
Syringe: 50 µM RFTS
K_d < 1 nM
Buffer: 20 mM Tris-HCl (pH7.5), 150 mM NaCl, 10µM ZnOAce

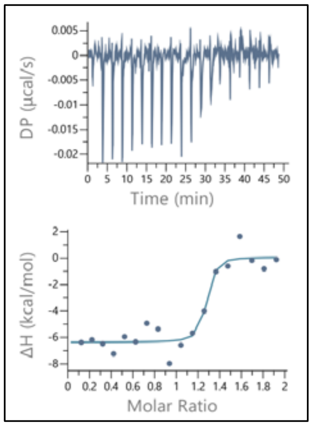

No4:
Cell: 2.5 µM PAF15Ub2
Syringe: 25 µM RFTS
K_d = 2.6 nM
Buffer: 20 mM Tris-HCl (pH7.5), 150 mM NaCl, 10µM ZnOAce

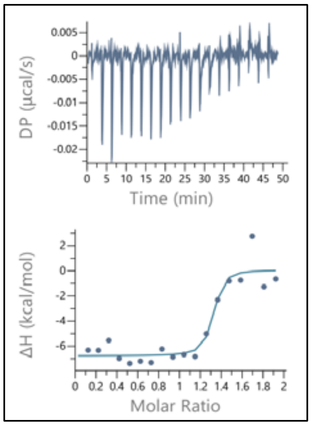

No5:
Cell: 2.5 µM PAF15Ub2
Syringe: 25 µM RFTS
K_d = 3.9 nM
Buffer: 20 mM Tris-HCl (pH7.5), 150 mM NaCl, 10µM ZnOAce

Figure legends

The ITC experiments using low concentration proteins. No1: ITC experiments was performed using 10 µM PAF15Ub2 and 100 µM RFTS which is the same as submitted paper. No2: To reduce the binding affinity, ITC experiment was performed at high salt concentration (300 mM NaCl). No3: A half concentration of original experiment. No4 and 5: ITC data using 2.5 µM PAF15Ub2 and 25 µM RFTS with increased data points at the curve.

1. Padavannil A. *et al.* Importin-9 wraps around the H2A-H2B core to act as nuclear importer and histone chaperone. *Elife.* 8: e43630 (2019).

We then tried to determine the Kd between RFTS and ubiquitylated histone peptides using SPR technology. Again, unfortunately we found that the affinity between RFTS and the histone H3 peptide determined by SPR is far weaker (Kd=30.9 µM) compared to that determined by ITC (Kd=1.3 µM), presumably due to the conformational change of RFTS protein by fixation to the sensor chip. Taken together, we demonstrate the ITC results (c value=10000) in the revised Fig. 3c with standard deviations and describe this in the text as follows: "The ITC experiment using RFTS and K15Ub/K24Ub peptide was performed under a condition with higher c value (c=n[titrand]/Kd: 10000) than that with an optimal value (1<c<1000) because lower concentrations of RFTS protein (even 1/4 of the original) resulted in an insufficient calorimetrical reaction for the reliable detection. Nevertheless, the results indicated that RFTS binds to the K15Ub/K24Ub peptide with much higher affinity than K15Ub or K24Ub." With respect to using a two-site fitting, as the reviewer indicated, the ITC curve derived from binding between RFTS and K24Ub appeared to be complexed and might not be suitable two site fitting, being not capable of determining the exact Kd values. However, the results clearly indicated that RFTS binds to the K15Ub/K24Ub peptide with much higher affinity than K15Ub or K24Ub. Therefore, in the revised version, we demonstrated the Kd values of RFTS/K24Ub as a not determined (n.d.).

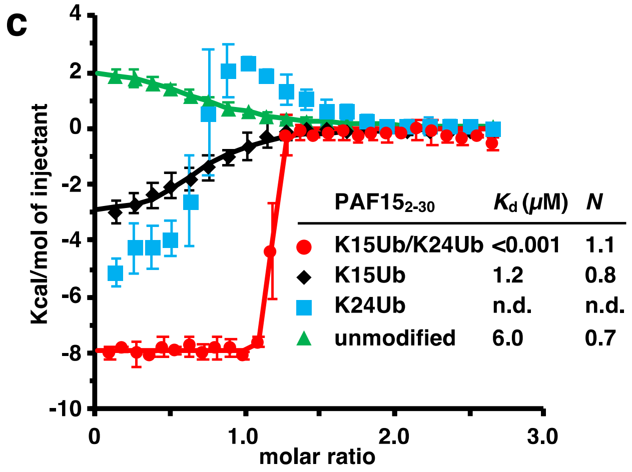

Figure legend

c, Superimposition of plots of enthalpy changes in the interaction between hRFTS and hPAF152-30 or its dual-mono-ubiquitylated form by ITC measurement.

16. p. 11, line 235: Contrary to what is stated in the text, the high binding constant of 3 nM of the K24Ub construct is not far away from the Ub2 construct.

In the revised version, we have shown the binding constant between RFTS and K24Ub as a not determined (n.d.), because the ITC curve appeared to be complexed and might not be suitable two site fitting, being not capable of determining the exact Kd values. However, the results (comparing the curves of RFTS/K15Ub/K24Ub with that of RFTS/K24Ub) clearly indicated that RFTS binds to the K15Ub/K24Ub peptide with a much higher affinity than K15Ub or K24Ub.

17. p. 11 and Suppl. Fig. 3d: The gel filtration profiles should be shown to document that complex formation occurred.

We put the results showing the gel filtration profiles in the revised Suppl. Fig. 3d.

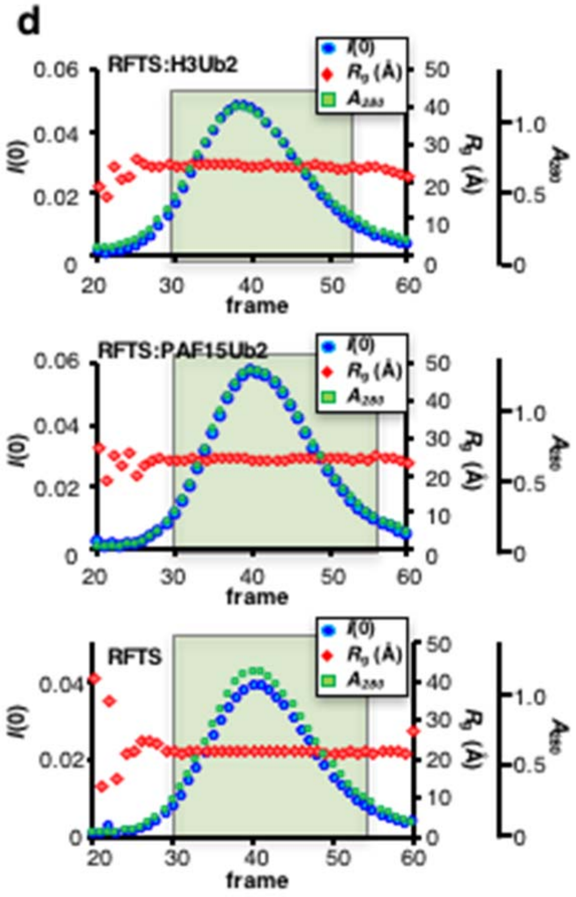

Figure legend

d, R_g (blue), absorption of λ=280 nm (A_{280}: green)and I(0) (red) plots for SEC-SAXS of hRFTS:$H3_{1\text{-}37W}$Ub2 (top), hRFTS:$PAF15_{2\text{-}30}$Ub2 (middle), and RFTS (bottom). X-ray scattering frames highlighted as green were used for extrapolation to zero-concentrations.

18. p. 11-12: The interpretation of the low resolution SAXC data is not convincing and the conclusions at the end of p.11 and beginning of p. 12 are overinterpreted.

According to the reviewer's indication, we have removed the results of *ab initio* models derived from SAXS data.

19. There is no reference to Fig. 3d in the paper.

We put the reference to Fig. 3d in the revised version.

20. Related to the response of the authors that chromatin binding of in-vitro translated recombinant xPAF15 is not reproducible, the figure panels reporting this type of data should be removed.

In the previous version (1st revision), we do not show any results using in vitro translated recombinant proteins.

21. Fig. 4a and d: indicate what the error bars are showing. Specify the number of experiments. Provide p-values.

We have now added short explanation to Fig. 4a and Fig. 4d as follows: "Data are mean ± s.e.m. from three independent experiments." (page 27)

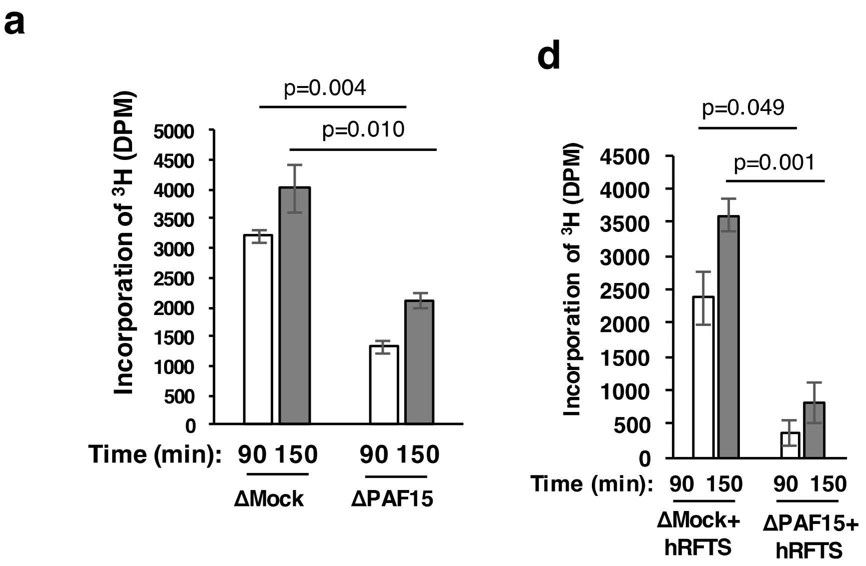

Figure legend

a and d, Sperm chromatin was added to either mock- or xPAF15-depleted extracts containing radiolabelled S-[methyl-^{3}H]-adenosyl-L-methionine in the absence (a) or presence of 0.6 μM hRFTS (e). The efficiency of DNA methylation was measured at the time points indicated. Data are mean ± s.e.m. from three independent experiments.

22. p. 13: The concept of the experiments adding hRFTS to the extracts is not convincing. This is prone to numerous side reactions and indirect effects.

Addition of hRFTS protein in the extract did not affect DNA replication. It also did not apparently affect maintenance DNA methylation in the presence of PAF15 and a defect in the DNA methylation was observed when PAF15 was depleted. Therefore, the results are unlikely due to the indirect effects of hRFTS addition. We have now described this information in the revised version. (page 13)

23. Fig. 4g is interesting. Is this result reproducible?

We repeated this experiment several times and found that they are reproducible. As a reference, we show the other results below.

#2

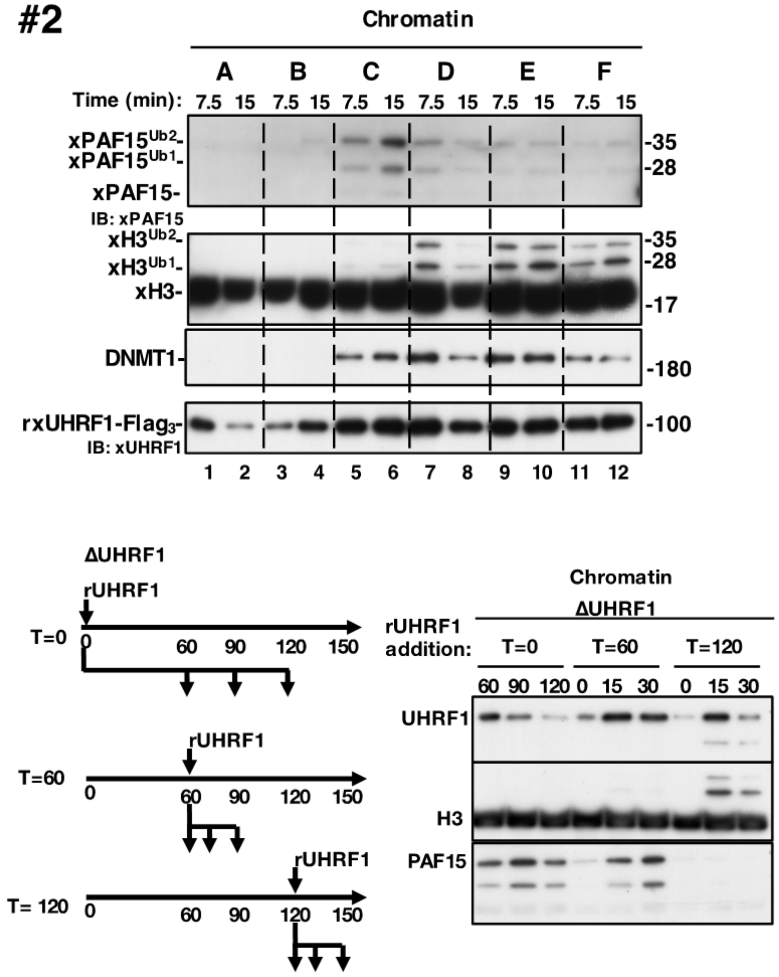

Figure legend

#2: Sperm chromatin was added to xUHRF1-depleted extracts and incubated for 0, 30, 60, 90, 120, or 150 min. Extracts were then supplemented with recombinant xUHRF1-Flag3 and further incubated for 7.5 or 15 min. Chromatin fractions were isolated and chromatin-bound proteins were analyzed by immunoblotting using the antibodies indicated.

Sperm chromatin was added to xUHRF1-depleted extracts supplemented with recombinant xUHRF1-FLAG×3 at 0, 60, or 120 min as indicated in schematic (left). Chromatin-bound proteins were analyzed by immunoblotting using the antibodies indicated.

24. The PAF15 pulldown in Fig. 5a and b is very weak. The PAF15 input is missing in

both panels. It is needed to support the conclusions in the text. These are important data that should be improved.

25. Fig. 5c lacks the necessary input controls.

We agree that input immunoblots are important controls, however, the low cellular PAF15 levels could not be detected in the nuclear extract with the available antibodies. As in our previous work[1], immunoblot detection of mPAF15 in mouse ESCs was only possible after enrichment via immunoprecipitation, which is consistent with the low abundance of mPAF15 protein measured in a recently reported deep-coverage proteome of mouse ESCs[2]. In view of these technical limitations we made every effort to control this experiment. We confirmed equal expression of *Paf15* in all samples by RT-PCR (Supplementary Fig. 5g) and demonstrated equal loading with Ponceau (Fig. 5 a,b). As all samples were meticulously processed in parallel and three times independently reproduced, we find that our results are conclusive, even without the traditional input controls.

To provide an additional line of evidence, we performed the reciprocal experiment and performed the immunoprecipitation with antibodies against PAF15, which allowed us to clearly detect the binding partner DNMT1 also in the input blots (Fig. 5b). Both immunoprecipitation experiments together clearly show that mPAF15 and mDNMT1 interact and that the interaction depends on the ubiquitylation of K15 and K24 residues of mPAF15. Moreover, these data are consistent with the extensive biochemical data presented on the highly conserved *Xenopus* proteins. Most importantly, these data are fully consistent with our functional data that clearly show a crucial role of mPAF15 and its ubiquitylation by mUHRF1 in the recruitment of mDNMT1 and the maintenance of DNA methylation. We revised the text to clarify these issues and also moved supporting, but not essential, data to Supplementary Information.

1. Karg, E. *et al.* Ubiquitome Analysis Reveals PCNA-Associated Factor 15 (PAF15) as a Specific Ubiquitination Target of UHRF1 in Embryonic Stem Cells. *J. Mol. Biol.* **429**, 3814–3824 (2017).

2. Yang, P. *et al.* Multi-omic Profiling Reveals Dynamics of the Phased Progression of Pluripotency. *Cell Syst* **8**, 427–445.e10 (2019).

26. The F3H assay should be based on more than 20 cells. Experiments should be

repeated independently and the results of the independent experiments documented separately not combined. These studies should be done blinded for the experimenter.

We appreciate the reviewer's request to increase the number of cells analyzed within our F3H assay and extended the F3H quantification to a total of 45 cells. We do also acknowledge that the experiment should be performed in independent replicates and apologize that this fact was not sufficiently stated in our last manuscript version. Accordingly, the updated Fig. 5e now depicts the quantification of n=45 cells per sample, derived from three independent experiments (each with n=15). However, we do not agree that documenting the replicates separately would improve the overall message of our assay. On the contrary, it would add even more repetitive figures to the already "large amount of data" present in this manuscript, which was one of reviewer 3's main critiques. Hence, we endeavored to reduce figures and enhance the clarity of the presentation.

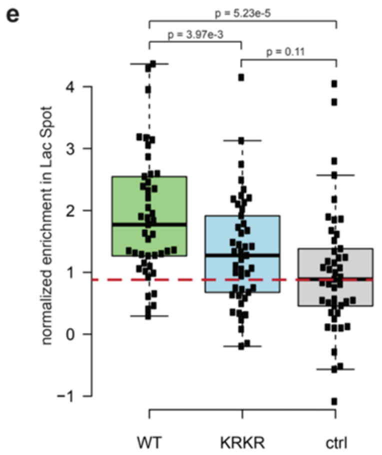

Figure legend

d. Cells were transfected with expression constructs for GFP-Dnmt1, GFPonly, mCherry-Paf15 wild-type (WT) and mCherry-Paf15 K15R/K24R double mutant (KRKR). Line intensity profiles for GFP and mCherry in the respective spots are shown below the confocal images. e. Quantification of the F3H assay. Background subtracted mCherry/GFP ratios within the spots were normalized to the control and plotted with n =45 from three independent replicates (per replicate, n=15). Statistical significance was determined using the student's t-test.

27. Fig. S6a: data are semiquantitative. LC-MS should be done.

We disagree with the reviewer that the immunofluorescence data in Supplementary Figure 6a (revised Supplementary Figure 6c) are semi-quantitative. Rather, our use of high-content image acquisition and analysis allowed us to specifically achieve robust

quantitative fluorescence intensity measurements at single-cell resolution. Thus, the quantification of 5mC for each genotype in Supplementary Figure 6 represents fluorescence intensity measurements from more than 500 cells per biological replicate. These results constitute a convincing and sound second line of evidence for the role of PAF15Ub2 in the maintenance of DNA methylation in mouse ESCs which is wholly consistent with our RRBS data.

Furthermore, we were surprised by the reviewer's suggestion, especially considering that Supplementary Figure 6a has been part of the manuscript in its current form since the original submission and was not singled out for critique in the first round of review. As such, we find the request to perform additional LC/MS measurements inappropriate at this stage of the review process. While we disagree with the notion that LC/MS measurements would be necessary to confirm PAF15Ub2's role in mouse ESCs, the completion of such experiments is also not feasible within the time frame set forth by the editor for these second round of revisions and would unduly delay the publication of our original and timely data.

28. For the RRBS statistics about the coverage and read depth of CpG sites should be provided. Suppl. Table S6 suggests that the read depth per CpG site is not very high and perhaps not sufficient for detailed conclusions. This should include the distribution of the covered CpG sites over genomic elements for the different samples to exclude sampling artefacts.

We appreciate the reviewer's suggestion. We have added the requested coverage statistics to the revised version of Supplementary Table 6. As stated in the Materials and Methods section referring to the RRBS, our analysis was restricted to CpGs with >10x coverage. As such, our conclusions were mainly based on global analyses using the information from a minimum of ~800,000 unique CpGs covered at least 10 times in every sample and should therefore be very robust.

Supplementary Table 6: RRBS Information

Sample_ID	Genotype	# total reads	#mapped reads	% of mapped reads
KRKR_1	PAF15_KRKR_ESC	28,140,727.00	19135694	68
KRKR_2	PAF15_KRKR_ESC	35,670,848.00	26753136	75
WT-1	wt_J1_ESC	33,012,500.00	24429250	74
WT-2	wt_J1_ESC	21,910,914.00	15118530	69
D1KO_r1	DNMT1_KO_ESC	3,686,943.00	2617730	71
D1KO_r2	DNMT1_KO_ESC	3,846,150.00	2499998	65
D1KO_r3	DNMT1_KO_ESC	3,688,377.00	2508097	68
U1KO_r1	UHRF1_KO_ESC	3,248,968.00	2436726	75
U1KO_r2	UHRF1_KO_ESC	3,581,465.00	2399582	67
U1KO_r3	UHRF1_KO_ESC	3,680,511.00	2539553	69
wt_r1	wt_J1_ESC	3,229,351.00	2615775	81
wt_r2	wt_J1_ESC	2,956,850.00	2247206	76
wt_r3	wt_J1_ESC	3,081,518.00	2280324	74

combined coverage of all samples over genomic elements

Genomic Element	coverage [fraction of total]
Repeats	0.013915533
CpG islands	0.610142975
Promoters	0.430718894
Gene bodies	0.445765294

REVIEWERS' COMMENTS:

Reviewer #3 (Remarks to the Author):

I have the impression that the technical comments were addressed sufficiently.
